# A Meta‐Analysis of Conductive and Strong Carbon Nanotube Materials

**DOI:** 10.1002/adma.202008432

**Published:** 2021-07-19

**Authors:** John S. Bulmer, Adarsh Kaniyoor, James A. Elliott

**Affiliations:** ^1^ Department of Materials Science and Metallurgy University of Cambridge 27 Charles Babbage Road Cambridge CB3 0FS UK

**Keywords:** carbon nanotubes, conductive polymers, graphitic intercalation compounds, meta‐analysis

## Abstract

A study of 1304 data points collated over 266 papers statistically evaluates the relationships between carbon nanotube (CNT) material characteristics, including: electrical, mechanical, and thermal properties; ampacity; density; purity; microstructure alignment; molecular dimensions and graphitic perfection; and doping. Compared to conductive polymers and graphitic intercalation compounds, which have exceeded the electrical conductivity of copper, CNT materials are currently one‐sixth of copper's conductivity, mechanically on‐par with synthetic or carbon fibers, and exceed all the other materials in terms of a multifunctional metric. Doped, aligned few‐wall CNTs (FWCNTs) are the most superior CNT category; from this, the acid‐spun fiber subset are the most conductive, and the subset of fibers directly spun from floating catalyst chemical vapor deposition are strongest on a weight basis. The thermal conductivity of multiwall CNT material rivals that of FWCNT materials. Ampacity follows a diameter‐dependent power‐law from nanometer to millimeter scales. Undoped, aligned FWCNT material reaches the intrinsic conductivity of CNT bundles and single‐crystal graphite, illustrating an intrinsic limit requiring doping for copper‐level conductivities. Comparing an assembly of CNTs (forming mesoscopic bundles, then macroscopic material) to an assembly of graphene (forming single‐crystal graphite crystallites, then carbon fiber), the ≈1 µm room‐temperature, phonon‐limited mean‐free‐path shared between graphene, metallic CNTs, and activated semiconducting CNTs is highlighted, deemphasizing all metallic helicities for CNT power transmission applications.

## Graphical abstract

A meta‐analysis of 1304 data points collated over 266 papers statistically evaluates the relationships between carbon nanotube (CNT) material characteristics, at an extrinsic and intrinsic level, and compares them to graphitic intercalation compounds and conductive polymers. An unrivaled deep dive into the state‐of‐the‐art, it provides a roadmap to developing advanced CNT conductors simultaneously competitive with copper and carbon fiber.

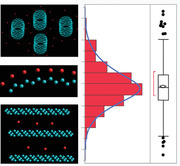

## 1 Introduction

The assembly of carbon nanotube (CNTs) molecules into macroscopic bulk materials is an emerging technology with a breadth of potential electromagnetic applications,^[^ ^1^ ^]^ including high frequency shielding and signal propagation,^[^ ^2^, ^3^, ^4^ ^]^ thermal and mechanical^[^ ^5^ ^]^ energy harvesting, electrodes for electrochemical energy storage,^[^ ^6^ ^]^ flexible transparent touch screens,^[^ ^7^ ^]^ field emission,^[^ ^8^ ^]^ THz and microwave devices,^[^ ^9^ ^]^ and meta‐materials.^[^ ^10^ ^]^ There is also the potential for CNT‐based cables to replace copper and aluminum for electrical power transmission.^[^ ^11^, ^12^, ^13^, ^14^, ^15^, ^16^, ^17^ ^]^ The concept of a high conductivity, high strength electrical cable based on sp^2^ carbon has been considered for almost forty years,^[^ ^18^ ^]^ well before the widespread recognition of CNTs as fullerenes, and now a great variety of advanced CNT‐based materials contend for this application; examples include aligned bucky paper,^[^ ^19^, ^20^ ^]^ aligned wet or dry spun CNT fiber,^[^ ^11^, ^21^ ^]^ and CNT–metal hybrid composites.^[^ ^22^, ^23^ ^]^ While all these materials are based on CNTs, not all CNT materials are created equally and there is a spectrum of performance trade‐offs between cost, conductivity, strength, stability, purity, degree of doping, crystallinity, and production yield. These metrics depend on not just the quality and structure of the individual CNTs, but how the CNTs assemble into an extrinsic microstructure. Currently, the best CNT‐based bulk cables are on a par or better than conventional synthetic fibers in terms of strength.^[^ ^24^, ^25^, ^26^, ^27^, ^28^ ^]^ In terms of electrical conductivity, they barely exceed the conductivity of iron^[^ ^29^ ^]^ (the typical catalyst used in CNT growth) and are a factor of six lower than that of copper.

**Figure 1:**
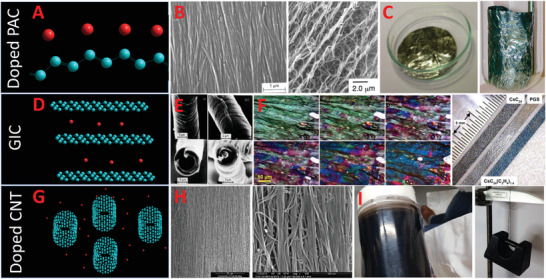
The various advanced carbon conductors that compete with traditional metallic conductors. Top row: A–C) Iodine‐doped polyacetylene as shown with a model (A), SEM images (B), and visual photographs (C) showing their shiny nature. Left SEM image in (B): Reproduced with permission.^[^ ^85^ ^]^ Copyright 1998, Elsevier. Right SEM image in (B): Reproduced with permission.^[^ ^86^ ^]^ Copyright 1998, American Association for the Advancement of Science (AAAS). Left photograph in (C): Reproduced under the terms of the CC‐BY Creative Commons Attribution 3.0 Unported license (https://creativecommons.org/licenses/by/3.0).^[^ ^87^ ^]^ Copyright 2013, IOP Publishing Ltd. Right photograph in (C): Reproduced with permission.^[^ ^88^ ^]^ Copyright 2010, RSC. Middle row: D–F) Graphitic intercalation compounds as shown with a model (D), SEM images (E) of graphitized carbon fiber before doping, and visual photographs (F) showing the color change. Photos in (E): Reproduced with permission.^[^ ^89^ ^]^ Copyright 1982, IOP Publishing. Left photos in (F): Reproduced with permission.^[^ ^90^ ^]^ Copyright 2019, American Chemical Society. Right photo in (F): Reproduced with permission.^[^ ^50^ ^]^ Copyright 2012, Elsevier. Bottom row: G–I) Doped SWCNT bundles as shown with a model (G), SEM images (H), and visual photographs (I). H,I) Photos in (H) and (I): Reproduced with permission. Copyright Dr. Thurid Gspann.

Notably, the absolute conductivity of older carbon‐based bulk conductors, such as conductive polymers and graphitic intercalation compounds (GICs), approached and exceeded the room temperature conductivity of copper in the late 1970s and 1980s (**Figure** **1**).^[^ ^30^, ^31^, ^32^, ^33^, ^34^, ^35^, ^36^, ^37^, ^38^ ^]^ The archetypal example of a conductive polymer is iodine‐doped polyacetylene—an assembly of 1D chains of sp^2^ carbon atoms with typically electron acceptor dopants, such as iodine, residing interstitially between carbon chains. The most conductive polymers reached conductivities of 11 to 15 MS m^−1[^ ^30^, ^39^ ^]^ (compared to 100% IACS copper at 58 MS m^−1^), and had to be kept in air‐free containment vessels to maintain their conductivity. A graphitic intercalation compound is some form of graphite, usually single‐crystal or graphitized carbon fiber, that is doped typically with electron acceptor chemical species residing between graphene planes in ordered, stacked stages. Their highest reported conductivities reached 16^[^ ^40^ ^]^ to 20^[^ ^41^ ^]^ to 35^[^ ^42^ ^]^ to 63^[^ ^33^, ^43^ ^]^ to 90 MS m^−1[^ ^44^ ^]^ for highly crystalline graphite and 16.7^[^ ^45^ ^]^ to 33^[^ ^46^ ^]^ to 36^[^ ^47^ ^]^ to 90 MS m^−1[^ ^37^ ^]^ for highly crystalline vapor‐grown carbon fiber across multiple research groups. These graphitic intercalation compounds were small in size (square millimeters for single‐crystal graphite and centimeter‐lengths for vapor‐grown carbon fiber) and discontinuous, mechanically impractical (tending to break or kink if bent too far) and unstable in air. Furthermore, most reports were well below these high conductivity values.^[^ ^18^, ^32^, ^48^ ^]^ By 1989, later studies showed graphitic interaction compounds exhibited stable electrical conductivity for years in air^[^ ^32^, ^40^, ^47^, ^49^, ^50^, ^51^, ^52^, ^53^, ^54^ ^]^ and strengths comparable to the host carbon fiber,^[^ ^18^, ^45^, ^55^, ^56^ ^]^ although their conductivity generally never exceeded ≈10 MS m^−1^ (At least one paper^[^ ^45^ ^]^ reported materials with strength of carbon fiber and a conductivity of 16 MS m^−1^). Conductive polymers and graphitic intercalation compounds were considered a way to explore the transport of 1D and 2D systems respectively, well before the widespread recognition of CNTs and graphene.^[^ ^42^, ^57^ ^]^ Considering that an individual CNT's intrinsic 1D electrical transport is potentially superior to the intrinsic electrical conductivity of these previously investigated carbon morphologies that have already reached the level of copper conductivities, further development of a bulk CNT conductor is warranted.

Tailored for the practical development of strong and conductive CNT materials, this meta‐analysis surveys a wide body of CNT, conductive polymer, and graphitic interaction compound literature to statistically compare characteristics of these materials at an extrinsic and intrinsic level. Metrics are catalogued in a database provided in the Supporting Information section with references. Composite CNT materials such as CNTs combined with polymers, metals, or other matrices will largely not be considered. The paper is organized as follows: in Section 2, we first review the mesoscopic, intrinsic characteristics of CNTs with details potentially overlooked in the bulk CNT textile community to include transport in: Section 2.1 metallic single‐wall CNTs (SWCNTs); Section 2.2 semiconducting SWCNTs; Section 2.3 multi‐wall CNTs (MWCNTs); Section 2.4 CNT bundles; Section 2.5 CNT junctions. This answers the question of how an assembly of CNTs (forming a mesoscopic bundle and then forming a macroscopic cable) compares to the assembly of graphene (forming into single‐crystal graphite crystallites and then into macroscopic graphite or carbon fiber). Next, we discuss the meta‐analysis on the aggregation of data across the literature on the experimentally reported extrinsic characteristics of CNT materials to include: Section 3.1 bulk conductivity, strength, and multi‐functionality; Section 3.2 thermal conductivity and ampacity; Section 3.3 density and specific properties; Section 3.4 purity; Section 3.5 anisotropy and microstructure alignment; Section 3.6 average CNT dimensions and graphitic perfection; Section 3.7 intercalation doping and junction enhancement. In Section 4, we integrate the previous topics together with: Section 4.1 partitioning of intrinsic and extrinsic characteristics in CNT material; Section 4.2 leading properties of the ultimate CNT material; Section 4.3 CNT's analogy to graphene and single‐crystal graphite; Section 4.4 briefly mentions CNT–metal composites for context; and Section 5 concludes with a summary of the meta‐analysis findings and the path ahead. Meta‐analysis methods are provided in detail at the end.

Briefly in regards to meta‐analysis methods, for each CNT category (unaligned multi‐wall, aligned multi‐wall, unaligned few‐wall, aligned few‐wall), a quantitative comparison is made between two CNT material properties from an amalgamation of papers on a log‐log chart using Pearson correlation coefficients and their corresponding *p*‐values. *p*‐Values equal to or less than 0.05 are deemed to indicate a statistically significant correlation and are colored green; *p*‐values between 0.05 and 0.1 are deemed to indicate weaker significance and are colored grey. A fitted slope with standard error is also provided; this represents the exponent in the power‐law associated between the two properties. Different studies provide different numbers of data points, possibly biasing the correlation. We also consider a weighted correlation and power‐law fit that assigns an equal weight to each academic paper independent of the number of data points provided for that particular CNT category. Correlations and fitted power‐laws that persist from the unweighted to the weighted analysis strengthen the notion that the relationship exists across the literature opposed to being associated with a few numerically influential studies.

There are many useful literature reviews on CNT‐based materials, including general review of CNT cables^[^ ^1^, ^11^, ^58^ ^]^ and transparent conductive films,^[^ ^59^ ^]^ mechanical properties and composites,^[^ ^60^, ^61^ ^]^ metal CNT composites,^[^ ^23^ ^]^ thermal conductivity and ampacity,^[^ ^62^ ^]^ thermoelectrics,^[^ ^63^ ^]^ fiber production,^[^ ^20^, ^21^, ^64^, ^65^, ^66^, ^67^ ^]^ transport mechanisms and intrinsic properties,^[^ ^68^, ^69^, ^70^, ^71^, ^72^ ^]^ armchair CNTs,^[^ ^73^ ^]^ double‐wall CNTs (DWCNTS),^[^ ^74^, ^75^ ^]^ electronic and helicity sorting,^[^ ^76^ ^]^ and doping.^[^ ^77^ ^]^ There are also useful reviews on carbon fiber,^[^ ^78^ ^]^ graphitic intercalation compounds,^[^ ^32^, ^35^, ^79^, ^80^, ^81^ ^]^ conductive polymers,^[^ ^82^, ^83^ ^]^ and Lewis super‐acids.^[^ ^84^ ^]^ This paper is noteworthy in that, for the first time, a comprehensive meta‐analysis collates CNT material properties extensively across the literature and statistically analyses trends across these studies; further, the review on intrinsic properties uniquely organizes and highlights exactly how CNTs are superior to other carbon‐based materials for a macroscopic multifunctional material. Our statistical analysis is performed on a database with 1304 data points accumulated from 266 academic papers on CNTs, graphitic intercalation compounds, and conductive polymers. Our statistical analysis thus far broadly covers many material topics at the simple level of univariate analysis, but may miss trends obscured in the ultimately multidimensional parameter space. We envision later studies that can address specific material topics in more detail, such as multidimensional modeling. The database is provided in the Supporting Information section. We would welcome other interested research groups to explore and add to this database.

## 2 Intrinsic Characteristics

A doped CNT cable could be superior to graphitic intercalation compounds and conductive polymers due to its unique intrinsic transport properties. To estimate the upper‐bound bulk conductivity and compare this against the older carbon‐based conductors, we first review the experimentally measured intrinsic transport properties of various individual electronic species of CNT molecules (metallic, semiconducting, multi‐wall) and their next level of structure (bundles and junctions).

Typical SWCNT diameters range from 0.7 to 4.0 nm; for larger diameters, they can undergo radial collapse.^[^ ^91^ ^]^ The length of SWCNTs in bulk assemblies typically ranges from micrometers to millimeters.^[^ ^92^ ^]^ Individually, a SWCNT may span tens of centimeters^[^ ^93^, ^94^ ^]^ with the longest reaching beyond half a meter.^[^ ^95^ ^]^ The geometric twist in the tubular lattice, called helicity (sometimes mistakenly referred to as chirality), dictates its electronic properties. A simple tight‐binding model can be used to approximate an individual CNT's electronic properties by simply considering a graphene lattice with periodic boundary conditions; in this simple picture, there is no regard to curvature‐induced straining of the sp^2^ bonds from rolling up the graphene sheet into a nanotube. It predicts two‐thirds of all possible SWCNT structures are twisted in a manner that makes them semiconducting with bandgap 0.7 eV/*d*,^[^ ^96^, ^97^, ^98^ ^]^ where *d* is the numerical value of nanotube diameter in nanometers. The remaining one‐third of SWCNTs are called “metallic” and were initially predicted to not have a bandgap, although experiments have gradually eroded this notion.

In 2001, researchers^[^ ^99^ ^]^ showed that most SWCNTs deemed metallic by the tight‐binding model actually had a diameter‐dependent bandgap 0.03 to 0.08 eV which was attributed to lattice curvature. Individual SWCNTs with an exact helical angle θ = 30°, called armchair, were shown in that study to be gapless. However, in 2009, transport experiments were carried out on very clean metallic SWCNTs suspended from the supporting substrate like a bridge, so that the metallic SWCNT was completely isolated.^[^ ^98^, ^100^, ^101^, ^102^ ^]^ These studies found that all metallic SWCNTs when completely isolated, including the armchair category, had a surprisingly large bandgap of 0.15 to 0.30 eV (following 0.45 eV/*d*) with no dependence on helical angle. It was concluded that no SWCNT is intrinsically metallic. The bandgap stems from interaction between charge carriers, although the exact nature is an active area of research. Still, even without conditions of extreme isolation, only ≈1% of individual SWCNTs incorporated into transistor devices act as truly metallic with a conductance completely independent of the transistor's gate voltage. Most SWCNTs incorporated into a transistor device either have either a “narrow‐band” response where there is at least partial suppression of current with gate voltage, or full on/off semiconducting behavior.^[^ ^98^, ^103^ ^]^ Nevertheless, for the purposes of this discussion, and following common convention in the literature, we will refer to the “narrow‐band” and “quasi‐metallic” SWCNTs as “metallic.”

### 2.1 Metallic SWCNTs

Metallic SWCNT are relevant to advanced carbon‐based conductors because they uniquely support extended delocalized electronic wave functions in one dimension, even with the presence of disorder.^[^ ^104^, ^105^ ^]^ It is not immediately obvious, however, that 1D transport is advantageous to achieving a high conductivity electrical cable for two reasons. First, it was thought that 1D transport would be hampered by the Peierls instability. Common to conductive polymers and other 1D metals, the Peierls instability stems from the breaking of 1D crystal symmetry by atomic oscillations along the 1D chain.^[^ ^105^ ^]^ This necessitates chemical doping of 1D polyacetylene to achieve a transition from an insulating to conductive state. The Peierls instability does not practically affect SWCNTs^[^ ^70^, ^104^, ^105^, ^106^ ^]^ because the stiffness of the sp^2^ bonds makes the bandgap of Peierls instability in SWCNTs very small (with bandgap 0.002 eV/*d* ^3^).^[^ ^100^ ^]^ This means that doping is not required to make a quasi‐1D metallic SWCNT conductive. Second, charge carrier maneuvering around a defect in 1D transport is by definition not possible and so it was thought any degree of disorder on a 1D chain would lead to charge carrier localization. For conductive polymers, this localization is not necessarily a major difficulty provided that charge carriers can hop to an adjacent chain before reaching a defect. For a well‐ordered 1D assembly, the condition of bulk electronic transport of 1D conductors is met when *L*_coherence_/*a* >> *t*_0_/*t*_3d_ where *L*_coherence_ is the charge carrier coherence length, *a* is the chain repeat unit length, *t*_0_ is the intrachain π‐electron transfer integral, and *t*_3d_ is the interchain π‐electron transfer integral.^[^ ^107^ ^]^ Sensitivity to any disorder is not a concern for quasi‐1D metallic SWCNTs; charge carriers exist as doughnut‐shaped standing waves around the CNT circumference where a point defect is instead incorporated into a collective circumferential average. Thus, a point defect in a metallic SWCNT uniquely does not result in automatic localization, but instead a less severe region of increased scattering probability.^[^ ^104^ ^]^ Because a larger circumference averages out a point defect, the characteristic distance between scattering from defects (the elastic mean‐free‐path *L*_m(Elastic)_) on a metallic SWCNT is proportional to the diameter (*L*_m(Elastic)_ *∝ d*).^[^ ^104^, ^108^, ^109^ ^]^ This relationship is expected to persist with substrate interaction, CNT bending, and charge carrier interaction,^[^ ^109^ ^]^ as well as with semiconducting SWCNTs.^[^ ^104^ ^]^ While large diameter implies a large mean‐free‐path, this trend will not continue indefinitely to where either the SWCNT undergoes radial collapse (expected for diameters over 4 nm^[^ ^91^ ^]^) or the transport transitions from 1D to 2D, becoming more graphene‐like. A computational study shows that the electronic mobility in CNTs converges to graphene's value around a diameter of 3 to 10 nm at room‐temperature, depending on the carrier concentration.^[^ ^110^ ^]^ Also, generally, a CNT's bandgap grows inversely with diameter and by 10–15 nm there is metallic behavior at room‐temperature independent of electronic species.^[^ ^111^, ^112^ ^]^ In terms of the tight‐binding model, the insensitivity to defects is shown as a linear (i.e., dispersionless) first conduction band, meaning charge carriers travel like massless particles, a feature shared with graphene.^[^ ^103^, ^113^ ^]^ Metallic SWCNTs also have additional semiconducting dispersive sub‐bands that contribute after substantial doping or electrostatic activation (with a bandgap of 2.5 eV/*d*).^[^ ^114^ ^]^

**Figure 2:**
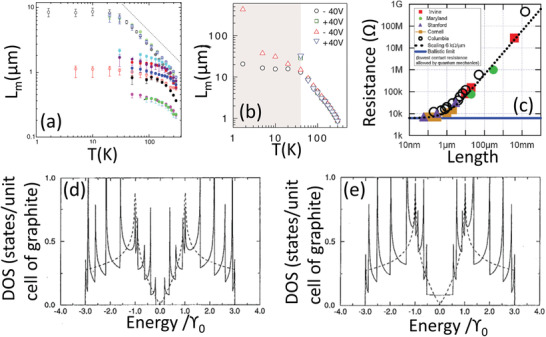
Transport characteristics of individual CNTs and graphene. a) Mean‐free‐path (*L*_m_) versus temperature (*T*) for a metallic SWCNT (open circles) and activated semiconducting SWCNTs (closed circles). Dotted straight line indicates *T* ^−1^ dependence, the expected behavior for electron–phonon scattering. Reproduced with permission.^[^ ^103^ ^]^ Copyright 2007, American Physical Society. b) Graphene has a similar room‐temperature mean‐free‐path as the SWCNTs, which also increases dramatically at colder temperatures. Reproduced with permission.^[^ ^122^ ^]^ Copyright 2013, AAAS. c) Compilation of studies showing resistance versus length of long individual SWCNTs, to include activated semiconducting SWCNTs. Resistance per length falls on a master curve at ≈6 kΩ µm^−1^. Reproduced with permission.^[^ ^69^ ^]^ Copyright 2009, Wiley‐VCH. d,e) Density of states of a semiconducting (10,0) SWCNT (d) and a metallic (9,0) SWCNT (e). *ϒ*_0_ is the nearest neighbor overlap integral; dotted lines are the density of states of graphene. d,e) Reproduced with permission.^[^ ^123^ ^]^ Copyright 1992, The American Institute of Physics.

With defect‐induced 1D localization and the Peierls instability both uniquely mitigated in metallic SWCNTs, as well as their particular dispersionless insensitivity to defects that grows with *d*, the fact that the transport is 1D is now advantageous. This is because the extreme anisotropy suppresses phonon or charge carrier backscattering—leading to long mean‐free‐paths *L*_m_, from both elastic and inelastic collisions, at room‐temperature.^[^ ^71^ ^]^ Individual metallic SWCNTs have room‐temperature mean‐free‐path lengths *L*_m_ experimentally measured on the order of 1 µm.^[^ ^71^, ^115^, ^116^, ^117^ ^]^ Both computational modeling^[^ ^69^ ^]^ and experiments^[^ ^70^, ^103^ ^]^ have determined that the room‐temperature mean‐free‐path is largely, though not completely, limited by inelastic acoustic phonon collisions. This is also illustrated in **Figure** **2**a for individual ultralong (1 mm) SWCNTs of metallic (open circles) and semiconducting (filled circles) varieties where the general mean‐free‐path *L*_m_ is measured as a function of temperature.^[^ ^103^ ^]^ Not just for the metallic variety, but for the semiconducting CNTs as well, *L*_m_ is ≈1 µm at room‐temperature. It is expected that an ideal, defect free metallic SWCNT will always have a room‐temperature mean‐free‐path, limited by unavoidable acoustic phonon collisions, on the order of 1 µm.^[^ ^69^ ^]^ For this reason, an infinitely long and perfectly crystalline metallic SWCNT at room‐temperature would not approach the conductivity of a superconductor. At room‐temperature, the ≈1 µm mean‐free‐path is well beyond both traditional metals (≈10 nm), doped polyacetylene (≈100 nm^[^ ^118^ ^]^), graphite (≈235 nm^[^ ^119^ ^]^), and graphitic intercalation compounds (≈100 nm^[^ ^120^ ^]^). The micrometer mean‐free‐path of 1 µm for a metallic CNT is approximately equal to that in graphene at room‐temperature (Figure 2b). Below ≈30 K, the mean‐free‐path in metallic SWCNTs increases dramatically relative to the semiconducting SWCNTs and indicates a metallic SWCNT's dispersionless behavior. The mean‐free‐path can approach 10 µm and become temperature‐independent, signaling that elastic collisions with crystal defects, opposed to phonons, now exclusively limit the transport. Figure 2b shows that graphene responds similarly with temperature.

For SWCNTs with lengths *L* much smaller than the mean‐free‐path *L*_m_ (*L* << *L*_m_), the SWCNT is a non‐Ohmic ballistic conductor where the resistance, independent of length, is always (2*G*_0_)^−1^ ≈ 6.5 kΩ (where *G*_0_ = 2*e* ^2^/*h* is the conductance quantum, *e* is the electric charge, and *h* is Planck's constant^[^ ^71^, ^121^ ^]^). For SWCNTs of length *L* much larger than *L*_m_ (*L* >> *L*_m_), the resistance is Ohmic with resistance *R* scaling with *L*_m_^[^ ^103^ ^]^ according to

(1) $$\begin{array}{c} R(L)=\frac{1}{2G_{0}}\left(1+\frac{L}{L_{m}}\right) \end{array}$$

The large mean‐free‐path enables very large sustainable current densities and conductivities in metallic SWCNTs.^[^ ^121^ ^]^ One experiment injected ≈25 µA into a ≈1 nm wide metallic SWCNT, which corresponds to a current density of ≈10^13^ A m^−2^.^[^ ^124^ ^]^ This is well beyond the limit for high temperature superconductors in liquid nitrogen (≈10^11^ A m^−2^) and two to three orders of magnitude beyond the electromigration limit of copper.^[^ ^121^ ^]^ In terms of conductivity, the conductivity of a thin cylinder scales inversely with the square of its diameter. Even allowing for the complication that elastic mean‐free‐path *L*_m(Elastic)_ scales linearly with CNT diameter, it would be expected that the standard conductivity should still correlate negatively with CNT diameter. Over many experiments it has been shown that long individual SWCNTs (up to ≈1 cm) measured in their Ohmic region (*L* >> *L*_m_) fall on an approximate master curve of ≈6 kΩ µm^−1[^ ^69^, ^115^ ^]^ with some spread ranging from ≈4^[^ ^125^ ^]^ to ≈10 kΩ µm^−1[^ ^103^ ^]^ (Figure 2c). Note that ≈6 kΩ µm^−1^ is what is expected if Equation (1) is applied with an *L*_m_ of 1 µm, which is the approximate maximum value of the mean‐free‐path limited by room‐temperature phonons. Assuming a 1 nm diameter metallic SWCNT, ≈6 kΩ µm^−1^ translates to a conductivity of 200 MS m^−1^. This is 3.5 times greater than copper^[^ ^69^, ^71^, ^115^, ^125^ ^]^ and approximately the estimated intrinsic limit of doped polyacetylene.^[^ ^118^ ^]^ In one study, a wider SWCNT with diameter 1.65 nm was found to have a conductivity of 67 MS m^−1^, only slightly above the value of copper. While the 67 to 200 MS m^−1^ intrinsic range does not incorporate doping, this range covers the best measured intrinsic CNT conductivities and are an upper bound estimate of the extrinsic conductivity of a CNT based cable.

### 2.2 Semiconducting SWCNTs

A semiconducting SWCNT can be as conductive as a metallic SWCNT at room‐temperature, provided that there is sufficient chemical doping or electrostatic switching.^[^ ^71^ ^]^ Shown in Figure 2a, at liquid helium temperatures, the mean‐free‐paths of the metallic SWCNTs are nearly three to ten times greater than activated semiconducting SWCNTs.^[^ ^103^ ^]^ This illustrates that doped semiconducting SWCNTs are more sensitive to defects than metallic SWCNTs, because of the metallic SWCNTs’ dispersionless conduction band. However, at room‐temperature, when inelastic phonon scattering is significant, the distinction between metallic and doped semiconducting SWCNTs is not so distinct and the mean‐free‐path in both is ≈1 µm.^[^ ^103^ ^]^ Other studies on activated semiconducting SWCNTs^[^ ^126^, ^127^, ^128^, ^129^, ^130^ ^]^ show room‐temperature mean‐free‐paths similar to metallic SWCNTs (on the order of 1 µm) and others^[^ ^96^, ^128^, ^131^ ^]^ showed the same very large current densities (≈10^13^ A m^−2^). Intrinsic room‐temperature mobilities of individual semiconducting SWCNTs span from ≈10^4[^ ^132^ ^]^ to ≈10^5^ cm^2^ V^−1^ s^−1^,^[^ ^129^ ^]^ compared to 13 × 10^3^ cm^2^ V^−1^ s^−1^ for graphite.^[^ ^32^, ^79^ ^]^ At room‐temperature it has been found that activated semiconducting SWCNT also fall on or near the ≈6 kΩ µm^−1^ master curve^[^ ^115^, ^127^ ^]^ and consequently may have a room‐temperature conductivity comparable to metallic SWCNTs^[^ ^129^ ^]^ when sufficiently doped.

Figure 2d,e shows the density of states of two representative metallic and semiconducting SWCNT respectively, both with approximately the same diameter superimposed in the density of states of graphene. Because the transport is restricted to less than three dimensions, spikes in the density of states appear, called van Hove singularities, for graphene and the two nanotubes. Electronic transitions between these spikes are responsible for helicity selective absorption of SWCNTs in the visible spectrum. Doping to shift these spikes onto the Fermi level leads to step increases in the carrier density. Considering that at room‐temperature, graphene, metallic SWCNTs, and activated semiconducting SWCNTs have approximately the same mean‐free‐path of 1 µm, differences in conductivity will become apparent with carrier density and ease of doping. Figure 2d,e shows the energy shift between the van Hove singularity and the Fermi level is much smaller for semiconducting SWCNTs than it is for metallic SWCNTs and graphene, for a given diameter.

### 2.3 Multi‐Wall CNTs

Sometimes referred to as the ultimate carbon fiber,^[^ ^106^ ^]^ MWCNT are CNTs concentrically nested within larger CNTs. The outer diameters may span 2 to 30 nm^[^ ^106^ ^]^ with lengths ranging from micrometers to centimeters; they can be wide enough that 1D effects are not significant and are metallic without a bandgap.^[^ ^1^, ^111^ ^]^ Computational calculations show that the electronic mobility of all varieties of CNTs converge to 2D graphene somewhere between a diameter of 3 to 10 nm depending on temperature and carrier density;^[^ ^110^ ^]^ MWCNTs wider than 10–15 nm have a sufficiently small bandgap that they are metallic at room‐temperature.^[^ ^111^, ^112^ ^]^ The spacing between shells in a MWCNT span between 0.34 and 0.39 nm and gets larger with smaller CNT diameter,^[^ ^133^ ^]^ compared to the 0.34 spacing between planes in graphite.^[^ ^134^ ^]^ MWCNTs can have diffusive transport^[^ ^135^ ^]^ or, similar to graphene and SWCNTs, can sometimes have dispersionless, ballistic conduction with longer room‐temperature mean‐free‐paths stretching up to ≈2 µm and greater.^[^ ^112^, ^136^ ^]^ Measured individual MWCNT room‐temperature conductivities are sometimes low (0.1–0.8 MS m^−1[^ ^137^, ^138^, ^139^, ^140^ ^]^) and, in cases of high graphitic perfection, were over an order of magnitude higher 8–20 MS m^−1^.^[^ ^139^, ^141^ ^]^ This range is greater than single‐crystal graphite and as‐is SWCNT bundles, although below copper and individual metallic SWCNTs.

The varied transport responses are a result of different CNT qualities, as well as the intricate interactions between inner shells of the MWCNT.^[^ ^142^ ^]^ Typically, inner shell stacking is incommensurate; this sets a non‐periodic potential on conducting shells and generates an increase in defect density.^[^ ^142^ ^]^ Also, it has largely been found that electronic transport is 2D, like graphene, and takes place only on the surface of the outermost two shells. This prevents the full cross section from contributing to the conductivity,^[^ ^106^ ^]^ unless special considerations are taken to electrically connect all the shells.^[^ ^112^, ^135^ ^]^ One study reports Ohmic connections to all the internal shells of a MWCNT by suspending it vertically between two metallic surfaces. Here, all the concentric CNTs within the multi‐wall CNT are connected and participate in the transport, which contrasts with the more typical situation where electrical contacts are made on the CNTs outer surface. This fully connected configuration leads to the highest CNT conductance measured, ≈230 times the theoretical ballistic conductance of an individual metallic SWCNT.^[^ ^112^ ^]^ The temperature dependence of resistance of an individual MWCNT is semiconductor‐like, where resistance increases with decreasing temperature; this is opposite to that found in metallic SWCNTs and traditional metals.

An important subset of the MWCNT variety is DWCNTs. With outer diameters ranging from 1.9 to 5 nm and an intershell spacing 0.33–0.41 nm, the outer shell is small enough to act as a 1D conductor with van Hove singularities and radial breathing modes.^[^ ^74^, ^75^ ^]^ Due to their larger curvature, DWCNTs are more rigid, chemically inert, and oxidize at higher temperatures compared to SWCNTs. DWCNTs have a total of four electronic species combinations, depending on the metallic or semiconducting nature of the inner and outer shells.^[^ ^74^, ^75^ ^]^ If the inner and outer shells are commensurate in registry and sufficiently close, modeling results indicate that the overall electronic nature is determined by both intrashell electronic species, as well as now the intershell coupling.^[^ ^74^ ^]^ Commensurate DWCNTs are easier to simulate computationally, although are rarely found in the real world.^[^ ^74^ ^]^ Multiple field effect transistor experiments on individual DWCNTs, assumed to be incommensurate, found that a semiconducting shell within a semiconducting shell behaved like a semiconducting SWCNT; with just a metallic outer shell, the DWCNT behaved like a metallic SWCNT; and in the particular case of a metallic shell within a semiconducting shell, there was weak gate voltage dependence because the inner metallic shell shields the gate voltage to the outer semiconducting shell.^[^ ^75^, ^143^, ^144^ ^]^ It has been shown that the inner shell can be electrically active where both shells contribute similarly to the transport.^[^ ^145^, ^146^, ^147^ ^]^ For this reason, DWCNTs are better intrinsic conductors than large MWCNTs (in which not all shells are coupled) and possibly better than SWCNTs,^[^ ^148^, ^149^, ^150^ ^]^ while maintaining a high degree of chemical stability.^[^ ^74^ ^]^ Further, functionalization can happen exclusively on the outer shell, leaving the inner tube intact and isolated.^[^ ^146^, ^151^ ^]^

### 2.4 CNT Bundles

SWCNTs and DWCNTs typically self‐assemble into aligned bundles though van der Waals forces and other interactions; typical bundles contain a few, to dozens, to hundreds of CNTs separated by a spacing of ≈0.34 nm. Bundle diameters range anywhere from 2 to 200 nm wide with lengths tens to hundreds of micrometers.^[^ ^150^, ^152^, ^153^, ^154^ ^]^ The bundle diameter *d*_Bundle_ is approximately related^[^ ^152^ ^]^ to the CNT diameter *d* and number of CNTs in the bundle *Z* by <*d*_Bundle_> ≈ <*d*>√*Z*. Bundles may consist of semiconducting and metallic CNTs, although their conductivity does not show a strong dependence on gate voltage when incorporated into a field effect transistor device.^[^ ^155^ ^]^ While a subject of debate, this lack of gate voltage may indicate that the metallic CNTs are primarily responsible for the transport within bundles.^[^ ^155^ ^]^ Alternatively, interactions between SWCNTs within a bundle modify the bundle conductivity itself and it is possible the typical notions of metallic and semiconducting CNTs lose their meaning when bundled. For a bundle of metallic armchair SWCNTs for example, adjacent SWCNTs destroy the rotational symmetry of the individual armchair SWCNT and this introduces a small pseudogap, or a partial reduction in the density of states at the Fermi level. This small pseudogap is typically 0.08 to 0.1 eV and is inversely dependent on CNT diameter.^[^ ^99^ ^]^ In another study, the distinct metallic and semiconducting characteristics of a metallic and semiconducting SWCNT were diminished as they were brought together into a bundle of two SWCNTs.^[^ ^156^ ^]^ In another related study, bandgaps closed for semiconducting SWCNTs and opened for metallic SWCNTs as they were brought together in bundles of two or three.^[^ ^157^ ^]^ For this reason, it is possible that the distinct metallic and semiconducting electronic character is washed‐out in large bundles. Further, it has been experimentally shown that the current density distribution over the cross‐section is not uniform, with only the outer CNTs participating in the bundle transport.^[^ ^158^, ^159^, ^160^ ^]^

Some reported individual bundles conductivities range between ≈1 and to 3 MS m^−1^ for bundle diameters from 20 to 5 nm.^[^ ^154^ ^]^ In another study, a 2.3 nm bundle yielded 3 MS m^−1^.^[^ ^158^ ^]^ These bundle conductivities are comparable to single‐crystal graphite (2.5 to 2.6 MS m^−1^)^[^ ^161^, ^162^ ^]^ and are notably well below measured values for individual metallic SWCNTs (67 to 200 MS m^−1^). The SWCNTs within a bundle are well‐aligned, optimally packed together, and the bundle has a mostly metallic resistance response with temperature.^[^ ^154^ ^]^ Therefore, this reported as‐is bundle conductivity, which is one to two orders of magnitude less conductive than an individual metallic SWCNT, is near an intrinsic conductivity limit in the undoped state. Unless bundle interaction can be mitigated, this means that 1 to 3 MS m^−1^ could be a more conservative upper bound conductivity of a completely undoped macroscopic CNT fiber, similar to single‐crystal graphite. Later with literature‐compiled datasets, we will show that the maximum conductivity of all undoped graphitic carbon materials, beyond intrinsic molecules, has a conductivity approximately that of single‐crystal graphite. This also includes the most conductive SWCNT fibers after de‐doping with a vacuum bake‐out process.^[^ ^15^ ^]^

As an analogy, graphene, like a metallic SWCNT, is a ballistic conductor and in the best possible case of zero defects, has an unavoidable phonon‐limited mean‐free‐path of ≈1 µm at room‐temperature.^[^ ^122^ ^]^ When graphene is perfectly assembled with ABAB stacking between layers, single‐crystal graphite is obtained with conductivities asymptotically approaching a limit near 2.5 to 2.6 MS m^−1^.^[^ ^161^, ^162^ ^]^ Here, increasing the area of the graphene planes of the single‐crystal graphite, when already substantially beyond the phonon‐limited mean‐free‐path, does not increase the intrinsic conductivity of the single‐crystal graphite.^[^ ^49^ ^]^ Similarly, increasing the length of CNTs, when already significantly beyond the mean‐free‐path, should not influence the intrinsic conductivity of the CNT bundle. Instead, as we will discuss later, increasing CNT length decreases the influence of extrinsic factors of the overall material, such as junctions between CNTs, impurities, unaligned regions, and voids. Larger crystallite domains in graphite and carbon fiber also increased the speed and degree of doping intercalation.^[^ ^49^ ^]^ In this way, CNT cables are likely to have an undoped, intrinsic conductivity dictated by CNT bundles and single‐crystal graphite, and this will be further supported in the discussions to follow. This notion underscores the importance of doping and understanding how graphitic intercalation compounds have already led to conductivities approaching the best metals. While metallic CNTs and graphene each individually have similar room‐temperature mean‐free‐paths, it is possible that an ordered, aligned, and doped CNT fiber may be superior to graphitic intercalation compounds in that: 1) aggregates of CNTs more easily accommodate bending; 2) the diameter of individual CNTs is an extra degree of freedom unavailable to the graphitic intercalation compounds. This enables, for example, higher overall material densities and better response to doping (particularly semiconducting CNTs) with closer, diameter‐dependent van Hove singularities compared to graphene. These enumerated hypotheses are supported in the previous discussion and the discussion to follow.

### 2.5 CNT Junctions

Junctions between CNTs and CNT bundles constitute a significant fraction of the resistance in a CNT network. One experimental study crossed pairs of various individual SWCNTs and measured the conductivity between them.^[^ ^163^ ^]^ The resistance across two crossed metallic SWCNTs ranged from 100 to 300 kΩ and between two crossed semiconducting SWCNTs ranged from 430 to 2600 kΩ. For comparison, remember that the ballistic resistance of a metallic SWCNT is 6.5 kΩ. The junction resistance between an activated semiconducting SWCNT to a metallic SWCNT was substantially worse because of the Schottky barrier interface. For this reason, transport in a SWCNT network is segregated between CNTs of similar electronic species. In terms of angle‐dependence between junctions, an atomic force microscope (AFM) study^[^ ^164^ ^]^ measured the contact resistance between individual MWCNTs (ranging from 9 to 46 nm in diameter) and graphite. As the individual MWCNT orientation was rotated across the graphite surface, they found a periodic fluctuation of the resistance with resistance minima occurring in increments of 60°; these resistance minima correspond to when the graphitic planes come into registry consistent with the expected six‐fold symmetry of the graphene lattice. A later AFM study^[^ ^165^ ^]^ showed a similar in‐registry periodic fluctuation when measuring the contact resistance between two large‐diameter (200 nm) MWCNTs. Here, complications from choosing different helicities and diameters for two crossed CNTs were eliminated by cutting one MWCNT and forming the junction between the two similar pieces. Also in this study, junctions between two MWCNTs with identical helicity and diameter were measured when they were placed in a parallel, overlapping configuration and were slid against each other. As the overlapping region between MWCNTs decreased with sliding, the average junction resistance generally increased. However, superimposed on this general resistance increase was also a periodically varying resistance component associated with the repeat length of the CNT unit cell.

In terms of contact resistance between bundles, one study^[^ ^158^ ^]^ found that smaller diameter bundles have junctions with higher conductivity. Across small CNT bundles 1.2 to 1.8 nm in diameter, the junction resistance averaged to 98 kΩ. Across bundles 4.5 to 6.8 nm in diameter, the bundle junction resistance averaged to 294 kΩ. Across large bundles 7 to 14 nm in diameter, the resistance was 2677 kΩ. A later experimental study however showed the opposite, with greater conductivity across larger bundles^[^ ^166^, ^167^ ^]^ and highlights the complexity of describing transmission across bundles.

## 3 Extrinsic Characteristics

With a better understanding how CNTs compete with other carbon conductors at the intrinsic level, we now examine extrinsic properties of various bulk CNT cables by considering an amalgamation of datasets across the literature.

### 3.1 Bulk Electrical Conductivity, Strength, and Multi‐Functionality

**Figure 3:**
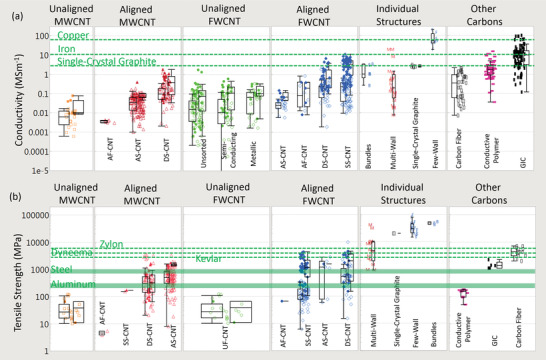
a,b) From data surveyed across the experimental literature: a) electrical conductivities and b) tensile strengths of CNTs, other carbon‐based conductors, and benchmark materials. Filled‐in shapes denote doped materials, as well as the right‐most box plot in each subcategory. Unfilled shapes and the left‐most box plots in each subcategory represent undoped materials. Green lines indicate benchmarks. Key: 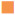) unaligned MWCNT materials; 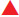) aligned MWCNT materials; 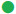) unaligned FWCNT materials; 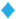) aligned FWCNT materials; 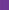) conductive polymers; 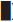) graphitic intercalation compounds; 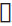) carbon fiber and graphite. M, F, B indicate individual MWCNTs, FWCNTs, and CNT bundles respectively. For the production method subcategories: AS‐CNT (derived from CNT forest arrays); UF/AF‐CNT (unaligned/aligned CNT film by filtering CNTs suspended in a fluid); DS‐CNT (aligned CNT materials directly extracted from FC‐CVD reactors); SS‐CNT (aligned CNT materials by extruding CNT solutions or suspensions into a coagulant).

The electrical conductivities of various CNT materials surveyed across the literature vary over five orders of magnitude (**Figure** **3**a). We first classify bulk CNT material using microstructure with either 1) a random in‐plane orientation or 2) some degree of CNT alignment. Figure 3a shows that aligned CNT materials (measured parallel with the microstructure alignment direction) are conclusively more conductive than unaligned. Figure 3a also shows CNTs categorized by broad structural type, either: 1) multi‐wall CNT (MWCNT) or 2) few‐wall CNTs (FWCNTs). FWCNTs have fewer nested CNTs than MWCNTs (the precise boundary is subjective in the literature), although now their smaller diameters enable more exotic optical and electronic properties such as van Hove singularities, stable 1D transport, radial breathing modes, and a bandgap that is strongly dependent on CNT structure. DWCNTs and SWCNTs make up most of this FWCNT category. Figure 3a shows that materials derived from FWCNTs are conclusively more conductive than those derived from MWCNTs, for both microstructure alignment categories as well as intrinsically for individual structures. The filled points in Figure 3a show the effect of doping, where the introduction of chemical species (either intentionally or as a side effect of other post‐processing) alters the conductivity (also represented by the right‐most box plot within each sub‐category). This can be accomplished intentionally by exposure to a specified chemical species, such as iodine vapor. Doping can also be a side‐effect of other post‐processes, such as with acid doping from acid‐based fiber production processes. This can be the case even after coagulant baths such as water or acetone have stabilized their conductivity, as residual acid constituents still remain. Unless extra measures, such as a vacuum bake, are used to remove these residual acid constituents (which reduces the original doped conductivity by a factor of approximately three to five^[^ ^15^, ^168^, ^169^ ^]^), in this meta‐analysis acid‐processed materials are considered doped.^[^ ^170^ ^]^ Unfilled points on Figure 3a are considered undoped, although there is likely still some natural p‐doping from exposure to outside air^[^ ^171^, ^172^, ^173^ ^]^ (also represented by the left‐most box plot within each sub‐category). Figure 3a shows that doping leads to the highest conductivity in all categories and is, so far, required to exceed the conductivity of single‐crystal graphite (2.5 to 2.6 MS m^−1[^ ^161^, ^162^ ^]^). There is at least one possible exception,^[^ ^168^ ^]^ which reported 4.0 MS m^−1^ for a fiber that was partially de‐doped during current‐carrying capacity tests, although this was not a traditional vacuum oven de‐doping procedure and it is likely to have retained some level of dopant. Only for a few cases reported so far have undoped CNT materials exceeded even 1 MS m^−1^.^[^ ^25^, ^26^, ^27^, ^168^, ^174^, ^175^, ^176^ ^]^

The green lines in Figure 3a represent standard benchmark materials, and in the last column, there is a category representing conductivity of other carbons (carbon fiber, conductive polymers, and graphitic intercalation compounds). Unaligned MWCNT material is the lowest CNT conductivity classification and has conductivity equivalent to or below that of glassy carbon (0.01 to 0.1 MS m^−1[^ ^177^ ^]^), an inert electrode material composed of disordered sp^2^ carbon bonds. Aligned MWCNTs and unaligned FWCNTs similarly have higher conductivities, although in an undoped state largely do not yet exceed the best commercial pitch‐based carbon fiber (0.75–0.9 MS m^−1[^ ^26^, ^78^, ^178^ ^]^). In the doped state, aligned MWCNTs^[^ ^134^ ^]^ and unaligned FWCNTs^[^ ^150^, ^179^ ^]^ approaches 1.8 MS m^−1^ and this surpasses the best laboratory graphitized carbon fibers (1.25 MS m^−1[^ ^18^ ^]^). The highest CNT category is doped, aligned FWCNTs. Within this category, fiber production via wet extrusion using super‐acid has yielded the highest conductivity with many reports residing between 2.5 and 8.5 MS m^−1,[^ ^15^, ^168^, ^170^, ^180^, ^181^ ^]^ with the highest value at 10.9 MS m^−1^.^[^ ^29^ ^]^ Wet extrusion takes CNTs from some growth source and mixes them in a liquid forming a suspension or solution where, after extrusion through an aperture, a fiber is made. Super‐acids enable a true CNT solution where CNTs individually protonate, de‐bundle, and align. For the purposes of standardization, we use the nomenclature proposed by Taylor et al.^[^ ^29^ ^]^ where these CNT materials are called SS‐CNTs for solution‐spun. Another prevalent production method within the aligned FWCNT category is direct spinning with floating catalyst chemical vapor deposition (FC‐CVD); here precursors in the gas phase are introduced into the hot zone of a tube furnace. Subsequent CNT growth into an aerogel cloud followed by its direct extraction out of the reactor yields a continuous CNT fiber with some degree of microstructure alignment, all in one production step. Again using the nomenclature proposed by,^[^ ^29^ ^]^ CNT materials made with this production process are called DS‐CNTs for direct‐spun. The highest doped conductivities for DS‐CNTs cluster between 1.2 and 4.3 MS m^−1[^ ^16^, ^27^, ^182^, ^183^, ^184^ ^]^ (when FWCNTs, not MWCNTs, are considered). There are two DS‐CNT papers^[^ ^14^, ^183^ ^]^ with higher conductivities up to 6.6 MS m^−1^; their fiber diameters were between 10 and 90 µm and were all heavily post‐processed with strong acids; in,^[^ ^14^ ^]^ there was additional iodine doping with a large scattering of conductivity values dependent on fiber diameter. Note that, instead of directly extracting CNT material from the FC‐CVD furnace, FC‐CVD‐derived CNTs can also be used in other production processes such as the initial stock for SS‐CNT materials. For example, the highest‐conductivity SS‐CNT fibers previously mentioned were made from CNTs produced by FC‐CVD reactors.^[^ ^29^ ^]^ Another subcategory of aligned FWCNT materials is bucky papers (or films made by filtering CNTs in a suspension with a membrane) with additional processing considerations for microstructure alignment. While there are several techniques available for this,^[^ ^185^, ^186^, ^187^ ^]^ a recently emerging method carefully controls filtering processes to create bucky paper films of high packing density and microstructure alignment.^[^ ^9^, ^188^, ^189^, ^190^ ^]^ Further, using sorted CNT feedstock, aligned bucky papers of full metallic or semiconducting SWCNTs has been demonstrated. Remarkably, the conductivity of these aligned bucky papers has so far remained low (<0.4 MS m^−1^), stemming from the fact they contain CNTs with short length, which will be discussed later. In line with the nomenclature of Taylor et al.,^[^ ^29^ ^]^ we call these materials AF‐CNT for aligned filtered; and UF‐CNT for unaligned filtered (traditional bucky paper). CNT materials derived from aligned CNT forests grown from catalyst patterned arrays, either spun into an aligned yarn or knocked down in an aligned film, are called AS‐CNT^[^ ^29^ ^]^ for array‐spun. Note that these subcategories specify methods of CNT production and can generally apply for both MWCNTs and FWCNTs. Overall, CNT materials have yet to reach the best conductive polymers (iodine‐doped polyacetylene at 15 MS m^−1^,^[^ ^30^, ^31^ ^]^ though unstable in air), standard metals (100% IACS copper at 58 MS m^−1^), or graphitic intercalation compounds (20 to 90 MS m^−1[^ ^32^, ^33^, ^35^, ^36^, ^37^, ^41^, ^43^, ^44^ ^]^ for those not stable in air and up to 10 MS m^−1^ for those stable in air^[^ ^32^, ^40^, ^45^, ^49^, ^50^, ^51^, ^52^, ^53^ ^]^). Graphitic intercalation compounds, so far, are the best room‐temperature Ohmic conductors in bulk morphology; they demonstrate that a conductivity exceeding that of copper is possible and moreover is accomplished with graphitic material. It is important to understand that the highest conductivity graphitic intercalation compounds (20 to 90 MS m^−1[^ ^32^, ^33^, ^35^, ^36^, ^37^, ^41^, ^43^, ^44^, ^47^ ^]^), while obtained by multiple different research groups, were nevertheless difficult to reproduce, short and discontinuous, mechanically impractical, and relatively unstable. CNT conductors can now be consistently manufactured in industrially relevant continuous lengths above the 10 MS m^−1^ benchmark conductivity with a strength approximating that of carbon fiber.^[^ ^29^ ^]^ In terms of practicality as a multifunctional mechanical or current‐carrying material, CNT materials, as they currently stand now, are superior to both graphitic intercalation compounds and conductive polymers. However, in order to understand how CNT materials can be improved further, it is important to discuss the conditions under which graphitic intercalation compounds and conductive polymers had higher maximum conductivities.

Figure 3a also shows the category of individual CNT structures, used as an estimated upper bound for the bulk conductivities. As discussed, individual SWCNTs can have an experimentally measured conductivity of 200 MS m^−1[^ ^71^ ^]^ if their diameter is used to calculate their cross‐sectional area, well above copper and graphitic intercalation compounds. However, the ordered agglomeration of FWCNTs into larger bundles leads to substantially lower conductivities (1 to 3.3 MS m^−1[^ ^158^, ^191^ ^]^). This bundle conductivity is similar to the best de‐doped aligned FWCNT fibers and to single‐crystal graphite (2.5 to 2.6 MS m^−1^), which is the most‐dense, ordered agglomeration of graphene. These similarities suggest that undoped CNT fibers (an agglomeration of CNTs) may not substantially exceed the conductivity of single‐crystal graphite (the densest agglomeration of graphene). Individual MWCNT conductivities span two orders of magnitude. They can exceed the measured conductivity of FWCNT bundles, although do not approach the conductivity of individual SWCNTs.

Strength: CNT materials are closer to competing with real‐world materials on the basis of mechanical strength than electrical conductivity. Figure 3b shows the tensile strength of CNT categories and benchmark materials. Unaligned MWCNTs and FWCNTs have lower strengths than their aligned counterparts (measured parallel to alignment direction) and are comparable to paper (3 to 100 MPa). Both aligned MWCNT and aligned FWCNT materials have larger strengths, with maximum clustered values reaching 4500 MPa. This is better than Kevlar (2800 MPa) and Dyneema (3900 MPa). It is also substantially greater than traditional conductive and high‐strength alloys (Copper 210 MPa, aluminum 241 MPa, steel 690 MPa, titanium 827 MPa) and approaches the best synthetic fibers, carbon fiber (2300 to 7100 MPa) and Zylon (5800 MPa). An application of t‐test shows that, while not the striking difference found with electrical conductivity, aligned FWCNT materials are on average stronger than aligned MWCNT materials (Figure S1, Supporting Information) and have higher maximum values. This includes the subset of aligned MWCNT fibers from forests (AS‐CNT) with stratified strengths^[^ ^24^, ^192^, ^193^, ^194^, ^195^ ^]^ (max 3200 MPa) approaching aligned FWCNT materials. Within the aligned FWCNT category, a t‐test shows directly extracted FC‐CVD fibers (DS‐CNT) and wet‐spun CNTs (SS‐CNT) have on average equal strengths, when considering the subset of wet‐spun fibers (SS‐CNT) only from super‐acid (Figure S2, Supporting Information). These t‐tests only illustrate differences in average material values, which may be problematic in that material categories are still developing. The maximum strength values between DS‐CNT^[^ ^25^, ^26^, ^27^, ^196^ ^]^ and SS‐CNT^[^ ^29^, ^67^, ^181^ ^]^ are also approximately similar and at this stage of development there is not a decisive lead between either process in terms of strength; there is one breakaway case of DS‐CNTs reaching a reported 9600 MPa.^[^ ^196^ ^]^ An application of t‐test shows no difference in strength between doped and undoped aligned MWCNT materials. For aligned FWCNT, from both DS‐CNTs and SS‐CNTs, doped materials are considerably stronger than undoped materials (Figures S3–S5, Supporting Information). This contrasts with conductive polymers, such as polyacetylene, which before doping have tensile strengths comparable to those of metals (300 to 900 MPa^[^ ^82^, ^197^, ^198^ ^]^). After doping, however, polyacetylene's tensile strength decreases by a factor of four to five due to reduced interfibrillar interaction (90 to 180 MPa).^[^ ^197^ ^]^ The highest conductivity graphitic intercalation compounds that reached copper's conductivity were formed from graphitized carbon fiber. The graphitization process (heat treatment in inert atmosphere at 2500 to 3000 °C) increases the structural perfection, Young's modulus, and conductivity of the carbon fiber host, at the cost of strength leading to brittle behavior and weakness and kinking in bending.^[^ ^18^, ^78^, ^199^ ^]^ While not obtaining copper's conductivity, less conductive graphitic intercalation compounds (4 to 10 MS m^−1[^ ^18^, ^55^, ^56^ ^]^ and, at least in one case, 16 MS m^−1[^ ^45^ ^]^) have post‐doped strengths that compare to their host carbon fiber (1090 to 3000 MPa). Here, the doping process reduced mechanical strength by approximately 25–50%^[^ ^18^, ^45^, ^56^ ^]^ and was dependent on the intercalation procedure and host carbon fiber. The comparable strength and superior conductivity of this subset of graphitic intercalation compounds make them a somewhat less fashionable rival to current CNT materials.

Shear strength between CNTs, as opposed to the much higher intrinsic tensile strength of CNTs, is the typical limiting factor in the strength of most CNT materials with an aligned microstructure; the bulk modulus is dictated by the modulus of the individual CNTs.^[^ ^170^, ^200^, ^201^, ^202^, ^203^, ^204^ ^]^ Graphite again is an analogous material where the individual graphene sheets are much stronger in‐plane than the van der Waals interactions which bind the graphene sheets together. Here, the shear force from the van der Waals interactions, unlike a typical macroscopic frictional force, has a strength proportional to the overlap area and is not significantly dependent on the normal force.^[^ ^205^ ^]^ Molecular dynamics simulations^[^ ^206^, ^207^ ^]^ have shown that material strength increases with CNT length and degree of cross‐linking between CNTs. For higher densities of cross‐linking, the reliance on CNT length was less critical. The maximum tensile strength of a optimally cross‐linked CNT fiber was calculated to be 60 GPa.^[^ ^206^ ^]^ Collapsed CNTs into a “dog‐bone” structure have the advantage of greater innertube friction and higher density; tight‐binding atomistic calculations have demonstrated a Young's modulus of 1100 GPa.^[^ ^208^ ^]^ Amorphous carbon,^[^ ^205^ ^]^ oligomeric by‐products from CVD reactors,^[^ ^209^ ^]^ and post‐process cross‐linking^[^ ^175^, ^195^, ^210^, ^211^, ^212^ ^]^ tie individual CNTs together, increasing their overall strength. Finite element analysis^[^ ^205^ ^]^ demonstrated that, for perfectly packed, aligned, and impurity free CNT fibers under load, the stress distribution varies substantially in the radial direction, being stronger on the surface and tapering off in the fiber core. It was proposed that some partial misalignment and impurities assisted uniform stress transfer across the cross‐section and increased the tensile strength of practical CNT fibers over realistic gauge lengths. In situ Raman measurements demonstrated that, at least in certain CNT fibers under load, the stress distribution is not necessarily homogeneous over the fiber gauge length and uneven stress transfer across bundles limits the overall fiber strength.^[^ ^213^ ^]^

To estimate an upper bound on the bulk strength, we now discuss the strengths of experimentally measured individual CNTs. Individual FWCNT bundles have strengths measured from 43 000 to 48 000 MPa^[^ ^201^ ^]^ using very long gauge lengths and the entire CNT diameter (rather than an annular region) to calculate the effective cross‐section. Here, the gauge length is comparable to the CNT bundle length and in this way is an intrinsic strength measurement. This strength is over twice the value of single‐crystal graphite (20 000 MPa^[^ ^18^ ^]^) and allows for significantly more flexibility in the bulk material. It was shown that, without any preconditioning treatment, the tensile strength decreased exponentially as more CNTs were incorporated into a bundle; this is due to the Daniels effect^[^ ^201^ ^]^ where CNTs break sequentially as load is applied, as opposed to the load being uniformly distributed over all the CNTs in a bundle. Through post‐treatment tightening and relaxation steps, the internal CNTs can be aligned on the nanoscale, and the bundles could be made much stronger because they now have a more uniform stress distribution.^[^ ^201^ ^]^ The strength of individual CNTs can be higher than bundles with individual FWCNTs measured between 25 000 and 96 000 MPa^[^ ^201^, ^214^, ^215^ ^]^ and individual MWCNTs measured between 10 000 and 42 000 MPa.^[^ ^216^, ^217^ ^]^ Note that for the individual strengths we are discussing, the engineering values that use the full cross‐sectional area, rather than the thin annular region around the CNT's diameter. Helicity has also been shown to affect tensile strength, where small diameter near‐armchair helicities yield the strongest CNT structures.^[^ ^214^ ^]^

**Figure 4:**
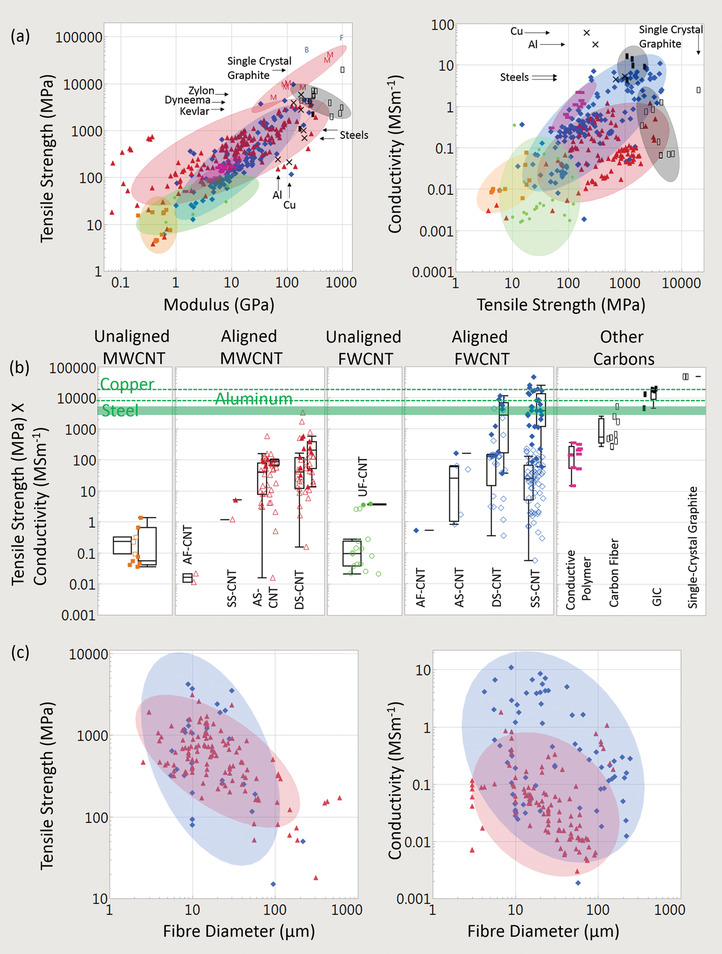
Conductivity and strength relationships in CNT materials and benchmarks across the literature surveyed. a) Tensile strength versus Young's modulus and conductivity versus tensile strength. b) Depiction of the multifunctional metric (conductivity multiplied by tensile strength), partitioned by CNT categories. c) Dependence of conductivity and strength on fiber diameter. Key: 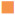) unaligned MWCNT material; 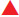) aligned MWCNT materials; 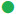) unaligned FWCNT materials; 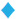) aligned FWCNT materials; 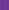) conductive polymers; 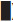)graphitic intercalation compounds; 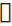) carbon fiber and graphite. M, F, B indicate individual MWCNTs, FWCNTs, and CNT bundles respectively. “*x*” indicated annotated benchmarks. Only in (b) do filled in shapes indicate doped materials. Ellipses help identify trends and are adjusted to cover 90% of the points. For the production method subcategories: AS‐CNT (derived from CNT forest arrays); UF/AF‐CNT (unaligned/aligned CNT film by filtering CNTs suspended in a fluid); DS‐CNT (aligned CNT materials directly extracted from FC‐CVD reactors); SS‐CNT (aligned CNT materials by extruding CNT solutions or suspensions into a coagulant).

**Figure** **4**a shows a plot of tensile strength versus Young's modulus (with double logarithmic axes) of the CNT categories, correlation data are given in **Table** **1**. This table (and the ones that follow) includes the correlation coefficient, the power‐law exponent and error, and the *p*‐values. This also includes an adjustment of these values where each paper is given equal “weight” independent of the number of data points they offer. While unaligned CNT material shows no correlation, the aligned MWCNT and FWCNT materials nearly overlap, with correlation persisting even after the weighted adjustment accounting for number of data points. The positive correlation between tensile strength and Young's modulus is typical of the behavior found in conductive polymers and synthetic fibers, where the implicit parameter benefiting both tensile strength and modulus is microstructure alignment.^[^ ^15^, ^82^ ^]^ Carbon fiber too can have a similar positive relationship between tensile strength and Young's modulus. After graphitization annealing, however, the correlation for carbon fiber becomes negative (Figure 4a).^[^ ^18^, ^78^, ^199^ ^]^ While aligned CNT materials have approached the tensile strength of the best carbon fiber, as shown in Figure 4a, carbon fiber is still superior in terms of modulus. We note that CNT‐based materials have much greater flexibility and bending radius over traditional carbon fiber; CNT fibers do not lose much if any strength when knotted, unlike knotted carbon fiber that experiences severe weakening.^[^ ^1^ ^]^

**Table 1:**
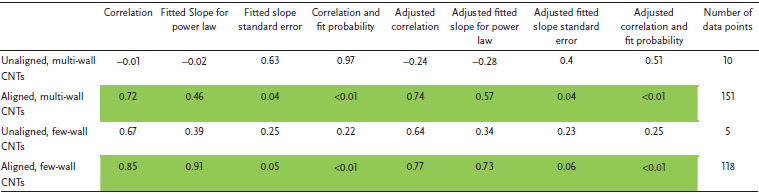
Log tensile strength versus log Young's modulus

Figure 4a also plots electrical conductivity against tensile strength, with correlation data provided in **Table** **2**. For the aligned CNT categories, a positive correlation is present and persists without much change after the weighted adjustment. This notion of greater strength with higher conductivity has been captured in multiple individual studies.^[^ ^170^, ^218^, ^219^ ^]^ Conductive polymers behave similarly, where microstructure orientation again is the implicit parameter simultaneously affecting both strength and conductivity.^[^ ^82^, ^197^ ^]^ In terms of a power‐law, for reasons we will explain later, both the conductivity and strength of aligned CNT materials depend similarly on microstructure alignment and CNT length. For these reasons we expect a power‐law exponent of unity between conductivity and strength for the aligned CNT material. Indeed, for the aligned FWCNT the fitted slope is approximately unity; for aligned MWCNTs the value is unexpectedly lower. Carbon fiber can have a negative correlation between conductivity and strength, where the strongest carbon fibers are disordered; a defective microstructure crosslinks crystalline regions and this comes at the cost of conductivity.^[^ ^18^, ^60^, ^78^ ^]^

**Table 2:**
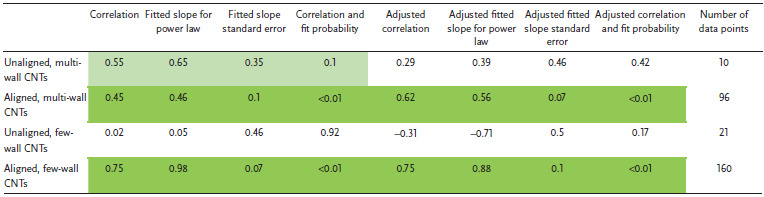
Log conductivity versus log strength

We showed that aligned CNT materials, as well as some graphitic intercalation compounds, have strengths that approach the strongest carbon fiber and synthetic fibers, while having substantially higher conductivities approaching copper; combining these attributes, we now introduce a multifunctional metric—the product of conductivity and tensile strength (Figure 4b). This metric is useful for easily identifying materials with the highest strength and conductivity concurrently. As a figure of merit for determining the best material for applications where strength and conductivity is critical (say, suspended cables for electrical power utility lines), it is not necessarily the case that a simple product is the most appropriate metric, and the optimal functional form would depend on the details of the implementation of the application. Doped, aligned FWCNT materials are clearly the highest ranking CNT category with acid‐extruded fibers (SS‐CNT) the highest ranking subset. CNT materials in this respect rank higher than all existing benchmark materials: metal, carbon fiber, and graphitic intercalation compounds. Although not shown on this graph, we also note that CNTs incorporated into metals as a composite also can yield similarly high multifunctional metric values;^[^ ^220^, ^221^, ^222^ ^]^ in any case CNTs yield the best multifunctional metric. Single crystal graphite is still even greater, although these are small brittle flakes impractical for use outside a laboratory.^[^ ^161^, ^162^, ^223^ ^]^

It is also known that smaller diameter fibers for CNTs^[^ ^11^, ^14^, ^224^, ^225^, ^226^ ^]^ and carbon fiber^[^ ^45^, ^227^, ^228^ ^]^ tend to have fewer voids, better microstructure alignment, and greater order over their cross‐section; this results in higher conductivity and tensile strength for smaller diameter fibers. There are however some counterexamples to this trend^[^ ^229^ ^]^ and it does not seem to hold true for graphitic intercalation compounds.^[^ ^40^, ^51^, ^53^, ^230^ ^]^ In general as the scale of the CNT assembly grows from an individual CNT, to a bundle, to a fiber, to a rope or cable or textile, the conductivity and tensile strength decreases due to a greater influence of extrinsic factors such as voids, impurities, and unaligned regions.^[^ ^60^ ^]^ Figure 4c and correlation **Tables** **3** and **4** shows the relationship of conductivity and strength against fiber diameter across the literature surveyed. For tensile strength and conductivity, there is the expected negative correlation with fiber diameter for both aligned FWCNT and aligned MWCNT material, before and mostly after the weighted adjustment. Note that, after the weighted adjustment, the conductivity to fiber diameter relationship for the aligned MWCNT category flips sign unexpectedly, although this is on the edge of even weak statistical significance. This is likely a spurious correlation in consideration of the large statistical significance of the other correlated categories. Also note that for the conductivity and strength correlations with fiber diameter, we found one data point,^[^ ^231^ ^]^ which was a significant outlier with low physical properties and which, when included, clearly skewed the correlations. These fibers were substantially smaller (1 µm diameter and smaller) than the other bulk, macroscopic fibers we compare to and were made with a dielectrophoretic technique that was otherwise not used elsewhere in this meta‐analysis. This data point was excluded from the correlation and power‐law calculation.

**Table 3:**
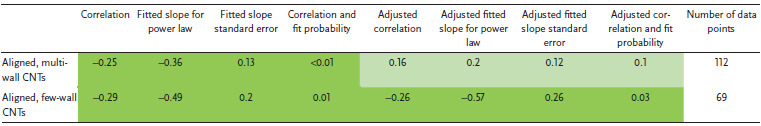
Log conductivity versus log fiber diameter

**Table 4:**
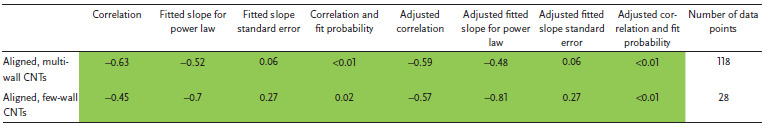
Log tensile strength versus log fiber diameter

### 3.2 Thermal Conductivity and Ampacity

**Figure 5:**
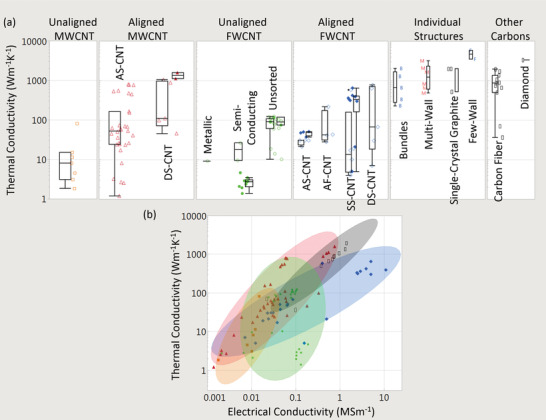
a) Thermal conductivity of CNT categories and benchmarks. b) Thermal conductivity versus electrical conductivity. Key: 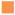) unaligned MWCNT material; 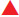) aligned MWCNT materials; 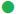) unaligned FWCNT materials; 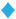) aligned FWCNT materials; 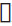) carbon fiber, diamond, and graphite. M, F, B indicate individual MWCNTs, FWCNTs, and CNT bundles respectively. Only in (a) do filled in shapes indicate doped materials, as does the right‐most box plots in each subcategory. Ellipses help identify trends and are adjusted to cover 90% of the points. For the production method subcategories: AS‐CNT (derived from CNT forest arrays); UF/AF‐CNT (unaligned/aligned CNT film by filtering CNTs suspended in a fluid); DS‐CNT (aligned CNT materials directly extracted from FC‐CVD reactors); SS‐CNT (aligned CNT materials by extruding CNT solutions or suspensions into a coagulant).

**Figure** **5**a shows the thermal conductivity of the various CNT categories across the literature, compared against the individual intrinsic structures and other carbons. As previously established with electrical conductivity and strength, unaligned CNT material is categorically less thermally conductive than the aligned; the intrinsic structures are substantially more thermally conductive than the bulk materials in descending order of FWCNTs, MWCNTs, and bundles. Aligned CNTs have yet to reach the thermal conductivity of diamond (3320 W m^−1^ K^−1[^ ^209^ ^]^) and single‐crystal graphite (2000 W m^−1^ K^−1[^ ^232^ ^]^). CNTs have just reached the level of laboratory‐scale graphitized carbon fiber (1000 to 2000 W m^−1^ K^−1[^ ^232^, ^233^, ^234^ ^]^). Industrial available quantities of carbon fiber^[^ ^235^, ^236^ ^]^ can reach 500 to 1060 W m^−1^ K^−1^ when graphitized. Contrary to this trend, the aligned MWCNT materials are on average just as thermally conductive as the aligned FWCNT material (Figure S6, Supporting Information) and also have the highest maximum values (exceeding 1000 W m^−1^ K^−^^1[^ ^237^ ^]^).

Thermal energy generally can be transmitted by phonons as well as electronic charge carriers, although for CNTs it has been thoroughly demonstrated that phonons are the dominant mechanism for thermal transport.^[^ ^209^, ^238^ ^]^ The mean‐free‐path length for room‐temperature phonons can be 500 nm for individual MWCNTs and longer for individual FWCNTs.^[^ ^209^, ^239^ ^]^ When brought together as a bundle, the thermal conductivity drops (Figure 5a) due to intertube interactions that strongly increase phonon scattering.^[^ ^240^ ^]^ Despite the decoupling between the electronic and thermal transport, in practice there is still a large correlation between thermal conductivity and electrical conductivity for the aligned CNT materials (Figure 5b and **Table** **5**). This correlation is not present for the unaligned CNT materials and supports the observation that microstructure alignment benefits transmission of both charge carriers and phonons, as opposed to some intrinsic connection between thermal conductivity and electrical conductivity (e.g., the Wiedemann–Franz law in metals).

**Table 5:**
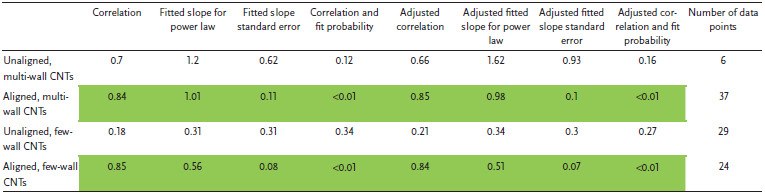
Log thermal conductivity versus log conductivity

Ampacity, or current‐carrying capacity, is an important metric for practically sizing electrical wires; however, it is not a fundamental material parameter, being a function of electrical conductivity, thermal conductivity, heat capacity, chemical stability, the effects of the testing environment, grain boundaries, melting or decomposition points, mechanical failure and most importantly, fiber diameter. Here, we review ampacity metrics reported across the CNT literature and uncover important trends with this meta‐analysis. Ampacity is not routinely measured in the CNT literature and in most cases, may warrant better testing protocols; hence, unlike electrical conductivity, the data are sparse, but nonetheless sufficient to make certain meaningful comparisons. First, we discuss various definitions of ampacity, followed by a comparison of ampacity values for various CNT types, and how these relate to the conductors’ diameters.

**Figure 6:**
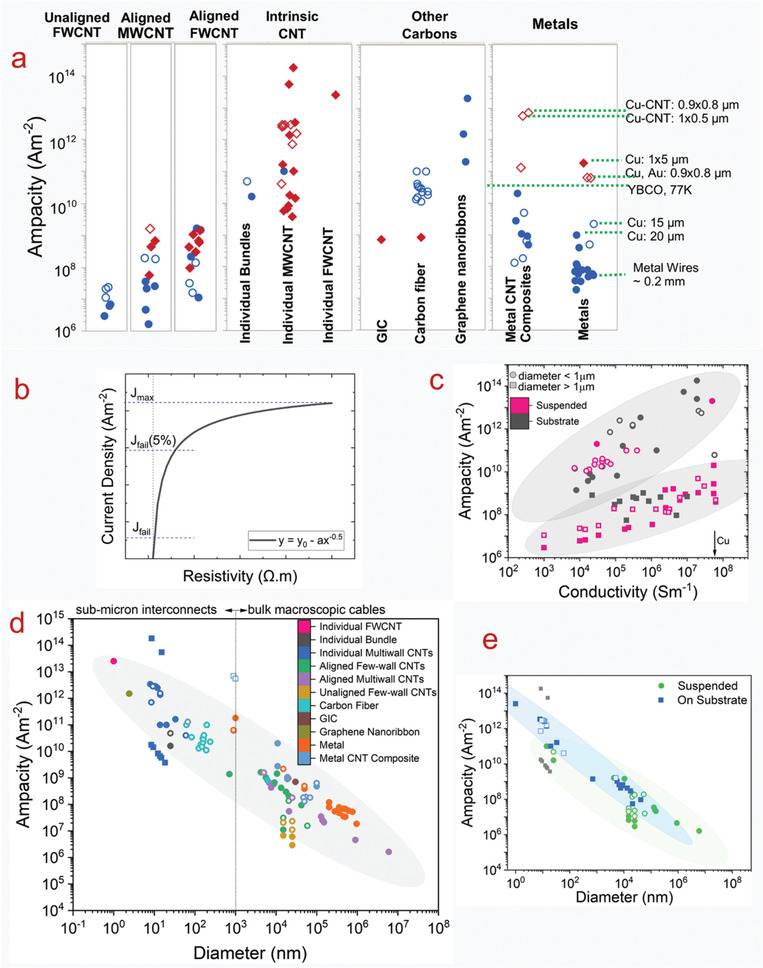
Ampacity relationships, open symbols indicate vacuum measurements and filled symbols indicate measurement in air, for all graphs. a) Ampacity of CNT materials surveyed across the literature, along with metals and other carbon‐based conductors. Blue circles are suspended samples, while red diamonds are samples measured on various substrates. b) An example of current density versus resistivity curve. c) Log−log plot of ampacity versus conductivity for all material classes. d) Ampacity versus diameter master curve for all materials studied here including metals, metalized CNTs, pure CNTs, and other carbons. Samples that did not fail or whose diameters cannot be confidently determined are shown with square symbols. e) Ampacity versus diameter plot for CNT materials only classified based on presence or absence of substrates. The ellipses in this figure (only) represent a 95% confidence region of the linear fits from which the power‐law exponents can be obtained.

The ampacity of current‐carrying conductors can most broadly be defined as the maximum current a conductor can sustain before damage. Since the damage to a conductor may occur in multifarious forms such as melting, fusing, or breaking, in practice, ampacity refers to an ensemble of metrics. For example, standard unclad electrical wires of copper and aluminum are rated in terms of the currents required to fuse or melt wires of a given diameter (*d*_Conductor_) or gauge. Typical fusing current densities for metals are in the range 10^7^ to 10^8^ A m^−2^.^[^ ^241^ ^]^ From a safety perspective, wires intended for regular use are more commonly rated an order of magnitude or two lower than the maximum current density at break (*J*_max_). These metrics, known either as the current carrying capacity (CCC) or continuous current rating (CCR, *J*_CCR_), represent the maximum current density before a certain temperature threshold is reached.^[^ ^168^, ^242^ ^]^ CCC is relevant for wires with an outer polymer cladding, which melt at far lower temperatures than the conductor; CCC values for commonly used metals are provided in standard tables such as IEC 60287. Failure, in the above cases, occurs due to Joule heating exceeding the rate at which heat can be dissipated from the surface of a wire, thus raising the temperature of the conductor beyond its melting or decomposition points. Since the amount of heat dissipated increases with the surface area, smaller diameter conductors can carry greater current densities (although, lower total currents) before they melt.^[^ ^168^, ^243^ ^]^ However, metallic filaments tend to become fragile below 15 to 20 µm, thereby limiting practically achievable ampacities from metal wires. The development of metal evaporation and coating processes solved this issue; thin, narrow lines of metals, called interconnects, could be “drawn” on various substrates which allowed for greater current densities, thus aiding miniaturization of electronics. In small dimensional conductors, however, another competing damage mechanism, electromigration, takes over. Electromigration is the diffusion of metal ions due to momentum transfer from charge carriers at high currents; it was confirmed by the movement of scratch marks on a gold wire subjected to high current densities.^[^ ^62^, ^242^, ^244^ ^]^ Electromigration results in the formation of voids and hillocks in conducting pathways, and can cause failure well before the conductors’ melting points (fusing currents) are reached.^[^ ^245^, ^246^ ^]^ These electromigration effects become significant in sub‐micrometer interconnects due to their narrow channel widths and thicknesses, increasing chances of failure.^[^ ^247^ ^]^ Cu interconnects with thickness (and widths) in the micrometer regime boast electromigration limited ampacities in the range 10^10^ to 10^11^ A m^−2^.^[^ ^248^ ^]^ Continuing miniaturization of electronics will, therefore, require mitigation of electromigration effects or development of new materials to push ampacities closer to the conductors’ fusing current densities. We have depicted the ampacities of some commonly used metal wires and interconnects in **Figure** **6**a.

Carbon‐based conductors are particularly suited for high current electronic applications as their strong C—C bonds resist electromigration, while the π orbitals allow for electronic conduction.^[^ ^242^, ^249^ ^]^ Electromigration‐like effects in CNT cables are limited to Fe catalyst impurities or embedded metals, which tend to sweat out or diffuse to the surface at high currents.^[^ ^62^, ^168^, ^242^, ^250^, ^251^ ^]^ Sometimes the residual catalyst sweating causes microtears, although it is unlikely these sweating effects cause breakage in CNT fibers. Thus, the ampacity‐related failure mechanism in pure CNT cables is Joule heating. Here, the most widely used ampacity metric is the maximum current density before fiber break (*J*_max_).^[^ ^141^, ^168^, ^252^ ^]^ While breaking is a perfect external indicator of damage, the exact value depends on current sweeping conditions; for example, current induced desorption of dopants or solvents may cause changes to material resistivity or the structure, altering the failure conditions.^[^ ^168^ ^]^ Wang et al.^[^ ^168^ ^]^ argue that a more meaningful definition of ampacity is the point at which the resistivity (ρ) of the fiber begins to change irreversibly (*J*_fail_), due to structural or chemical changes. As illustrated in the example *J*–ρ curve in Figure 6b, irreversible changes in resistivity are not a sharp transition; we suggest using a mathematically definable point, such as when resistivity increases by 5% (we call this *J*_fail_ (5%)). Unless specified that the ampacity in question is the irreversible resistivity point (*J*_fail_),^[^ ^168^ ^]^ for the purposes of this meta‐analysis, we have assumed that the reported ampacity values for bulk materials are all maximum current densities at break (*J*_max_).

Figure 6a depicts the ampacity (*J*_max_) values of carbon‐based conductors across our primary categories scavenged from 32 publications (22 on CNTs). Like all previous metrics, ampacity is highest for individual CNTs. Early experiments on isolated MWCNTs, surface‐contacted by a scanning tunneling microscope (STM) tip, showed that they could withstand current densities greater than 10^10^ A m^−2^.^[^ ^138^ ^]^ In another work, end‐contacted MWCNTs were shown to conduct electrons ballistically up to a length of 2 µm with current densities greater than 10^11^ A m^−2^.^[^ ^253^ ^]^ An interesting consequence of this ballistic transport is that the heat is dissipated at the contacts and not in the conductor, so the failure should occur at the contacts.^[^ ^253^, ^254^ ^]^ Later experiments on MWCNTs^[^ ^135^, ^141^, ^249^ ^]^ did not observe ballistic transport, but were able to demonstrate higher current densities due to the use of low contact resistance electrodes. Experiments by Collins et al.^[^ ^249^ ^]^ and Huang et al.^[^ ^135^ ^]^ in side‐contacted and end‐contacted geometry, respectively, showed that MWCNTs undergo a shell‐by‐shell breakdown at current densities of about 10^12^ Am^−2^, confirming current saturation. Wei et al.^[^ ^141^ ^]^ reported the highest ever MWCNT ampacities at 10^14^ Am^−2^ without observing any current saturation. It is not clear if they were able to achieve ballistic transport, as these values clearly stand out as outliers in the individual MWCNT category. In this case for SWCNTs,^[^ ^124^ ^]^ current saturation at high‐bias was inferred from the current‐voltage curves which gives an upper bound of 10^13^ A m^−2^ for their ampacities. As a summary comparison, the ampacities for individual CNTs are: SWCNTs, 10^13^ A m^−2^;^[^ ^124^ ^]^ MWCNTs, 10^11^ to 10^12^ A m^−2^;^[^ ^135^, ^249^, ^253^ ^]^ and bundles^[^ ^255^ ^]^ (25 nm diameter), greater than 10^10^ A m^−2^. Individual carbon nanofibers (not to be confused with carbon fiber) show ampacities in the range 10^10^ to 10^11^ A m^−2^,^[^ ^256^ ^]^ while graphene nanoribbons can carry 10^12^ to 10^13^ A m^−2^.^[^ ^257^ ^]^

The earliest bulk CNT material study^[^ ^255^ ^]^ involved bucky papers/films grown from HiPCO CNTs. These unaligned FWCNTs exhibit far lower breakdown current densities than those of individual nanotubes: about 3 to 7 × 10^6^ A m^−2^ in air and three to four times higher in vacuum; aligning the microstructure increased their ampacity a factor of 1.5 to 2 times. Fiber morphologies tend to be more aligned, showing higher ampacities. CNT fibers made from either purified or doped DWCNT cotton^[^ ^14^, ^252^ ^]^ (with short probe spacings) showed conductivities of 0.6 to 0.1 MS m^−1^ and ampacities of 10^9^ to 10^8^ A m^−2^ in vacuum, respectively. Using a finite element model for the fiber in a core‐shell geometry, they confirmed that the breaking is due to inhomogeneous Joule heating. CNT fibers extruded from acid solutions (SS‐CNTs) tend to be denser and heavily doped and, in one case, gave them a higher conductivity of 2.5 to 3 MS m^−1^.^[^ ^168^ ^]^ The ampacities of such fibers were found to be approximately 10^8^ A m^−2^, which is lower than that of copper, but on the basis of weight becomes comparable.^[^ ^168^ ^]^ Aligned MWCNT fibers which are acid‐treated can also have an ampacity of approximately 10^8^ A m^−2^, while pure untreated MWCNT fibers tend to exhibit lower values of approximately 10^7^ A m^−2^.^[^ ^258^ ^]^ In that study, improvement in ampacity was largely due to mechanical condensation rather than acid treatment. MWCNT fibers with large fiber diameters (100 µm to 6 mm) or poorer alignment show even lower ampacities of 10^6^ A m^−2^.^[^ ^259^, ^260^ ^]^ All together, these results suggest that improvements in density and alignment are more critical to ampacity than other factors such as doping.

Before analyzing the full collection of ampacity data in the meta‐analysis, we briefly discuss the Joule heating mechanisms to derive the expected power‐laws. Joule heating in a wire can be balanced with one or some combination of the following cooling pathways: 1) cooling through conduction in the fiber, end contacts or substrates, 2) convection by surrounding gases, and 3) blackbody radiation. The classical law describing ampacity follows from the works of both Forbes and Preece in the 1880s on conventional metal wires,^[^ ^243^ ^]^ where convection in a surrounding background gas is the primary cooling pathway. Known as the “Fuse law,” it states that the current required to melt a metallic wire is related to its diameter as *I*_max_ ∝ *d*_Conductor_^3/2[^ ^243^ ^]^ or *J*_max_ ∝ *d*_Conductor_^−1/2^. We can derive a more generalized relation by equating the Joule heat generated in a cylindrical wire, 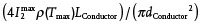, to the convective heat loss flow rate, $\dot{Q}\;\;\;=\;\;\;h_{\text{conv}}\;\pi d_{\text{Conductor}}\;\;L_{\text{Conductor}}(\Delta T)$ where *L*_Conductor_ is the length of the conductor, Δ*T* is the temperature difference between the sample (*T*_max_) and ambient (*T*_amb_), and *h*_conv_ is the convective heat transfer coefficient with units W m^−2^ K^−1^. This assumes heat loss is through convection over the surface of the wire and not conduction through the ends. The resulting power‐law is as follows

(2) $$J_{\max}\propto {\left(d_{\text{Conductor}}\right)}^{-1/2}{\left(\sigma \left(T_{\max}\right)\right)}^{1/2}{\left(\Delta T\right)}^{1/2}$$

By solving the 1D steady‐state heat equation, Suzuki et al.^[^ ^256^ ^]^ and Wang et al.^[^ ^168^ ^]^ arrived at another set of current density temperature relations. While both studies agree with *J*_max_ ∝ *T*_max_^1/2^σ(*T*_max_)^1/2^, they differ on the predicted diameter dependence. Suzuki et al.^[^ ^256^ ^]^ were concerned with short conductors (<250 nm) in which the current saturates at high bias, such as carbon nanofibers or CNTs. In such a case, where conduction through the ends is a significant heat sink, they found *I*_max_ is independent of diameter and *J*_max_ ∝ *d*_Conductor_^−2^. In addition, the heat equation solution develops a weak dependence on the length of conductors between contacts. Wang et al.'s theory,^[^ ^168^ ^]^ which is applied to larger fibers, agrees with the convection mechanism as given by Equation (2) with one important difference—they found using natural convection theory that the thermal conductance, *g* (dimensionally equivalent to the term *h*_conv_ used here), is dependent on gas composition and diameter as *g* ∝ *d*_Conductor_^−0.826^. Substituting the diameter dependence of thermal conductance into Equation (2), we get

(3) $$J_{\max}\propto {\left(d_{\text{Conductor}}\right)}^{-0.913}{\left(\sigma \left(T_{\max}\right)\right)}^{1/2}{\left(\Delta T\right)}^{1/2}$$

The above equation is valid for different gaseous media surrounding the fiber, but not for vacuum where *g* = 0. In the absence of convection, heat loss through black‐body radiation from the surface of the conductor may become dominant, in which case *J*_max_ ∝ *d*_Conductor _^−1/2^ *σ(* *T*_max_)^1/2^(*T*_max_^4^ − *T*_amb_^4^)^1/2^. At temperatures where most materials melt, losses by radiation are small compared to those by convection in a medium.^[^ ^168^ ^]^ So, in general, the ampacity in vacuum is expected to be smaller than the ampacity in air. Specific material property particularities with CNTs complicate this analysis. For example, heating in vacuum de‐dopes CNT fibers, further reducing their ampacity in vacuum. However, carbon‐based conductors burn in oxidizing environments (for CNTs in air, burning between 450 and 800 °C^[^ ^255^, ^261^ ^]^) and are structurally stable to much higher temperatures in vacuum. Depending on the CNT fiber, irreversible structural changes start in vacuum between 1400 and 1800 °C^[^ ^251^, ^255^, ^262^, ^263^, ^264^ ^]^ and carbon sublimes at greater than 3550 °C;^[^ ^251^ ^]^ therefore, the ampacity difference between air and vacuum is not simple to analyze universally. From specific studies in the literature, CNT bucky papers and CNT–copper composites showed slightly higher ampacities in vacuum,^[^ ^255^, ^265^ ^]^ while acid extruded FWCNT fibers (SS‐CNTs) showed two times lower ampacities in vacuum.^[^ ^168^ ^]^ In respect to the agglomeration of ampacities in the meta‐analysis, large‐scale samples (greater than 1 µm diameter) did not display a difference between air and vacuum (t‐test, not shown here). Summarizing the expected ampacity dependence power‐laws, we expect the exponent for conductivity to be 1/2 and for diameter, somewhere between −1/2 and −1 dependent on the exact heat‐sink pathway.

Equipped with the above understanding, we proceed with the meta‐analysis of ampacity relationships. Conductors that do not fail (except in case of saturation) or whose cross‐sectional areas are not confidently known are excluded from the correlation analysis, although are depicted in the plots. For materials with a rectangular cross‐section, we use an effective diameter which we define as the thickness of the strip (*H*_Conductor_); similar to Equation (2), when the width of a conducting strip is much larger than thickness, *J*_max_ ∝ *H*_Conductor_^−1/2^. Rather than partitioning the data between CNT types as accomplished in the rest of the meta‐analysis, just for ampacity, the data are better partitioned according to experimental conditions as uniquely specified in each plot. Also, just for this ampacity discussion, we have included other materials such as metals and CNT–metal composites to better highlight the relationships between conductivity and diameter. In Figure 6c, across all material and experimental categories, we plot ampacity versus electrical conductivity. Two populations become apparent, distinguished by fiber diameters greater than or less than 1 µm; note that most of the samples thinner than 1 µm have a probe separation less 1 mm. For the population with fiber diameter greater than 1 µm (correlates with probe separation larger than 1 mm), there is a significant correlation with a fitted power‐law exponent very close to the expected value of 1/2, both before and after the weighted adjustment (**Table** **6**), which is consistent with specific studies^[^ ^255^, ^256^ ^]^ and the scaling laws derived above. The second population, fibers less than 1 µm in diameter, are also highly correlated although the fitted power‐law exponent is somewhat higher at 0.88. Their small probe separation and fact that most are in contact with a substrate (black), opposed to suspended (pink), complicate current and heat‐flow.

**Table 6:**
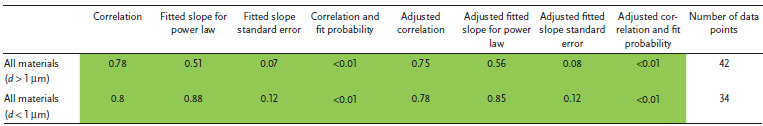
Log ampacity versus log conductivity

Figure 6d shows ampacity versus diameter (6 mm down to 1 nm) for all material categories and experimental configurations (air, vacuum, on‐substrate, and suspended); there is a strong negative correlation across a wide range of diameters and materials. Note that here we excluded the highly cited, high‐ampacity obtained^[^ ^242^ ^]^ from the correlation analysis because it is a visible outlier and substantially skews the relationship (although have included it on the plot). With the data otherwise taken in its entirety without partitioning, the power‐law exponent is very close to −1. When considering just the subset of air measured samples (solid symbols), this becomes −0.9, which is very close to the predicted value (**Table** **7**). Samples measured in vacuum have a power‐law exponent of almost −1; this should not necessarily be true for vacuum measurements where convection is not possible and where black‐body radiation dictates a power‐law exponent of −1/2. As explained above, for material property reasons specific to carbon materials, the distinction of ampacity measurement between vacuum and air is nuanced and is not immediately obvious in specific studies or this meta‐analysis that substantial differences exist. Now taking this dataset in its entirety, and simply partitioning between suspended and substrate‐bound subsets, we find a similar strong ampacity–diameter correlation with power‐law exponents of −0.9 and −1.1 (Table 7). The substrate‐bound power‐law exponent is slightly more negative than predicted, although this is not surprising considering the more complicated heat‐flow situation. Restricting the dataset to CNT materials (Figure 6e, excluding all other materials as well as CNT composites), we obtain similar power‐law exponents with the suspended and substrate‐bound partitioning, both before and after the weighted adjustment. We have also indicated on top of Figure 6d, two regimes: the sub‐micron interconnect regime and the macroscopic cable regime for conductors smaller and greater than 1 µm in diameter, respectively. In the vicinity of this border (1‐20 µm), CNT fibers beat any other know conductor due to their strength and flexibility since metal wires are fragile and carbon fibers or graphitic intercalation compounds are brittle. Thus, ampacity is strongly influenced by fiber diameter with a near inverse dependence (Figure 6d) across a wide range of diameters and materials; electrical conductivity is also important with a square root dependence (Figure 6c). Data correlating CNT burn temperatures and ampacity are not available in sufficient quantity to draw any significant conclusions, but we would expect the two to be highly correlated.

**Table 7:**
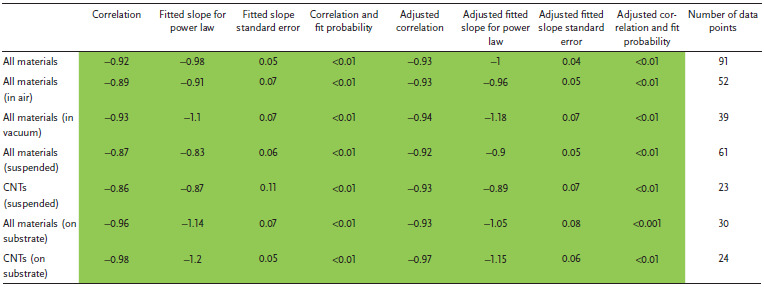
Log ampacity versus log diameter

Ampacity measurement protocols such as probe separation, probe configuration, background atmosphere, or whether the CNTs were laid on substrates or suspended vary across the literature. If ampacity values are to be compared across groups, measurement should be done on suspended fibers (to avoid heat dissipation via substrates^[^ ^168^ ^]^) with sufficiently large separation between the probes (so that the measurement becomes independent of probe separation^[^ ^221^ ^]^). Specific applications, such as interconnects, might require ampacity measurement on substrate, but these values can be higher than expected due to complicated heat‐flow.

### 3.3 Density and Specific Properties

**Figure 7:**
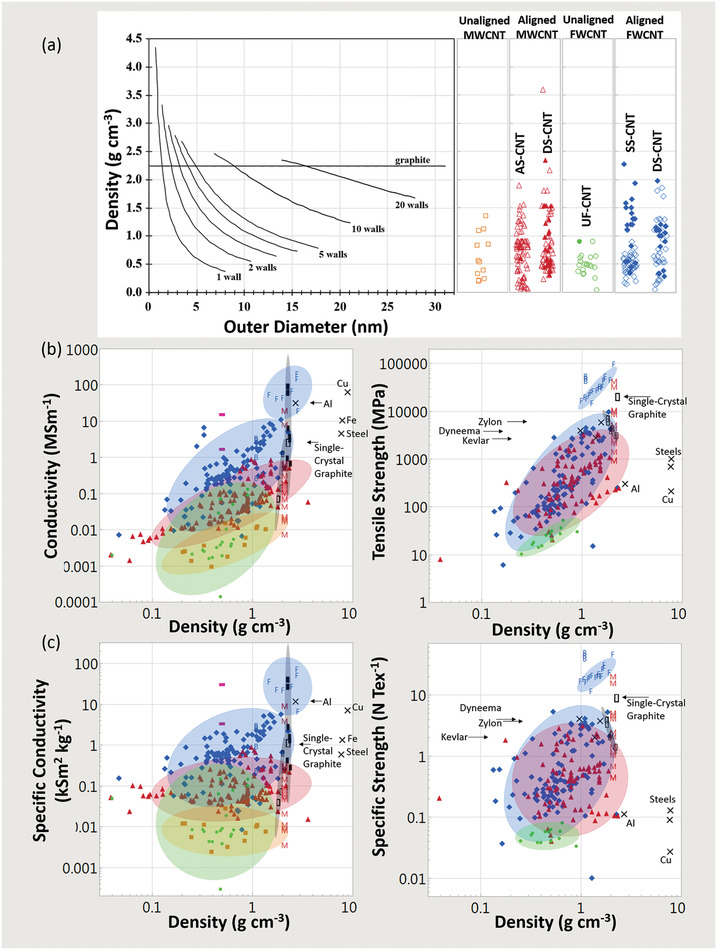
Density and density normalized characteristics for CNTs and benchmarks. a) Theoretical density of an individual CNT, as a function of diameter and wall number. Reproduced with permission.^[^ ^266^ ^]^ Copyright 2010, Elsevier. Alongside is the density of various CNT textiles. b) Conductivity and tensile strength as a function of density; c) specific conductivity and specific strength as a function of density. Key: 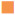) unaligned MWCNT material; 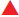) aligned MWCNT materials; 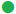) unaligned FWCNT materials; 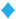) aligned FWCNT materials; 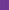) conductive polymers; 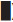) graphitic intercalation compounds; 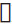) carbon fiber and graphite. M, F, B indicate individual MWCNTs, FWCNTs, and CNT bundles respectively. “*x*” indicated annotated benchmarks. Only in (a) do filled in shapes indicate doped materials. Ellipses help identify trends and are adjusted to cover 90% of the points. For the production method subcategories: AS‐CNT (derived from CNT forest arrays); UF/AF‐CNT (unaligned/aligned CNT film by filtering CNTs suspended in a fluid); DS‐CNT (aligned CNT materials directly extracted from FC‐CVD reactors); SS‐CNT (aligned CNT materials by extruding CNT solutions or suspensions into a coagulant).

CNT materials become more attractive when their low density is considered.^[^ ^11^ ^]^ The theoretical density of an individual CNT (provided by^[^ ^266^ ^]^) is depicted in **Figure** **7**a. Density increases with decreasing diameter and increasing numbers of walls, converging to that of graphite at 2.23 g cm^−3^. Note that there is a small effect from different helicities yielding different lattice constants not accounted for here.^[^ ^133^ ^]^ Another study^[^ ^267^ ^]^ has a similar analytical plot of bundle density versus tube diameter and wall number. In experimental studies on individual CNTs, a 15 nm diameter MWCNT was 1.74 g cm^−3[^ ^268^ ^]^ and a 49 nm diameter MWCNT was 2.09 g cm^−3^;^[^ ^133^ ^]^ an individual SWCNT was 2.13 g cm^−3^.^[^ ^133^ ^]^ A bundle of 0.8 nm diameter CNTs has a density of 1.16 g cm^−3^.^[^ ^269^ ^]^ In a careful gradient centrifugation study that took into account catalyst contaminants,^[^ ^133^ ^]^ the bundle density was 1.87 g cm^−3^ for an average CNT diameter of 1.44 nm. Next to the theoretical density plot are the measured bulk densities of various CNT materials (Figure 7a). Aligned CNT materials are denser than unaligned. Bulk densities are lower than the theoretical individual CNT densities due to impurities, such as amorphous carbon and residual catalyst, and packing factor associated with alignment of the microstructure. The density of graphitic intercalation compounds approximately equals that of graphite; as chemical species intercalate and weight increases, they simultaneously push the graphene planes apart and increase the volume nearly proportionally.^[^ ^18^, ^79^ ^]^

Specific conductivity and strength: Individual studies show that increasing CNT fiber density increases strength and conductivity.^[^ ^270^, ^271^, ^272^ ^]^ In particular, with dry spinning processes such as forest growth or FC‐CVD, densification is accomplished by soaking and evaporation in a solvent, such as acetone or ethanol.^[^ ^92^, ^209^, ^273^ ^]^ Mechanical densification can include twisting^[^ ^25^, ^194^, ^224^, ^274^ ^]^ (increasing strength by a factor of approximately two^[^ ^192^ ^]^), passing through wire‐drawing dies (increasing conductivity by a factor of three to ten^[^ ^16^, ^182^ ^]^) or from rollers (increasing conductivity a factor of two to ten^[^ ^26^, ^196^, ^275^ ^]^). Figure 7b shows positive correlation between density and both conductivity and tensile strength for all CNT categories; correlation values and power‐law exponents are shown in **Tables** **8** and **9** and are maintained after the weighted adjustment. Similar logarithmic plots of fiber properties versus density can be found here,^[^ ^200^ ^]^ although different CNT classifications and datasets are considered. In terms of a power‐law, we would expect a linear proportionality with density if the property enhancement was trivially from adding more material. Indeed power‐law exponents near unity are observed, except for aligned FWCNT material where the exponents are greater than unity.

**Table 8:**
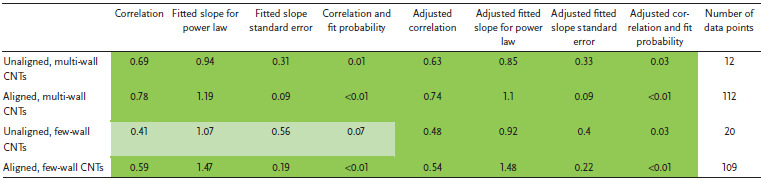
Log conductivity versus log density

**Table 9:**
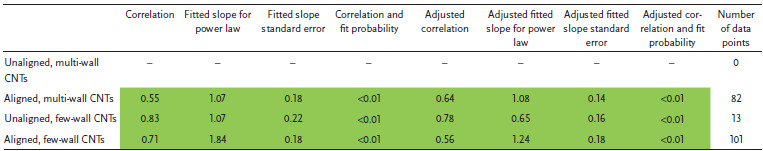
Log tensile strength versus log density

It has been explained^[^ ^271^, ^272^ ^]^ in individual studies that conductivity increases with density primarily from more material and conduction paths being present in a given cross‐section, opposed to any intrinsic transport enhancement. The conductivity divided by density, or specific conductivity, largely remains unchanged after densification in these studies. Specific conductivity is a particularly relevant metric for weight‐critical applications such as aerospace^[^ ^180^ ^]^ and overhead transmission lines.^[^ ^18^ ^]^ It is a more useful metric for CNT materials because, unlike absolute conductivity, it accounts for the substantial variances in density between CNT materials. Unlike absolute conductivity, the calculation of specific conductivity does not require measuring fiber cross‐section, which can be irregular and variable in these materials.^[^ ^238^ ^]^ All that is required is measuring the fiber's mass per unit length and resistance per unit length, according to Equation (4 )^[^ ^180^ ^]^

(4) $$\begin{array}{c} \text{Specific}\;\text{conductivity}\;={\left(\frac{\mathrm{Resistance}\;\left(\text{Ohms}\right)}{\text{Length}\;\left(\text{mm}\right)}\frac{\text{Mass}\left(\text{mg}\right)}{\text{Length}\;\left(\text{mm}\right)}\right)}^{-1}\; \end{array}$$

This makes specific conductivity easier to measure than absolute conductivity. Here for specific conductivity we use units of kS m^2^ kg^−1^, which is equivalent to the more natural units of MS m^−1^ (g cm^−3^)^−1^, and a value of unity is also approximately the value of single‐crystal graphite.

Figure 7c shows the relationship between specific conductivity and density for all CNT categories. The highest specific conductivity CNT category is again the doped, aligned FWCNT material. Within this category, when doped, acid‐extruded fibers (SS‐CNTs) have higher values ranging from 0.45 to 5.64 kS m^2^ kg^−1[^ ^15^, ^29^, ^168^, ^170^, ^180^, ^181^, ^276^, ^277^ ^]^ compared to directly spun FC‐CVD derived material (DS‐CNTs) with its best values so far clustered from 1.24 to 2.27 kS m^2^ kg^−1^.^[^ ^26^, ^27^, ^182^, ^218^, ^278^, ^279^ ^]^ The maximum values are approaching those of copper (6.7 kS m^2^ kg^−1^), although are a still factor of two to three away from a more appropriate and cheaper benchmark, aluminium (13 kS m^2^ kg^−1^). One often cited doped FWCNT paper^[^ ^14^ ^]^ reported even higher specific conductivity values then those given here. However, it is likely that these high specific conductivity values may have been overestimated because density was calculated by weighing multiple microfibers at once and, assuming they had the same density, dividing the total weight by the fiber number to get an average microfiber weight;^[^ ^15^ ^]^ further, such high values have not been reproducibly obtained when repeating their method.^[^ ^280^ ^]^ For the older advanced carbon conductors, although small, discontinuous, and not air‐stable, iodine‐doped polyacetylene is estimated to be between 15 and 30 kS m^2^ kg^−1[^ ^82^ ^]^ and the most conductive graphitic intercalation compounds can range between 26 and 40 kSm^2^ kg^−1^,^[^ ^37^, ^49^, ^79^ ^]^ although many stronger, more practical graphitic intercalation compounds can reside between 2 and 3 kS m^2^ kg^−1^.^[^ ^18^ ^]^ In order to assess the upper bound value for bulk CNTs, the specific conductivity of an individual FWCNT bundle was estimated by taking bundle conductivities (given in Figure 3) and dividing by the bundle density measured in a specific report.^[^ ^281^ ^]^ This was found to be 1 to 3.3 kS m^2^ kg^−1^, which is comparable to that of single‐crystal graphite (1.1 kS m^2^ kg^−1^). By contrast, individual SWCNT conductivity was found to be much higher at 25 to 154 kS m^2^ kg^−1^; further details on the estimation of specific conductivity of these intrinsic structures may be found here.^[^ ^282^ ^]^

Specific strength is calculated by taking the ultimate tensile strength and dividing by density. Figure 7c shows specific strength plotted against density. The specific strength of aligned MWCNT and FWCNT material are substantially greater than unaligned FWCNT material, structural metals, and conductive metals. Within the aligned FWCNT category, the leading material derived from direct extraction from FC‐CVD (DS‐CNT) clusters from 1.6 to 6.4 N tex^−1^.^[^ ^14^, ^26^, ^27^, ^28^, ^196^, ^209^, ^210^, ^218^ ^]^ Note that N tex^−1^ is dimensionally equivalent to GPa (g cm^−3^)^−1^. Two reports in DS‐CNT exceed the range of carbon fiber and synthetic fiber (4.4 to 5.2 N tex^−1^).^[^ ^27^, ^196^ ^]^ A recent FWCNT paper^[^ ^28^ ^]^ reached 2.3 N tex^−1^ and, after an acid‐based stretching post‐process, reached 6.4 N tex^−1^. This is record breaking for any material fiber, although they did not report the density. One often cited FC‐CVD paper^[^ ^92^ ^]^ has a value nearly double even this, although the authors explained that this was a one‐off result with zero gauge length, which makes proper measurement problematic due to the non‐uniform stress distribution throughout the cross section.^[^ ^205^ ^]^ The best acid‐extruded FWCNT material (SS‐CNT) ranges from 1 to 2.1 N tex^−1^.^[^ ^15^, ^29^, ^170^, ^181^ ^]^ In terms of material properties, specific strength is the only characteristic where DS‐CNT fibers are systematically better than SS‐CNT fibers (Figure S7, Supporting Information). DS‐CNTs can be particularly long; moreover, in FC‐CVD, oligomeric by‐products can hold CNTs together as a kind of composite^[^ ^205^, ^209^ ^]^ and is a topic that warrants further study. The best commercial synthetic fibers range from 2.0 (Kevlar) to 3.7 (Zylon^[^ ^283^ ^]^) to 4.0 N tex^−1^ (Dyneema); the specific strength of the best commercial carbon fiber ranges from 1.9 to 3.9 N tex^−1^. We also note that aligned CNT materials are also stronger on a weight basis than graphitic intercalation compounds. For graphitic intercalation compounds formed from graphitized carbon fiber, while highly conductive, they are problematically unbendable and brittle^[^ ^18^ ^]^ and there is little mechanical data. Graphitic intercalation compounds made from a carbon host that is stronger, though more disordered, sacrifices its post‐doped conductivity for strength; reports of its specific strengths range from 0.5 to 1 N tex^−1^ for specific conductivities 2 to 4 kS m^2^ kg^−1^.^[^ ^18^ ^]^ In order to assess the maximum possible bulk CNT specific strength, the specific strength of an individual FWCNT can be 49 N tex^−1^ and an individual FWCNT bundle 27 to 30 N tex^−1^,^[^ ^201^ ^]^ which are all high intrinsic values compared to single‐crystal graphite (8.8 N tex^−1^).^[^ ^18^ ^]^

Correlation data between density and the conductivity and strength specific properties are provided in **Tables** **10** through **11**. Compared to the absolute properties, most correlations with density have disappeared or become intermittent with the weighted adjustment. The exception is specific conductivity for aligned FWCNTs, where there is still a significant correlation both before and after the weighted adjustment. Thus, this survey of the literature shows intrinsic enhancement of conductivity with increasing density for FWCNT materials, while for other cases increasing density improves properties by simply adding more material. For conductive polymers, also, it was determined that increasing density had little if any intrinsic benefit.^[^ ^82^ ^]^

**Table 10:**
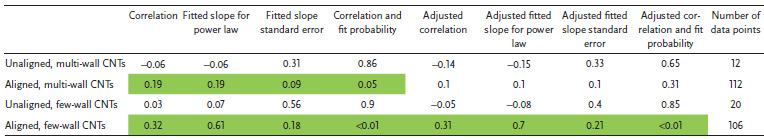
Log specific conductivity versus log density

**Table 11:**
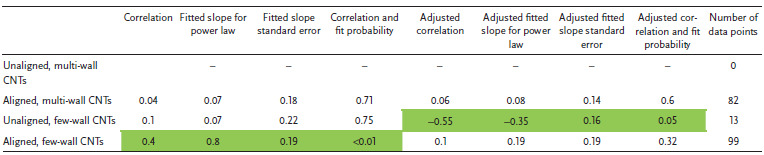
Log specific strength versus log density

Coming to the question of density dependence of ampacity, following the standard Fuse law (Equation (2)), Cress et al. showed that *I*_max_ ∝ *D*_L_^0.75^ where *D*_L_ is the linear density of the fiber. However, if the corrected law (Equation (3)) is used, then *I*_max_ ∝ *D*_L_^0.544^ or *J*_max_ ∝ *D*_L_^−0.457^. These equations can be written in terms of *D*_V_ with the same magnitude of the exponent, but with an opposite sign. The data available in the literature are insufficient to verify the precise dependence on density.

### 3.4 Purity

Impurities in CNT fiber, such as amorphous carbon and left‐over iron catalyst, come from the CNT growth process. Growth of smaller diameter CNTs are typically accompanied by a greater fraction of amorphous carbon and catalytic impurities.^[^ ^284^ ^]^ For example, SWCNTs grown via chemical vapor deposition typically have an impurity weight fraction ranging from 50% to 80% of the total as‐is yield; for MWCNTs the range can be lower, from 1% to 70%.^[^ ^284^ ^]^ Specific to the FC‐CVD process, the typical weight of amorphous carbon fraction ranges from 4% to 20%^[^ ^209^ ^]^ and residual catalyst from 1.3% to 15%.^[^ ^26^, ^209^, ^285^ ^]^ When FC‐CVD is used to produce SWCNTs of particularly narrow diameter distribution and a high degree of graphitic perfection, the amorphous carbon content can be less than 5% and residual catalyst weight can approach 60%.^[^ ^218^, ^261^ ^]^

In the best case, impurities add dead‐weight to CNT conductors and, more often, they hinder the intrinsic electronic transport. For CNTs fibers wet‐spun from super‐acid solutions, residual catalyst above 5% by weight prohibits alignment of the CNT microstructure and the formation of homogeneous fibers.^[^ ^29^, ^170^ ^]^ Another study showed that the specific conductivity of super‐acid spun fibers increased from 0.04 to 1 kS m^2^ kg^−1^ after conversion from unpurified SWCNTs (CNTs comprising 20–30 wt% of the carbonaceous mass) to purified SWCNTs (CNTs comprising 99 wt% of the carbonaceous mass).^[^ ^180^ ^]^ For FC‐CVD‐derived CNT fibers, purification resulting in catalyst less than 1% by weight led to a conductivity of 2 MS m^−1^;^[^ ^14^ ^]^ in another report, purification to impurity levels below 10% by weight led to a conductivity of 1.6 MS m^−1^.^[^ ^182^ ^]^

Chemical‐based purification of CNTs comes at the expense of adding defects and lowering the yield; this becomes particularly challenging when oxidation rates of impurities and CNTs are similar.^[^ ^284^ ^]^ Acid‐soaking,^[^ ^284^ ^]^ such as hydrochloric or nitric acid, or chlorine gas exposure^[^ ^286^ ^]^ is required to dissolve metal catalyst in a scalable and uniform way. Residual catalyst typically has a protective carbon shell that hampers straight forward removal with harsh chemical exposure.^[^ ^13^, ^284^, ^285^, ^286^, ^287^ ^]^ This carbon shell must be oxidized away first via gas‐phase oxidation (baking in air) or liquid‐phase oxidation (soaking in hydrogen peroxide). For SWCNT powders at 500 °C in air, for example, the oxidation rate of the catalyst's protective carbon shell roughly matches the oxidation rate of the SWCNTs.^[^ ^287^ ^]^ Amorphous carbon typically has a faster oxidation rate than CNTs because of its dangling bonds and disordered nature^[^ ^284^, ^288^ ^]^ (burning initiating between 370 and 400 °C^[^ ^285^, ^289^ ^]^). Smaller diameter SWCNTs and defective CNTs oxidize next due to greater bond curvature or activation sites (burning initiating between 440 and 550 °C^[^ ^289^ ^]^). DWCNTs and MWCNTs generally start oxidizing last (burning between 500 and 800 °C^[^ ^290^, ^291^, ^292^ ^]^). However, this trend is not universally true, and in several cases authors using FC‐CVD production^[^ ^27^, ^261^ ^]^ have noticed high quality SWCNT material burning at higher temperature than MWCNT populations. This observation suggests that defects can determine oxidation temperature rather than CNT curvature. The presence of residual catalyst also lowers the oxidation threshold for CNTs. For SWCNT film with residual catalyst present, for example, they completely burn in air at 425 °C within 30 min; after purification, the SWCNTs are stable under these conditions.^[^ ^287^ ^]^ Heating CNTs dynamically over a range of temperatures, as opposed to isothermally, has been found to be a particularly effective method of removing carbon‐based impurities.^[^ ^289^, ^293^ ^]^

Due to the great variety of CNT materials and requirements there is no one standard purification technique; an extensive purification review is given in.^[^ ^284^ ^]^ For the highest conductivity aligned FWCNT fibers surveyed here however, there is a common theme of an initial oxidization step consisting of some combination of air baking (400 to 560 °C) and soaking in hydrogen peroxide (30% to 37%) or nitric acid. Concurrent with or following this is a soaking in hydrochloric acid (37%) with the entire process taking tens of hours to days.^[^ ^14^, ^180^, ^182^, ^285^ ^]^ Because there is typically no de‐doping step, such as a vacuum bake, it is difficult to differentiate between improvement due to purification and that due to doping.

### 3.5 Anisotropy and Microstructure Alignment

Having shown that CNT material with an aligned microstructure has substantially better properties than when completely unaligned, we now discuss the continuum of microstructure alignment between these extremes. Microstructure alignment was a critical and implicit parameter for the conductivity and pre‐doped strength of conductive polymers. Mechanical stretching of polyacetylene increased the film length by a factor of six to fifteen, leading to improvement in microstructure alignment and proportional increase in conductivity after doping.^[^ ^82^, ^197^ ^]^ CNT reports also showed that stretching improved microstructure alignment and material properties,^[^ ^186^, ^294^, ^295^, ^296^, ^297^ ^]^ although the stretching was less dramatic than for conductive polymers. For example, stretching a MWCNT film with solvent led to a 22% to 40% increase in length and a factor of two to ten increase in conductivity.^[^ ^294^, ^298^, ^299^ ^]^ For a SWCNT film, a stretch increase of 80% increased conductivity by a factor of four times.^[^ ^295^ ^]^ In other cases, stretching CNT films with chlorosulfonic acid led to a 10% to 30% length increase and tensile strength increase a factor of two to three^[^ ^27^, ^28^, ^300^ ^]^ (leading to record specific strengths in any synthetic fibers). Again, with chlorosulfonic acid, for SWCNT films with a high degree of graphitic perfection, 150% to 200% increase in stretched length was possible. Conductivity scaled linearly with stretch length, although this trend saturated as the de‐doped specific conductivity approached the value for single‐crystal graphite.^[^ ^218^ ^]^ Similar results were also later obtained with the same degree of stretching and de‐doping.^[^ ^176^ ^]^ It is likely that CNT fibers directly extracted from FC‐CVD reactors (DS‐CNT) will always require similar post‐processing to further align, densify, and dope the CNT assembly in order to be competitive with acid‐extruded material (SS‐CNT).^[^ ^28^, ^60^ ^]^

Since the degree of microstructure alignment is frequently reported using different metrics, it was difficult to obtain a consensus in the meta‐analysis. Techniques to measure CNT microstructure alignment generally fall into three classes: conductivity anisotropy ratio, diffraction techniques, and Raman spectroscopy techniques. The conductivity anisotropy ratio is advantageous in that it probes throughout the entire material's bulk. However, diffraction and Raman techniques are more prevalent in the literature, although they just measure a small surface region. For Raman, the probed area is the typically micrometer wide spot size of the laser; for X‐ray diffraction the typical spot size diameter is several millimeters and penetrates deeper into the bulk.^[^ ^294^ ^]^ For example, the X‐ray attenuation coefficient of CuKα radiation for highly oriented pyrolytic graphite is 10 cm^−1[^ ^301^ ^]^ and means that any attenuation happens at scales well beyond the thickness of most CNT materials.

**Figure 8:**
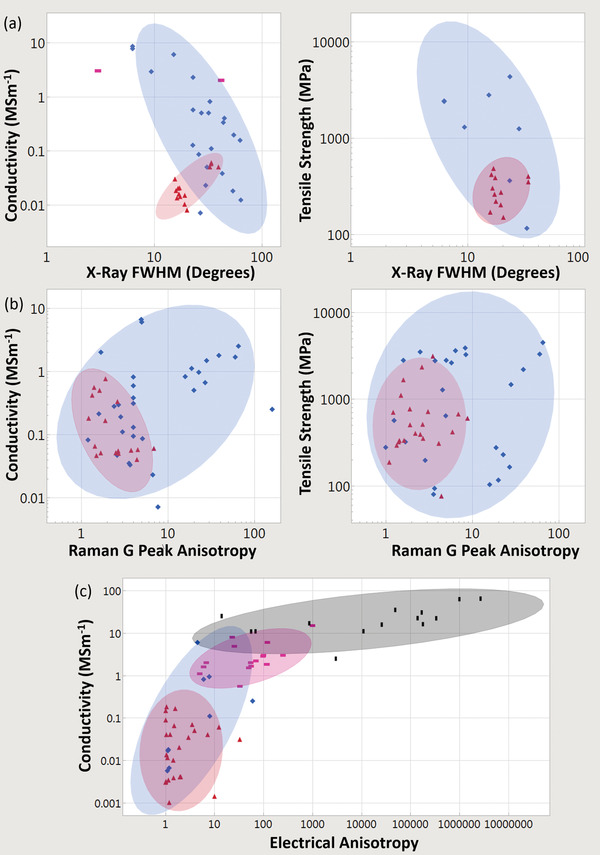
a–c) Material properties versus different microstructure alignment/anisotropy metrics across the literature: a) FWHM of the intensity variation measured in the azimuthal circle of a Bragg peak in X‐ray diffraction; b) Raman spectroscopy's G peak anisotropy; and c) electrical anisotropy. Key: 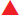) aligned MWCNT materials; 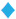) aligned FWCNT materials; 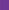) conductive polymers; 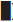) graphitic intercalation compounds. Ellipses help identify trends and are adjusted to cover 90% of the points.

The conductivity anisotropy ratio is the conductivity measured parallel to direction of microstructure alignment divided by the conductivity measured perpendicular this direction. A four‐probe set‐up is the standard measurement configuration provided the positioning of the multimeter probes ensures a uniform current distribution. With the four‐probe setup, for an anisotropic conductor, it is important that the aspect ratio of the sample (its length divided by its width) be at least as large at the square root of its conductivity anisotropy ratio.^[^ ^36^ ^]^ The conductivity anisotropy can also be obtained by four probes positioned on the corner of a square of material (the Montgomery method^[^ ^294^ ^]^), as well as without physical contact using polarized microwaves^[^ ^302^ ^]^ or THz radiation.^[^ ^9^, ^303^ ^]^ **Figure** **8**c shows the conductivity anisotropy values for CNTs, conductive polymers, and graphitic intercalation compounds against their conductivity. For the most conductive graphitic intercalation compounds, where doping chemical species reside between each graphene plane, this ratio can be as large as ≈10^6^. In these cases of extreme anisotropy, four‐probe DC measurement does not lead to a uniform current distribution and conductivity must be measured using AC inductive techniques.^[^ ^79^, ^162^, ^304^ ^]^ Conductive polymers with conductivities larger than 1 MS m^−1^ typically have anisotropy values of ≈100^[^ ^82^ ^]^ and, for the highest conductivity conductive polymers (15 MS m^−1^), the anisotropy can reach ≈1000.^[^ ^30^ ^]^ For undoped graphite, the anisotropy ratio depends on graphitic quality with values ranging from 2500 to 10 000.^[^ ^79^, ^162^, ^305^ ^]^ Anisotropy ratios are lower for aligned MWCNT materials with a maximum found in the literature of 33;^[^ ^134^, ^225^, ^294^, ^306^, ^307^ ^]^ for aligned FWCNT materials, the maximum value is 60 (Figure 8c). Bucky papers made from aligned SWCNTs (AF‐CNTs) can achieve near perfect alignment and packing when filtering conditions are carefully controlled. This results in the anisotropy ratio of 60, although their conductivity is relatively low (0.25 MS m^−1^) because of short SWCNT length (<500 nm).^[^ ^9^, ^188^, ^189^, ^190^ ^]^ Short lengths were necessary to increase stiffness and avoids kinks and bending when forming the bucky paper dispersion.^[^ ^19^ ^]^ Individual CNT studies^[^ ^185^, ^186^, ^294^ ^]^ found connection between CNT conductivity, strength, and anisotropy. Using an anisotropy ratio based on specific conductivity, for FWCNT mats made from FC‐CVD, a specific study from our group found anisotropy ratio values ranged from one to 30 and was tightly correlated with specific conductivity and specific strength.^[^ ^219^ ^]^ Surveyed across the meta‐analysis however, there is some positive correlation between conductivity and anisotropy ratio for the aligned FWCNT material, although it does not survive the weighted adjustment, and there is none for the aligned MWCNT material (**Table** **12**). This is possibly because the conductivity anisotropy is not widely reported as a metric in the literature, apart from the significant correlation found in specific studies.

**Table 12:**
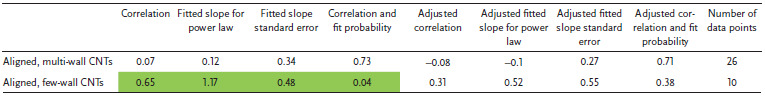
Log conductivity versus log electrical anisotropy

Measuring microstructure alignment using X‐ray diffraction has been common in synthetic fiber and carbon fiber research, although the application of X‐ray diffraction for CNT cable development^[^ ^15^, ^26^, ^181^, ^185^, ^186^, ^209^, ^276^, ^277^, ^278^, ^308^, ^309^, ^310^ ^]^ has been not as widespread. Individual CNTs may have a degree of graphitic perfection, although this perfection is largely independent of their degree of ordered assembly into large bundles. This contrasts with carbon fibers where graphitic perfection and crystalline order in the fiber are linked. The lower degree of crystalline order in CNT fibers leads to a weaker X‐ray signal and its lower reliability as an analytic tool.^[^ ^60^, ^311^ ^]^ For X‐ray diffraction studies across the literature, Figure 8a shows the conductivity and tensile strength of aligned CNT material as a function of the full‐width‐at‐half‐maximum (FWHM) of the intensity variation measured around the azimuthal circle of some selected 2θ scattering peak; correlation data are provided in **Tables** **13** and **14**. Here we do not differentiate between the type of scattering peak, although they originate typically from order induced by bundle formation^[^ ^169^, ^186^, ^312^ ^]^ or the interlayer spacing of repeating shells in multiwall CNTs.^[^ ^313^ ^]^ For aligned FWCNT materials, there is the expected negative correlation between conductivity and FWHM that is maintained after the weighted adjustment. For aligned FWCNT strength, no correlation with FWHM is apparent. For aligned MWCNTs, there is an unexpectedly positive correlation for conductivity that is maintained after the weighted adjustment, as well as possibly marginal positive correlation for strength after the weighted adjustment. Inspecting the graphs in Figure 8a, however, it appears as if this positive correlation might be spurious and result from a cropping out of points that possibly obscures the expected negative correlation. This will be discussed in a broader context below.

**Table 13:**
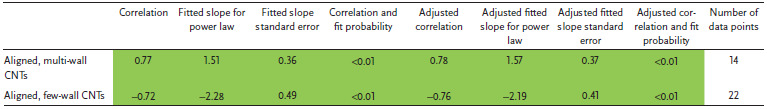
Log conductivity versus log of the FWHM of azimuthal scan peak in X‐ray diffraction

**Table 14:**
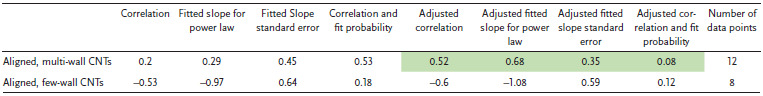
Log tensile strength versus log of the FWHM of the azimuthal scan peak in X‐ray diffraction

The smallest FWHMs observed for aligned FWCNTs so far fall between 6° and 10°. For comparison, the FWHM of the most conductive iodine‐doped polyacetylene generally span from 3° to 21°^[^ ^82^, ^197^, ^314^ ^]^ (the FWHM and the associated azimuthal scan are typically taken from the 110 or 200 Bragg peaks of polyacetylene^[^ ^82^ ^]^). One conductive polymer, for example, had a conductivity of 2 MS m^−1^ with a FWHM of 21°.^[^ ^314^ ^]^ While not a diffraction technique, similar azimuthal angle peak analysis may be generated by considering the Fourier transform of scanning electron microscopy (SEM) images in both the horizontal and vertical directions of the photograph.^[^ ^9^, ^209^ ^]^ For the near perfectly aligned, perfectly packed bucky paper,^[^ ^9^ ^]^ the SEM FFT alignment FWHM was 3°, although the conductivity was lower due to the shortness of the CNTs.

Since 1946, the Hermans orientation parameter has been an established metric for polymer fibers that reduces the azimuthal angle peak into a scalar which measures the degree of uniaxial orientation using an analytical expression given in other reports.^[^ ^209^, ^294^, ^311^ ^]^ The Hermans orientation parameter is calculated relative to some pre‐selected axis and spans from –0.5 (indicating alignment in a plane perpendicular to the pre‐selected axis), to zero (indicating no alignment with the pre‐selected axis), to one (indicating maximum alignment with the pre‐selected axis). The highest FWCNT fiber values have Hermans orientation parameters approaching one (ranging from 0.974 to 0.99) and conductivities from 2.9 to 8.5 MS m^−1^.^[^ ^15^, ^170^, ^181^ ^]^ Once the material has a good degree of microstructure alignment, the Hermans orientation parameter becomes only weakly sensitive to further enhancement of alignment; In addition, it only applies to 3D diffraction characterization. When the microstructure alignment characterization is accomplished by a 2D surface scan (such as with a scanning electron microscope), the typical Hermans orientation parameter expression must be modified as provided in^[^ ^209^ ^]^ or requires analysis of the more general orientation distribution function.^[^ ^60^, ^311^ ^]^ Other reports^[^ ^186^, ^294^ ^]^ explain that alignment of catalyst, amorphous carbon, and voids in the microstructure results in the overestimation of the Hermans orientation parameter. Further, the Hermans orientation parameter may depend strongly on the peak fitting functions used and is affected by the background signal. By contrast, consideration of FWHM is simpler and does not require the fitting of functions, although is less mathematically robust as compared to orientation parameters.^[^ ^311^ ^]^ For these reasons, the literature would benefit if FWHM was always reported with other diffraction‐based metrics, with the understanding that FWHM is a more qualitative metric than quantitative.

Raman spectroscopy also provides a useful approach to measure microstructure alignment. Many reports acquire a sense of the anisotropy simply by changing the direction of polarization of the inbound Raman laser to the sample, with the polarization parallel and then perpendicular to the direction of microstructure alignment. The intensity change of some Raman spectra feature, typically the *G* peak at ≈1580 cm^−1^, indicates the degree of microstructure alignment. For aligned MWCNTs, Raman anisotropy values range between 1 and 10^[^ ^134^, ^209^, ^274^, ^275^, ^315^, ^316^, ^317^ ^]^ and, again for aligned FWCNTs, they range higher between 1 and 107.^[^ ^14^, ^27^, ^28^, ^181^, ^186^, ^190^, ^196^, ^209^, ^218^, ^277^, ^318^ ^]^ For the nearly perfectly packed and aligned SWCNT bucky paper, the Raman spectroscopy alignment ratio rose to 160.^[^ ^9^ ^]^ Within specific studies it has been shown that increasing the Raman anisotropy value leads to increased material properties.^[^ ^92^, ^294^, ^316^, ^319^ ^]^ This Raman technique does not consider the CNT's significant optical absorption of the inbound laser, which itself is alignment‐dependent. Further, this technique does not take into account polarized Raman‐shifted radiation going from the sample to the spectrometer. These factors make comparison across different studies difficult and render Raman anisotropy values more of a qualitative indicator of anisotropy than relating directly to a physical material property. Addressing these subtleties, more sophisticated treatments^[^ ^9^, ^181^, ^276^ ^]^ of Raman spectroscopy yield physical alignment metrics, although are not widely enough utilized in the literature for analysis here. Still, we plot the simple Raman G peak anisotropy values across the literature (Figure 8b) versus conductivity and strength; correlation data are provided in **Tables** **15** and **16**. For aligned FWCNT materials, while no correlation is apparent for strength, there is the expected positive correlation between conductivity and Raman anisotropy. The correlation does not survive the weighted adjustment however and this signals undue bias from specific studies. For aligned MWCNT materials, again there is the opposite behavior of what was expected—a negative correlation between conductivity and Raman *G* peak anisotropy that is marginally maintained after the weighted adjustment.

**Table 15:**
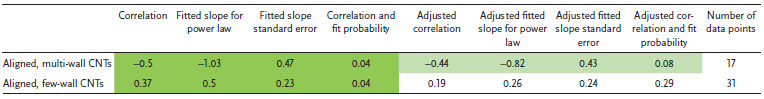
Log conductivity versus log of the Raman anisotropy in the G peak

**Table 16:** Log tensile strength versus log of the Raman anisotropy in the G peak

	Correlation	Fitted slope for power law	Fitted slope standard error	Correlation and fit probability	Adjusted correlation	Adjusted fitted slope for power law	Adjusted fitted slope standard error	Adjusted correlation and fit probability	Number of data points
Aligned, multi‐wall CNTs	0.03	0.05	0.32	0.88	−0.16	−0.28	0.39	0.48	22
Aligned, few‐wall CNTs	0.11	0.13	0.24	0.58	−0.25	−0.34	0.26	0.21	27

### 3.6 Average CNT Dimensions and Graphitic Perfection

While the average length, diameter, and graphitic perfection of individual CNTs influence the electrical, thermal, and strength of the bulk CNT cable, the average dimensions of the next level of agglomeration, the CNT bundle, are similarly important. They are analogous to the crystal grains found in graphite and graphene, where larger crystal grains result in less scattering at grain boundaries and overall higher electricity conductivity.^[^ ^119^, ^320^, ^321^ ^]^ There is a point however where crystal grain dimensions become substantially larger than the average scattering length mean‐free‐path, say from phonons, and increasing the crystal domain size no longer increases conductivity in these undoped materials. An example of this is pristine highly oriented pyrolytic graphite (HOPG), where the 1 µm average crystallite size is larger than the characteristic scattering length.^[^ ^49^ ^]^ For graphitic intercalation compounds, the highest degree of graphitic crystal size, density, and in‐plane conductivity was required of the host graphite structure before the highest post‐intercalated conductivities (at times surpassing copper) could be achieved after doping.^[^ ^32^, ^33^, ^35^, ^36^, ^37^ ^]^ Larger crystallite domains, even when much larger than the scattering length, increased post‐doped conductivity because larger crystallite domains led to faster and more complete intercalant uptake; smaller crystallite domains trap and impede intercalant species.^[^ ^49^ ^]^

Concerning CNT material with an unaligned microstructure, a numerical simulation^[^ ^322^ ^]^ showed that the upper‐bound network conductivity depends on the CNT dimensions according to σ ∝ *V*_f_(*L*/*d* ^2^) where σ is the conductivity, *V*_f_ is the volume fraction of the network, *L* is the length of the CNT structure and *d* is the CNT structure diameter. In this treatment, ideal CNT contacts and purely ballistic conduction within CNTs were assumed. In an analysis^[^ ^167^ ^]^ for a diffusive regime where again CNT junctions dominate the transport in the unaligned network, σ ∝ *L^x^*/*d* ^2^ where *x* is an exponent between 0 and 2.48. As junction resistance becomes less significant and most of the network resistance is distributed over the CNTs themselves, the exponent tends toward zero;^[^ ^167^ ^]^ this illustrates that at some point in material development, when most of the overall fiber conductivity is from the intrinsic CNT conductivity, increasing CNT length will lead to diminishing returns in as‐is conductivity. The inverse dependence of *d* was explained by more conducting elements operating in parallel with smaller structure diameters, although assumes that the CNTs are perfectly rigid and that bundle diameter does not affect junction resistance. Experimentally,^[^ ^167^, ^323^ ^]^ for real‐world unaligned FWCNT networks where resistance contributions come from both intrinsic FWCNT structures and junctions that depend on diameter, conductivity scales according to *L* ^1.46^ and *d* ^−1.613^. These length studies were accomplished with relatively short, micrometer long SWCNTs. Experiments on much long length SWCNTs in unaligned films^[^ ^270^ ^]^ confirmed a weaker, though still positive, correlation between conductivity and average length; film conductivity only doubled going from a CNT length of 350 to 1500 µm.

While *d* is defined for CNT structures generally, specifically, smaller CNT bundle diameters *d*_B_ also lead to higher film conductivities and may have more specific considerations. As explained earlier, smaller bundles means more CNT junctions wired in parallel and more conduction paths.^[^ ^50^, ^119^, ^150^, ^152^, ^167^, ^324^ ^]^ Additionally, researchers^[^ ^158^, ^159^, ^160^ ^]^ demonstrated that the current density over a bundle is not uniform and only the outer CNTs in a bundle participate in the transport. Further, current traveling preferentially down a bundle may sometimes need to cross perpendicular to the bundle to get to the next node point. For comparison, with high‐quality graphite, the *a*‐axis conductivity (across the planes) is 2500 to 10 000^[^ ^79^, ^162^, ^305^ ^]^ greater than the *c*‐axis conductivity (through the planes). This large anisotropy may also apply similarly to the CNT bundle and could hinder uniform current distribution over its cross‐section. One research group,^[^ ^158^, ^324^ ^]^ using geometrical arguments to analyze the number of bundle junctions in an unaligned SWCNT network, found that conductivity follows a stronger relationship with diameter, σ  =  *K* *V*_f_^2^/(*R*_Bundle_ *d*_B_^3^) where *R*_Bundle_ is the average resistance between bundles and *K* is a proportionality factor incorporating CNT length according to *K* ∝ *L* ^1.7^. As a counterpoint however, another vein of experimental research into unaligned CNT networks found greater network conductivity from larger diameter CNTs.^[^ ^325^ ^]^ This was attributed to two factors: lower junction resistance from greater contact area^[^ ^166^ ^]^ (seemingly opposite to the finding from Nirmalraj et al.^[^ ^158^ ^]^) and a greater effectiveness of doping due to smaller bandgaps in larger diameter CNTs.^[^ ^326^ ^]^

**Figure 9:**
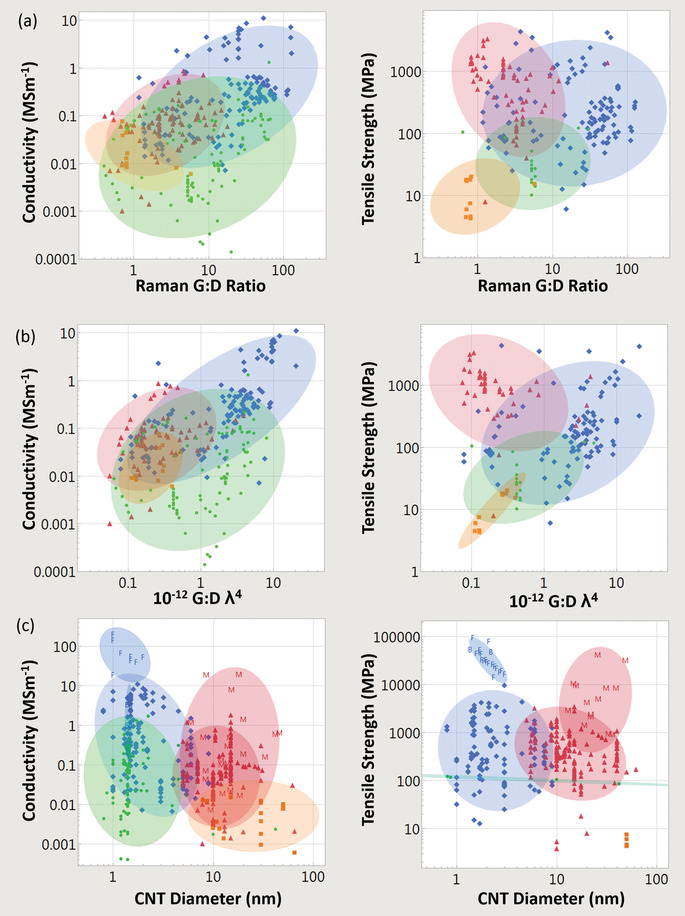
a–c) Conductivity and strength metrics versus Raman spectroscopy G:D ratio (a), Raman spectroscopy G:D ratio × λ^4^ (b), and CNT diameter (c). Key: 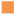) unaligned MWCNT material; 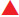) aligned MWCNT materials; 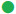) unaligned FWCNT materials; 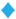) aligned FWCNT materials. M and F indicate individual MWCNTs and FWCNTs and B indicates individual bundles. Ellipses help identify trends and are adjusted to cover 90% of the points.

We now consider the impact of molecular dimensions on CNT material with aligned microstructure. Systematic studies of aligned FWCNT^[^ ^170^, ^181^, ^327^ ^]^ and MWCNT material^[^ ^307^ ^]^ found that conductivity and strength scale approximately linearly with CNT length (more precisely^[^ ^170^, ^181^ ^]^ aspect ratio). Earlier, we discussed that the room‐temperature mean‐free‐path will be unavoidably limited by phonons to approximately 1 µm. Significantly longer CNTs are still favorable to conduction in that they will decrease the number of CNT junctions and extrinsic resistance contributions. The strength is also expected to scale linearly with CNT length when sliding friction is the limiting force keeping aligned CNTs in place under tension.^[^ ^328^ ^]^ Coarse‐grained modeling^[^ ^207^ ^]^ showed that, in this regime when frictional sheer force between CNTs is the limiting force, the tensile strength is proportional to *Lf* where *L* is the CNT length and *f* is the frictional force per unit length, which can be increased by cross‐linking. This proportionality applies for various distributions of CNT lengths and positions within a bundle. A linear proportionality between CNT length and material properties generally will taper off as the intrinsic stress‐transfer limit is approached.^[^ ^12^, ^207^, ^328^ ^]^ It is possible the intrinsic properties are from the individual CNT, although in this report and elsewhere^[^ ^218^, ^329^ ^]^ we have discussed the bundle as the limiting intrinsic element. We note that only a handful of CNT fiber papers^[^ ^25^, ^26^, ^27^, ^168^, ^174^, ^175^, ^176^ ^]^ have approached the conductivity of single‐crystal graphite in the undoped state. While other papers^[^ ^170^, ^181^ ^]^ have shown improved conductivity with longer CNT length and higher aspect ratio, it is possible that other factors, such as bundle diameter, may still limit the transport in many CNT materials. Conductivity and strength should increase with smaller diameter CNTs, for similar reasons explained for unaligned CNTs (that is, an initial expectation of *d* ^−2^ dependence). An experimental study on aligned FWCNT materials did not find any influence from diameter;^[^ ^327^ ^]^ studies on large diameter aligned MWCNTs found stronger CNT materials with smaller diameters.^[^ ^204^, ^330^ ^]^ **Figure** **9**c shows conductivity and tensile strength as a function of CNT diameter across reported values in the literature; correlation data are provided in **Tables** **17** and **18**. When the diameter dataset is taken in its entirety without CNT categorization, there is a clear negative correlation between CNT diameter and both electrical conductivity and tensile strength (for quantification, see Figure S8, Supporting Information). For just the aligned FWCNT category, there is negative correlation between conductivity and diameter with a power‐law of ≈−1 that is maintained after the weighted adjustment. Less strong negative correlations between conductivity and CNT diameter are possibly present with the other categories, although only after the weighed adjustment. For correlation of strength with CNT diameter, there were insufficient data for the unaligned MWCNT material; for unaligned FWCNTs, a correlation was observed, although this may be spurious considering that only four data points are available. For aligned FWCNT material, there was no apparent correlation. For the aligned MWCNT material however, there was a statistically significant negative correlation that persisted after the weighted adjustment. Thus, correlation with diameter is somewhat intermittent when CNT categorization is taken into account and not as strong as simple analysis would suggest. While there are a number of possible explanations for the weaker than expected dependence on CNT diameter, one possible consideration is that the average bundle diameter, which is rarely reported, may be a limiting factor as discussed above.

**Table 17:**
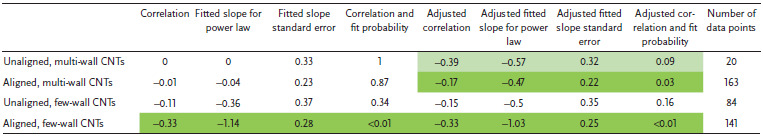
Log conductivity versus log CNT diameter

**Table 18:**
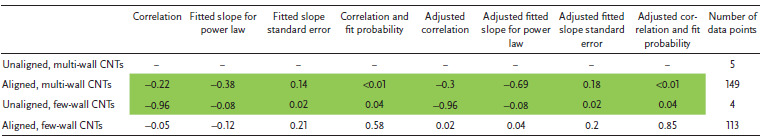
Log strength versus log CNT diameter

Individual FWCNTs in a powder morphology typically range in dimension as follows: length 0.4 to 20 µm, diameter 0.8 to 3.2 nm, and aspect ratio (*L*/*d*) 340 to 9600.^[^ ^331^ ^]^ For CNT fibers formed from superacid wet spinning (SS‐CNTs), any CNT aspect ratio is acceptable for processing provided there is sufficient graphitic perfection as well as initial quantity (>50 mg). The highest conductivity CNT material currently is an acid‐spun fiber at 10.9 MS m^−1^ consisting of CNTs 12 µm long with an aspect ratio of 6700.^[^ ^29^ ^]^ This has increased from a few years ago, where the same group produced a CNT fiber with a conductivity of 8.5 MS m^−1^ consisting of FWCNTs with aspect ratio 4400.^[^ ^170^ ^]^ The aspect ratio from FC‐CVD derived material (DS‐CNTs) has been challenging to quantify because of the long, tangled nature of the CNTs. One early approach involved long, statistical searches for CNT ends relative to number of CNTs seen under a transmission electron microscope; what was found was an approximate length no greater than a millimeter and aspect ratio no greater than 10^5^.^[^ ^92^ ^]^ In another study, DS‐CNT fiber was lightly dispersed with super‐acids to isolate individual CNTs. They found a much smaller average length of 2 µm, although it is possible oxidative processing shorted them.^[^ ^285^ ^]^ Magneto‐resistance experiments^[^ ^329^ ^]^ and Raman analysis^[^ ^319^ ^]^ has found a characteristic distance between defects on the scale of micrometers on some DS‐CNT fibers, although this does not necessarily represent CNT length. Other studies have been able to measure CNT bundle length (an upper bound for individual CNT length) under SEM with a substrate sparsely coated from FC‐CVD. Average lengths of these graphitically high‐quality CNT bundles are 41.4±21.5 µm (Raman *G*:*D* ratio ≈47)^[^ ^326^ ^]^ and 27.4 ± 11.7 (Raman G:D ratio ≈60).^[^ ^332^ ^]^ MWCNTs in an aligned forest can be dry spun into a aligned yarn^[^ ^24^, ^333^, ^334^ ^]^ or knocked‐down into an aligned film^[^ ^307^, ^308^ ^]^ (AS‐CNTs), with much greater CNT lengths typically ranging from 200 µm to 2 mm.

The number of FWCNT walls is also an important structural metric. Among the unaligned FWCNT films, several reports indicate there is an optimum number of two to three shell walls to achieve highest conductivity.^[^ ^148^, ^149^, ^150^ ^]^ It is thought that for FWCNTs, all shells participate in the transport. This is unlike larger MWCNTs, where only the two outer shells participate, with the rest only taking up volume. In regards to aligned FWCNT material, a systematic survey did not find any substantial difference between SWCNTs and DWCNTs.^[^ ^170^ ^]^ DWCNTs, however, are easier to make and are more robust to environmental and chemical conditions than SWCNTs.^[^ ^74^ ^]^

The ends of a CNT are unavoidable defects on the CNT's rolled graphitic structure, either with open endcaps or closed hemisphere endcaps.^[^ ^335^ ^]^ Point defects also frequently appear along a CNT depending on its processing conditions. One study found 1 defect per 100 nm from CNTs made via arc vaporization and one defect per micrometer for CVD grown CNTs, with defect density increasing in regions of CNT curvature.^[^ ^69^, ^336^ ^]^ Treatment with 12% HCl acid would add on average one defect per micrometer for every hour of bathing. Another study^[^ ^337^ ^]^ found one defect every 200 to 600 nm, with an average of 350 nm. When oxidized, the average distance between defects dropped to 200 nm.

The combined end and point defect density of CNT material is typically measured by Raman spectroscopy using a well‐known metric called the G:D ratio, which is the integrated area of the *G* peak (centered at approximately 1580 cm^−1^) divided by the integrated area of the D peak (centered at approximately 1320 cm^−1^). The G peak, found in all graphitic materials, arises from in‐plane vibrations of the graphitic lattice. In the cases for CNTs, the G peak is broadened to account for vibration along and tangential to the CNT structure.^[^ ^338^ ^]^ The D peak arises from a defect‐induced break in symmetry of the graphene plane, where most of the signal originates from a 3 to 4 nm graphitic zone around the defect or grain boundary.^[^ ^339^ ^]^ Various forms of amorphous carbon also contribute to the D peak. Quantitatively, for graphite and graphene when defects are predominantly from grain boundaries, the G:D ratio is precisely linked to a characteristic crystal grain diameter *L*_a_ according to *L*_a_ ∝ (G:D)λ^4^, where λ is the wavelength of the Raman laser. For sparse networks of individual CNTs, it was shown that the same length dependence exists, where the characteristic distance equals the CNT length (*L*_a_ = *L*); there was however no simple relationship with λ because of CNTs unique helicity‐dependent 1D resonance features.^[^ ^340^ ^]^ It was found that when the CNTs are dense in a textile, sufficiently pure, and not directly in a resonant condition, that the λ^4^ response of graphite can be uncovered.^[^ ^319^ ^]^ Other studies on aligned CNT material found a linear relationship between Raman G:D ratio, CNT aspect ratio, tensile strength, and conductivity.^[^ ^170^, ^327^ ^]^ Studies on unaligned FWCNT films found at least a positive trend between conductivity and Raman G:D ratio.^[^ ^324^ ^]^ For poorly aligned FWCNT films derived from FC‐CVD, our group^[^ ^218^, ^219^ ^]^ found no positive correlation between specific conductivity and Raman G:D ratio. After a post‐process stretching further aligned the microstructure however, the correlation between Raman G:D ratio and specific conductivity became positive. An explanation is that before alignment most of the resistance came from extrinsic junctions and, after alignment, the CNTs themselves shared a significant contribution of the resistance.

Due to different excitation wavelengths, helicities, and other non‐standardized measurement procedures, it is difficult to compare quantitatively the G:D ratio for CNTs across different studies. However, as a qualitative comparison across the literature, Figure 9a shows the G:D ratio versus material properties without regard to excitation wavelength or other Raman collection parameters; correlation data are provided in **Tables** **19** and **20**. For conductivity, there is a positive Raman G:D correlation with most of the CNT categories that persists after the weighted adjustment (except unaligned MWCNT). For strength, correlation with Raman G:D correlation is largely absent. To the degree that conductivity and strength scale proportionally to CNT length (as expected for aligned CNT materials) and the degree Raman G:D ratio successfully probes length among the noise of other confounding factors, it is expected that there would be a power‐law exponent of one between Raman G:D ratio and conductivity and strength.^[^ ^170^ ^]^ For the conductivity of aligned CNT materials, the fitted slope yields a value approximately half of unity and gets closer to unity after the weighted adjustment.

**Table 19:**
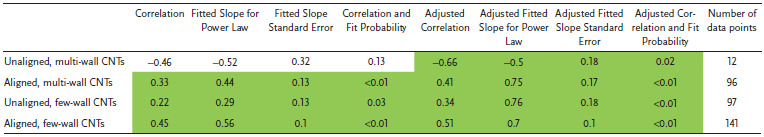
Log conductivity versus log Raman G:D ratio

**Table 20:**
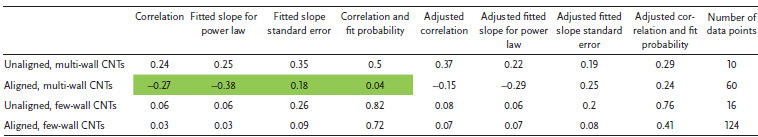
Log tensile strength versus log Raman G:D ratio

Figure 9b plots the metric G:D.λ^4^ against material properties for values in the literature; correlation data are provided in **Tables** **21** and **22**. What is expected is the same unity power‐law as for the G:D ratio, although with stronger correlations because the impact of Raman wavelength is taken into account. What is observed is, for conductivity, nearly the same correlation strengths as the standard G:D ratio across the CNT categories, if not somewhat higher for aligned FWCNT. For strength, however, we see new positive correlations for aligned FWCNT and unaligned MWCNT that persist after the weighted adjustment. This suggests G:D*.λ* ^4^ may be a more useful metric than G:D ratio (particularly for strength analysis in FWCNT fibers) or, at least, that Raman laser wavelength should be considered if comparing G:D ratios across studies. It is possible that Raman G:D ratio is beginning to lose sensitivity to measuring defects in the most graphitically pristine CNT materials;^[^ ^170^, ^278^, ^336^ ^]^ as manufacturing processes mature and conductivity improves, the defect density under the micrometer‐scale laser beam may drop below the level of practical detectability. A similar situation was found for graphitized carbon fiber, the host for the most conductive graphitic intercalation compounds, where X‐ray diffraction and Raman spectroscopy lost the ability to differentiate between highly graphitized carbon fibers.^[^ ^51^, ^341^ ^]^ Magneto‐resistance was shown to effectively measure the defect density of graphitically pristine carbon fiber^[^ ^341^ ^]^ and this approach has also been shown to be applicable to CNT conductors.^[^ ^329^ ^]^

**Table 21:**
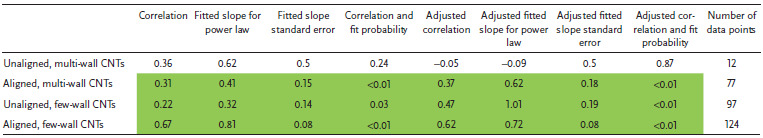
Log conductivity versus log Raman G:D ratio × λ^4^

**Table 22:**
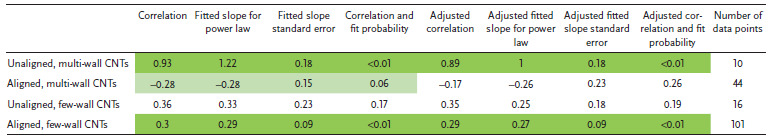
Log tensile strength versus log Raman G:D ratio × λ^4^

Structure enhancement through annealing: Post process graphitization annealing (>2000 °C in an inert atmosphere) is a standard technique for increasing the conductivity, density, and order for graphite and carbon fibers.^[^ ^89^, ^342^ ^]^ Graphitization has also been successfully applied to MWCNTs. Initially wavy, disordered walls of as‐produced MWCNTs straighten after graphitization^[^ ^343^, ^344^, ^345^, ^346^ ^]^ and increases the oxidation temperature by several hundred degrees, indicating defect removal.^[^ ^136^ ^]^ MWCNT graphitization has been shown to improve room‐temperature conductivity from 10 to 200 kS m^−1^,^[^ ^344^ ^]^ to increase thermal conductivity 2.5 to 22.3 W m^−1^ K^−1^,^[^ ^347^ ^]^ and to improve a charge carrier's mean‐free‐path from ≈0.3 to ≈2 µm.^[^ ^136^ ^]^ FWCNT graphitization however is not as straightforwardly effective where SWCNTs melt together into larger SWCNTs beginning at ≈1400 °C and by ≈1800 °C start transforming into MWCNTs.^[^ ^262^, ^263^, ^264^ ^]^ By 2400 °C it was found all CNTs transformed into MWCNTs, and in some cases become graphitic carbon ribbons.^[^ ^348^ ^]^ DWCNTs were structurally stable up to 2000 °C^[^ ^349^ ^]^ and MWCNTs structurally stable up to 2800 °C.^[^ ^350^ ^]^ The small curvature and high strain of SWCNTs, and to a lesser degree DWCNTs, make them vulnerable to oxidation, chemical treatment, and to typical graphitization annealing. The internal stresses that prevent typical graphitization treatment, however, potentially make the defects in small diameter CNTs easier to heal. First principles modeling shows that defects in the FWCNT crystal structure are not static and become mobile approaching ≈100–200 °C.^[^ ^351^ ^]^ Argon or vacuum annealing of SWCNTs well below typical graphitization temperatures has been attempted at 1000 °C^[^ ^352^ ^]^ up to 1500 °C,^[^ ^353^ ^]^ leading to an increase in graphitic perfection.

### 3.7 Intercalation Doping and Junction Enhancement

For graphitic intercalation compounds, doped conductive polymers, and CNT‐based materials, the best bulk conductivities are obtained after post‐processing the host carbon material with carefully selected chemical species. Note that here we discuss intercalation doping where the foreign chemical species, introduced after the growth process, reside adjacent to the sp^2^ carbon structure and donate charge carriers through a charge transfer process. This contrasts with any covalent bond to a defect site (functionalization) or carbon atom replacement during the growth process (substitutional doping). Substitutional doping and functionalization can tailor other physical properties, although they tend to negatively affect mobility and, ultimately, hinder the attaininment of the highest conductivities.^[^ ^70^ ^]^ Many of the intercalated dopants in graphitic intercalation compounds, conductive polymers, and CNTs are not stable in air and may degrade when exposed to ambient conditions, possible necessitating an outer sheathing structure for practical applications.^[^ ^32^ ^]^

In a graphitic intercalation compound, a chemical species is incorporated between the graphene planes of graphite in periodically ordered, stacked layers. A charge transfer process donates either holes or electrons from the intercalation layer to the adjacent graphene planes. Due to charge screening, the charge transfer from the intercalation layer rapidly decays away after the first graphene layer. This means the most conductive intercalation compounds alternate between intercalation layer to graphene layer, as opposed to less frequent regular intervals.^[^ ^79^ ^]^

Untreated single‐crystal graphite is a conductive semimetal where the conduction and valance bands overlap over a small interval (≈0.040 eV). Crystalline graphite has a high room‐temperature in‐plane mobility (13 000 cm^2^ V^−1^ s^−1^ compared to 35 cm^2^ V^−1^ s^−1^ in copper), although the carrier density is low (≈2 × 10^−4^ carriers/atom, compared to ≈2 carriers/atom in copper); this low carrier density is why the electrical conductivity of crystalline graphite is well below that of copper.^[^ ^32^, ^79^ ^]^ The graphitic intercalation compound takes advantage of graphite's high mobility and artificially increases the carrier density. Chemical species examples include electron donors such as potassium and lithium, which resulted in sub‐Kelvin superconductors. Electron acceptors include iodine, iron chloride, nitric acid, or arsenic fluoride.^[^ ^32^ ^]^ In general, the electron acceptors have led to greater conductivities. At least one paper explained that this is because the ionic radii of electron donor species, while ionized, are larger between graphene planes compared to their normal ionic radii, while ionized and without the graphene planes. The opposite is true for electron acceptor species that have smaller ionic radii, while ionized within graphene planes, compared to their normal ionic radii while ionized without graphene planes. Smaller ionic radii mean less likelihood of inadvertent chemical bonding to the host and better availability of donated charge carriers.^[^ ^34^ ^]^ In general, stronger acids with greater ionization capability lead to larger increases in hole carrier density and conductivity (as indicated roughly by the Hammett acidity function).^[^ ^34^ ^]^ In one of the highest conductivity results, using high crystallinity graphite and arsenic pentafluoride, the carrier density increases by a factor of sixty at the expense of in‐plane mobility, which is reduced by a factor of four.^[^ ^79^ ^]^ The net result is a room‐temperature conductivity beyond copper (90 MS m^−1^) and a ≈36‐fold increase in conductivity from the initial host value.^[^ ^32^ ^]^ A fundamental prerequisite for these doped conductivities is that the host graphite is of the highest crystallinity. With less than perfect crystallinity, conductivity increases of a factor of ten from the initial host value are typical.^[^ ^32^, ^79^ ^]^ Metal chlorides such as copper chloride, iron chloride, and antimony chloride has resulted in conductivities only approaching copper, although have proven to be stable in air over month long intervals.^[^ ^32^, ^354^ ^]^

Unlike graphitic intercalation compounds, the conductive carbon‐based polymers are in an insulating state before doping. The archetypical conductive polymer is doped polyacetylene, a bulk fiber composed of 1D chains of sp^2^‐bonded carbon atoms. Its initial insulating state manifests from the inherent drawback of 1D transport, the Peierls instability—a ≈1 eV bandgap formed by atomic vibrations breaking translational symmetry of a 1D conductor.^[^ ^355^ ^]^ After doping, the chemical species resides between polymer chains and ionically donates holes or electron depending on the dopant species. Again, arsenic fluoride and iodine have been shown to be the most effective dopants when the polyacetylene is particularly crystalline and aligned. Conductivity may increase more than seven orders of magnitude from the initial insulating value^[^ ^355^ ^]^ and in the best cases so far approaches copper (15^[^ ^30^, ^31^ ^]^ to 58 MS m^−1^ 100% IACS copper).

**Figure 10:**
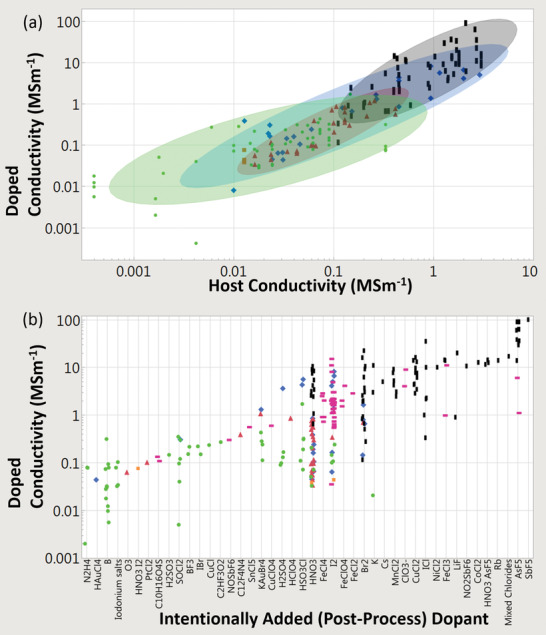
Surveyed across the experimental literature, effects of doping on CNT material. a) Post‐doped conductivity versus host conductivity for the four CNT categories. b) Post‐doped conductivities for the four CNT categories as a function of categorical dopant intentionally applied as a post‐process. Key: 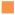) unaligned MWCNT material; 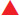) aligned MWCNT materials; 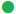) unaligned FWCNT materials; 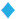) aligned FWCNT material; 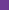) conductive polymers; 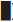) graphitic intercalation compounds. Ellipses help identify trends and are adjusted to cover 90% of the points.

In regards to CNT doping, the electron donating or accepting chemical species permeates between CNTs,^[^ ^356^ ^]^ or in the CNT cores,^[^ ^357^ ^]^ which activates the semiconducting SWCNTs (similar to activation of polyacetylene^[^ ^174^, ^356^, ^358^ ^]^) and increases the charge carrier density on metallic SWCNTs (analogous to graphitic intercalation compounds^[^ ^174^, ^356^ ^]^). Perhaps more importantly, intercalation also substantially improves charge transfer across CNT junctions.^[^ ^158^, ^358^, ^359^ ^]^ **Figure** **10**a shows the relationship between host conductivity and post‐doped conductivity for all four CNT categories; correlation data are provided in **Table** **23**. There is a significant positive correlation and a near linear relationship for most categories, which is maintained after the weighted adjustment. For graphitic intercalation compounds the host conductivity was correlated to the highest post‐doped conductivities and the trend persists in CNTs.

**Table 23:**
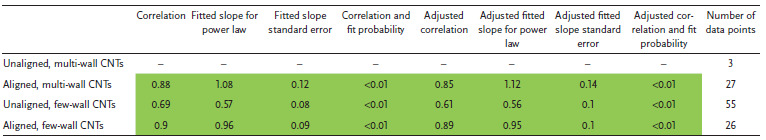
Log post‐doped conductivity versus log host conductivity

Figure 10b shows a range of intentionally added post‐process dopants and their resultant conductivities for various CNT materials. Hydrazine soaking^[^ ^77^, ^358^ ^]^ and alkali metal vapors^[^ ^359^, ^360^ ^]^ are successful n‐dopants, although unstable in air. As‐prepared CNT materials are typically exposed to ambient environmental conditions and will gradually physisorb atmospheric constituents leading to light p‐doping and a conductivity enhancement of 10–15%. Atmospherically physisorbed species can be removed by heating in vacuum to several hundred degrees Celsius.^[^ ^171^, ^172^, ^173^, ^361^ ^]^ Iodine and its derivatives are another prevalent CNT p‐dopant that can increase the conductivity of aligned CNT fiber two to seven times,^[^ ^13^, ^14^, ^15^, ^299^ ^]^ leading to several record conductivities.^[^ ^14^, ^15^ ^]^ For less aligned FWCNT material, iodine and its derivatives can increase the conductivity six to ten times.^[^ ^279^, ^362^, ^363^, ^364^, ^365^, ^366^, ^367^ ^]^ Thionyl chloride is another effective p‐dopant for CNT materials leading to a conductivity enhancement by a factor of 3 to 13 times.^[^ ^13^, ^362^, ^365^, ^368^, ^369^ ^]^

Soaking in nitric, sulfuric, or chlorosulfonic acids are other typical deliberate p‐doping procedures, increasing the conductivity up to a factor of ten for aligned CNT materials^[^ ^14^, ^183^, ^188^, ^211^, ^237^, ^273^, ^280^, ^298^, ^370^, ^371^ ^]^ (one case, up to 30^[^ ^189^ ^]^) and up to 28 for unaligned CNT materials^[^ ^150^, ^158^, ^166^, ^179^, ^329^, ^362^, ^363^, ^372^ ^]^ (in one case with controlled helicity, up to 100^[^ ^358^ ^]^). The conductivity enhancement of these acids in some cases is partly from an increase in carrier density with a relative down shift of the Fermi level ranging anywhere from 0.35 to 0.50^[^ ^372^ ^]^ to nearly 1 eV.^[^ ^358^ ^]^ Chlorosulfonic acid was shown to shift the Fermi level by 0.7 eV for double‐wall CNTs 1.9 in diameter, resulting in a factor of five increase in conductivity.^[^ ^174^ ^]^ Alternatively, conductive atomic force microscopy studies show that almost all the conductivity enhancement from acid stems from improved transmission across SWCNT junctions, where conductivity of the SWCNT structures themselves are largely unaffected.^[^ ^158^, ^166^ ^]^ Acids may also remove amorphous carbon, catalyst, surfactant, and other impurities, although some acids, such as nitric acid, can degrade CNT crystallinity^[^ ^373^ ^]^ (particular with small diameters^[^ ^13^ ^]^). Very strong acids, such as chlorosulfonic acid, in addition to doping effects, also separates bundled CNTs through protonation. This makes the bundle diameters smaller and, alongside doping, increases conductivity.^[^ ^150^, ^179^ ^]^

When an unaligned film composed of predominantly metallic SWCNTs was p‐doped with nitric acid, the conductivity increased a factor of four.^[^ ^358^ ^]^ This was attributed to improved junction transport because carrier density enhancement was not observed. Chemically enhancing carrier density of metallic SWCNTs has otherwise been demonstrated^[^ ^190^, ^356^ ^]^ and is analogous to the graphitic intercalation compounds.^[^ ^374^ ^]^ A nitric acid doped, predominantly semiconducting SWCNT film however had a profound conductivity increase of nearly a hundred, indicating doping activated the semiconducting SWCNTs.^[^ ^358^ ^]^ Doped semiconducting SWCNT films are often more conductive than metallic SWCNT films, doped or otherwise. As discussed earlier in the intrinsic transport section, the superior conductivity of doped semiconducting SWCNT films to metallic SWCNT films is counter intuitive and has been attributed to more effective carrier density enhancement on the CNTs and better charge transfer across semiconducting SWCNT junctions.^[^ ^63^, ^77^, ^358^, ^375^ ^]^

## 4 Discussion and Summary

### 4.1 Partitioning of Intrinsic and Extrinsic Characteristics in a CNT Wire

**Figure 11:**
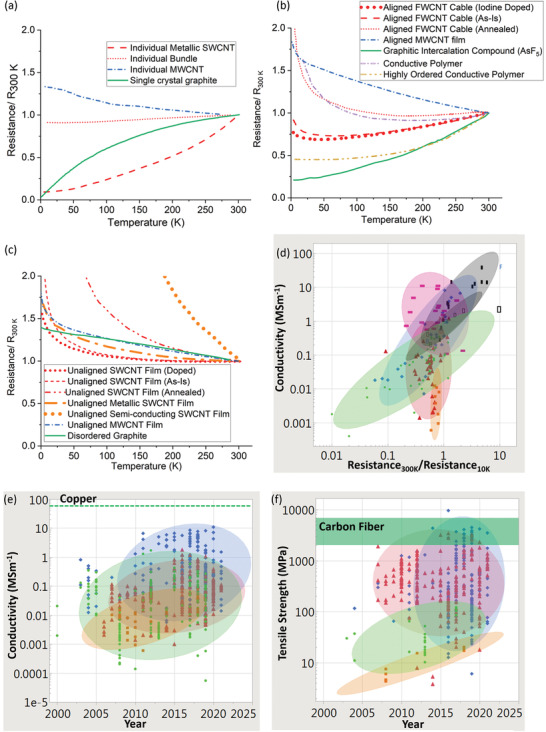
a–c) Resistance versus temperature traces for specific studies for intrinsic, individual elements (a),^[^ ^79^, ^103^, ^142^, ^154^ ^]^ carbon‐based materials with high order and microstructure alignment (b),^[^ ^15^, ^79^, ^378^, ^381^ ^]^ and carbon‐based materials with low order and no microstructure alignment (c).^[^ ^172^, ^309^, ^379^ ^]^ d) Plot of conductivity versus ratio of resistance at 300 K divided by resistance at 10 K. e,f) Plots showing incremental conductivity and strength improvement over the years since the early 2000s. Key: 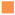) unaligned MWCNT material; 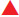) aligned MWCNT materials; 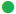) unaligned FWCNT materials; 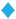) aligned FWCNT materials. 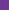) conductive polymers; 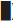) graphitic intercalation compounds; 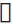) carbon fiber and graphite. The ellipses help identify trends and are adjusted to cover 90% of the points.

In this meta‐analysis, we surveyed the intrinsic and extrinsic properties of a CNT cable; as manufacturing processes continue to improve, we would expect that most of the CNT fiber resistance will originate from the intrinsic contribution (CNTs and bundles) and less from the extrinsic (voids, impurities, junctions). Note that our usage of “intrinsic transport” does not designate the doping state (as in traditional semiconductor parlance); it refers to the limiting transport if a bulk CNT material could be manufactured with perfect alignment, maximum density, zero impurities, and no junctions between CNT structures. The limiting transport is dictated by the individual CNT or, as argued here, possibly the CNT bundle. In this sense, the intrinsic conductivity can be enhanced by increasing the mean‐free‐path (decreasing defect density), increasing carrier density (by doping), and possibly making smaller‐diameter bundles (If, indeed, the full bundle cross‐section is not used). **Figure** **11**a shows the resistance versus temperature response of some specific cases of these intrinsic elements. Single crystal graphite,^[^ ^79^, ^342^ ^]^ an individual metallic SWCNT,^[^ ^103^ ^]^ and a CNT bundle^[^ ^154^, ^376^ ^]^ have a completely metal‐like increase in resistance with increasing temperature over the entire temperature range, typically from phonon interaction.^[^ ^355^, ^377^ ^]^ As intrinsic elements are assembled into a larger agglomeration, the resistance verses temperature response changes. Many FWCNT films of unaligned microstructure,^[^ ^309^ ^]^ MWCNT materials,^[^ ^378^ ^]^ individual MWCNTs,^[^ ^139^ ^]^ and disordered graphite^[^ ^379^ ^]^ have a completely semiconducting resistance response, from room‐temperature to cryogenic temperature (Figure 11c). This also includes unaligned films of predominantly metallic SWCNTs;^[^ ^172^, ^329^ ^]^ though the CNTs individually are metallic, this does not mean the bulk material will have the standard metal‐like increase in resistance with increasing temperature. Alternatively, more ordered materials, such as CNT cables with aligned microstructure,^[^ ^15^ ^]^ less disordered carbon fibers,^[^ ^320^ ^]^ and conductive polymers,^[^ ^380^, ^381^ ^]^ have a parabolic‐like resistance versus temperature response (Figure 11b). Here, there is a metal‐like response at higher temperatures and a semiconductor‐like response at lower temperatures with a minimum in resistance at the crossover. Figure 11d shows the conductivity versus the ratio of resistance at 300 K divided by the resistance at 10 K for material categories in this meta‐analysis; correlation data are provided in **Table** **24**. For the FWCNT materials, over a large range of conductivities spanning nearly 0.01 to 10 MS m^−1^, there is significant positive correlation that is maintained after the weighted adjustment. For MWCNT materials, a consistent correlation is largely absent. These correlations in FWCNT materials demonstrate the importance of the metallic‐like temperature response over the semiconducting‐like temperature response, without adopting any specific transport mechanisms.

**Table 24:**
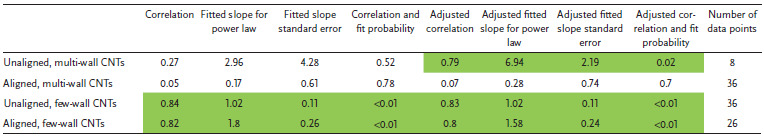
Log conductivity versus log *R*_300K_/*R*_10K_

Details of popular transport models are discussed elsewhere,^[^ ^68^, ^376^ ^]^ although to better understand the meaning of Figure 11d, we briefly address the origins of the semiconductor‐like resistance‐temperature response as intrinsic CNT elements are brought together into an assembly. For disordered CNT materials, where the resistance exponentially diverges as absolute zero is approached,^[^ ^309^, ^329^, ^382^, ^383^, ^384^ ^]^ there is no difficulty in assigning this to Mott variable range hopping. Here, the intrinsic CNT conductivity is less important, junctions between CNTs dominate the transport, and localized charge carriers hop (or tunnel with a phonon exchange) to a distant localized state of similar energy level. For better ordered CNT materials, resistance still increases with decreasing temperature, although approaching absolute zero, now converges to a finite value or increases according to a power‐law; various transport mechanisms lead to this more modest resistance increase and are somewhat inconsistently utilized in the literature. Weak localization is one such mechanism, where cryogenic temperatures freeze out phonon‐interactions and enable the charge‐carrier back‐scattering from crystal defects to constructively add and oppose the transport. Weak localization was first applied to the anomalous resistance increase when cooling thin film metals^[^ ^385^, ^386^ ^]^ and was next used to explain the resistance dependence on magnetic field is disordered graphite.^[^ ^379^, ^387^, ^388^ ^]^ Later, weak localization explained the cryogenic magnetic field dependence of resistance for a variety of conductive polymers,^[^ ^389^, ^390^ ^]^ individual MWCNTs,^[^ ^391^ ^]^ individual CNT bundles,^[^ ^392^, ^393^ ^]^ unaligned CNT materials,^[^ ^172^, ^309^, ^394^ ^]^ aligned MWCNT^[^ ^378^ ^]^ and FWCNT^[^ ^309^, ^395^, ^396^ ^]^ materials. Some of these studies^[^ ^378^, ^389^, ^390^, ^395^ ^]^ also incorporated another transport mechanism, where electron–electron interaction (EEI) reduces the thermal electron diffusion through states near the Fermi energy, although was only applicable for temperatures less than 4 K. Generally, these studies demonstrated that weak localization and EEI were a small addition to the resistance, well within 5% of the unmodified resistance. While successful in describing magnetic field dependence, these listed studies did not quantitatively address, at least in detail, the significant^[^ ^158^, ^166^, ^167^ ^]^ influence of resistive junctions between CNT structures on the bulk transport. It is unlikely that the small resistance contribution from weak localization and EEI, nor the too large contribution from hopping,^[^ ^396^ ^]^ can explain the full resistance‐temperature dependence of ordered CNT materials, such as those shown in Figure 11b or other CNT transport studies with aligned microstructure^[^ ^329^, ^397^, ^398^ ^]^ (particularly when doping influence has been removed^[^ ^15^ ^]^).

A heterogeneous transport model, on the other hand, as applied to conductive polymers^[^ ^399^ ^]^ and CNTs,^[^ ^15^, ^68^, ^278^, ^307^, ^329^, ^359^, ^376^, ^377^, ^380^ ^]^ considers a scenario where anisotropic conductive filaments are separated by thin semiconducting barriers to conduction; resistance here is modeled by two terms that add in series: 1) some metallic‐like term (where resistance increases with increasing temperature) and 2) a semiconducting‐like fluctuation‐induced tunneling term (where resistance increases with decreasing temperature, although eventually saturates approaching absolute zero). The fluctuation‐induced tunneling models the contribution from CNT junctions and other extrinsic effects. This parabolic‐like response with crossover resistance minimum (Figure 11b) is a signature of this heterogeneous transport. It has been demonstrated that no temperature‐induced phase transitions or qualitative changes in transport occur at the crossover resistance minimum; therefore, the competition of two transport mechanisms—metallic and semiconducting resistance terms adding in series—is the best explanation of the parabolic‐like resistance response with temperature.^[^ ^68^, ^376^ ^]^ For these reasons, the semiconductor‐like increase in resistance with decreasing temperature is primarily from the extrinsic effects of junctions and voids (as modeled with fluctuation‐induced tunneling or hopping) with at most a marginal contribution from weak localization and EEI. Therefore, the variation of conductivity versus room‐temperature to cryogenic resistance ratio (Figure 11d) is primarily a measure of the intrinsic and extrinsic contributions of the CNT material; the greater this resistance ratio, the greater the influence of the intrinsic transport on the overall room‐temperature conductivity. Note that fluctuation‐induced tunneling was not considered in many transport studies^[^ ^172^, ^309^, ^378^, ^389^, ^390^, ^394^, ^395^, ^396^ ^]^ involving weak localization; further there has been some criticism in that fluctuation‐induced tunneling has not adequately fit some datasets or is ambiguous in respect to other possible transport mechanisms such as hopping. In response, other transport mechanisms involving Landauer formalism^[^ ^398^ ^]^ and Luttinger liquid theory^[^ ^397^ ^]^ have been recently proposed and will need to be evaluated by the community, hopefully using sufficiently cold temperatures (<4 K) where differences become more apparent.

For studies that implemented the heterogeneous model and fluctuation‐induced tunneling, we now discuss the exact partitioning of the intrinsic and extrinsic transport from these specific CNT studies, using the specific transport models and fitting parameters provided by the respective authors. In general the following studies use the same fluctuation‐induced tunneling term, while the functional form for the metallic‐like term was varied. The extrinsic percentage contribution to room temperature resistance is simply calculated by taking the fluctuation‐induced tunneling term, dividing it by the sum of all model terms, and evaluating at room temperature. For tangled SWCNT rope, the extrinsic room‐temperature contribution is 95% of the total resistance.^[^ ^377^ ^]^ For FWCNT material from FC‐CVD^[^ ^278^ ^]^ (DS‐CNT), after microstructure alignment and removal of impurities and less conductive CNTs, the extrinsic contribution is 82%. For one of the highest conductivity aligned SWCNT fibers from acid extrusion (SS‐CNT), the extrinsic room‐temperature contribution to resistance was 57%. After iodine doping and further conductivity enhancement, the extrinsic contribution was 63%.^[^ ^15^ ^]^ For comparison, in doped polyacetylene, the extrinsic contribution at room‐temperature ranged from 87% to 100% of the total resistance.^[^ ^399^ ^]^ There are cases of highly ordered conductive polymer^[^ ^381^, ^400^, ^401^ ^]^ and graphitic intercalation compounds^[^ ^79^ ^]^ having a complete metallic‐like resistance versus temperature response (Figure 11b); proof in principal of the possibility to pacify the extrinsic contribution with superior processing, although this has yet to be demonstrated in CNT materials.

The accumulation of literature data for aligned MWCNT material has shown the opposite to the expected trends between alignment and conductivity and strength (in respect to FWHM from X‐ray diffraction and Raman anisotropy). There was no conductivity correlation with room‐temperature/cryogenic resistance ratio; further, there was lack of convincing enhancement of specific properties as density is increased. Taken together across a wide variety of sources, it is apparent that these are not spurious correlations and that more fundamental research on MWCNT material is required. A possible explanation worth exploring is that greater microstructure alignment and less voids generally leads to a greater contribution from the intrinsic CNTs over larger‐scale extrinsic factors. MWCNTs on an individual molecular level never had an intrinsic metal‐like resistance increase with increasing temperature (Figure 11a); further, there was no correlation between conductivity and room‐temperature/ cryogenic resistance ratio (Figure 11d). For aligned MWCNT yarn spun from forests (AS‐CNTs), one study^[^ ^402^ ^]^ showed that the CNTs themselves limited the room‐temperature conductivity and that junction resistance only became significant at cryogenic temperatures. It is possible the intrinsic properties of MWCNT have not successfully manifested on an individual or bulk level, leading to the unexpected trends on larger scales. Considering that single‐crystal graphite and graphitized carbon fiber can have a fully metal‐like resistance response with temperature (Figure 11a,b), this should still be possible for individual MWCNTs provided the defect density is lowered.

### 4.2 Leading Properties of the Ultimate CNT Cable

Yarns made from spinning MWCNT forests (AS‐CNTs) have a competitive degree of microstructure alignment, long CNT length, and purity compared to aligned FWCNT material, although in terms of strength they are somewhat lower than aligned FWCNT material and, in terms of electrical conductivity, they are significantly lower. The distinction between MWCNT material and FWCNT material is thus one of the most important classifications to be considered first before addressing other material properties. Among the aligned FWCNT material, there is an emerging class of aligned bucky papers with near perfect microstructure alignment, packing density, purity, and even control of the electronic CNT species (AF‐CNTs). Their conductivity is relatively low (0.25–0.4 MS m^−1^) because the CNT length is relatively low (<500 nm).^[^ ^9^, ^188^, ^189^, ^190^ ^]^ For the most conductive CNT fiber, the average CNT length is 12 µm.^[^ ^29^ ^]^ Longer CNT length and smaller diameter (or higher aspect ratio) is likely the primary governing property for fundamental enhancement of strength and conductivity in aligned FWCNT materials. We argued that bundles are the intrinsic building block in CNT cables, opposed to individual CNTs, and are analogous to single‐crystal graphitic crystallites in graphite. Large bundle diameters should also be another limiting factor in aligned FWCNT material transport, although this has not been demonstrated conclusively in experiment on aligned FWCNT materials. Increasing density intrinsically increases the conductivity of aligned FWCNT materials; for other CNT categories and tensile strength, however, increasing density improves material properties trivially from simply more material present. Doping will be required to bring conductivity substantially beyond single‐crystal graphite and to conductivities approaching graphitic intercalation compounds and conductive polymers.

Note that in the aggregation of data, conductivity of unaligned FWCNT material never strongly benefited from sorting between metallic or semiconducting electronic species (Figure 3a). For the bucky paper with aligned microstructure (AF‐CNT), unsorted still has the highest conductivity^[^ ^9^ ^]^ compared to the conductivities with sorted FWCNT concentrations. Finally, acid extrusion of metallic or semiconducting FWCNTs into aligned fibers (SS‐CNTs), at least in one case,^[^ ^403^ ^]^ never led to major conductivity improvement over unsorted fibers. This is possibly a result of damage from processing conditions and more work is required, although there is a body of evidence showing that control of the electronic species is not a top priority.

### 4.3 Analogy between CNTs, Graphene, and Single‐Crystal Graphite

The hierarchy between graphene, single‐crystal graphite crystallites, and graphite is analogous to the hierarchy between CNTs, CNT bundles, and CNT cables. Graphene optimally assembled into single‐crystal graphite with perfect ABAB stacking reaches a conductivity of only 2.5 MS m^−1^,^[^ ^161^, ^162^ ^]^ approximately the value of perfectly packed and aligned SWCNT bundles and the best undoped aligned FWCNT fibers. This graphite however transforms into the most highly conductive (at room temperature) macro‐substance known after intercalation doping, where every graphitic plane is separated by an electron accepting intercalation species.^[^ ^79^ ^]^ Thus, in this meta‐analysis we in part covered the intrinsic properties of CNT and graphene to understand: 1) how an assembly of individual CNTs into a CNT bundle and then into a CNT cable compares to an assembly of graphene into a single‐crystal crystallite and into carbon fiber and 2) in what ways could a doped CNT cable compete with a graphitic intercalation compound. The following are a summary of the compiled observations between graphene and CNTs: 1) The experimentally measured mean‐free‐path for graphene and metallic CNTs are similar from room‐temperature to cryogenic temperature.^[^ ^404^, ^405^, ^406^ ^]^ Undoped, in their larger assemblies of single‐crystal crystallites and CNT bundles, they have approximately the same conductivity. This means that superior conductivity in either assembly will be from greater carrier density. Activated semiconducting SWCNTs at room‐temperature can have mean‐free‐paths approaching metallic SWCNTs and graphene. 2) By virtue of being conductors with restricted transport dimension, both graphene and CNTs have spikes in the density of states called van Hove singularities. Doping to the van Hove singularity is a means to dramatically increase the carrier density. The van Hove singularities are fixed and relatively far away in graphene; in CNTs their position relative to the Fermi is dependent on the CNT diameter, as well as the specific electronic species. Semiconducting CNTs generally have the closest van Hove singularity to the Fermi level for a given diameter. 3) CNT and their bundles are inherently bendable. Single‐crystal graphite, at its largest on order of square millimeters, is brittle and must be handled with special tweezers to avoid damaging the ABAB stacking.^[^ ^42^, ^161^, ^162^, ^342^ ^]^ Graphitized carbon fiber is similarly mechanically impractical and not conducive for handling after doping. For these reasons, we see CNT cables being fundamentally superior to single‐crystal graphite and graphitized carbon fiber as a high‐mobility host for a doping chemical species. Although recent experimental evidence deemphasizes the importance of electronic species control, for the theoretical reasons above, we still expect that a sufficiently doped semiconducting SWCNTs will be superior to metallic SWCNTs, doped or not, in a CNT cable with aligned microstructure.

### 4.4 CNT Metal Composites

While this meta‐analysis primarily focuses on pure CNT fibers with possibly doping and cross‐linking, we briefly mention CNT–metal composites and their attributes here; an in‐depth analysis can be found elsewhere.^[^ ^23^, ^62^ ^]^ The best CNT–metal composites are associated with a high CNT volume fraction (greater than 20%) and thorough, intimate mixing at the molecular level.^[^ ^23^, ^221^ ^]^ With these favorable conditions, the mechanical strength of the composite is found to increase two to four times compared to the host metal at the expense of electrical conductivity.^[^ ^222^ ^]^ One of the best strength‐enhanced CNT–copper composites,^[^ ^220^ ^]^ for example, had a tensile strength of 800 MPa, as compared to 300 to 400 MPa for unalloyed copper, although the conductivity (20 MS m^−1^) was one‐third that of copper. Only recently has the strength been increased (doubled for an aluminum matrix), with no reduction in conductivity after CNT incorporation.^[^ ^222^ ^]^ Inherent improvement of electrical transport on the other hand, as opposed to trivial enhancement of conductivity by simply adding copper, has only been rarely reported. In one case,^[^ ^242^ ^]^ a CNT–copper composite had an ampacity of 10^12^ A m^−2^, over an electromigration‐limited pure copper value of 10^10^ A m^−2^. This was for substrate‐bound strips 50 µm long (≈1 µm tall and wide); in larger, free‐hanging CNT–copper composite cables, the vacuum‐measured ampacity dropped to ≈10^8^ to 10^9^ A m^−2^,^[^ ^220^, ^265^ ^]^ which is back within range of conventional metals and pure CNT materials. Note that, in the case of the high‐ampacity substrate‐bound strips versus the lower ampacity cables, these were measured with probe separation of ≈50 µm and 5 mm respectively; in the ampacity discussion (Figure 6) we showed the literature‐wide dependence of ampacity on diameter and the influence of probe‐separation and have not seen evidence for the ultrahigh 10^12^ A m^−2^ ampacities scaling to macroscopic samples; further, this particular study was an outlier in the overall body of CNT ampacity literature. Some CNT–copper composite studies have found a specific electrical conductivity approaching that of copper.^[^ ^220^ ^]^ It should be remembered, however, that adding copper to anything will make the conductivity and specific conductivity closer to those of copper; to be worthwhile, intrinsic enhancement must be demonstrated. Only recently^[^ ^221^ ^]^ has a synergistic outcome occurred, yielding a large‐scale CNT–copper composite with specific conductivity (9.4 kS m^2^ kg^−1^) nearly 50% greater than copper (6.7 kS m^2^ kg^−1^). While aluminum (13 kS m^2^ kg^−1^) is a more appropriate benchmark for specific conductivity, this demonstrates that the CNT–metal composites may not be a zero‐sum game of property trade‐offs. Pure, though possibly crosslinked or doped CNT cables, are already substantially stronger than CNT–metal composites and currently have similar stratified multifunctional (strength x conductivity) values; in terms of intrinsic conductivity, pure CNT cables should be superior. Nevertheless, to the degree that the developments of^[^ ^221^ ^]^ can be improved upon, this may be one possible route to sidestep extrinsic restrictions in CNT cables for electrical power applications.

## 5 Conclusion

Figure 11e,f shows the steady progress in the conductivity and strength of CNT materials over the years, since they were first routinely assembled into a macroscopic aligned assembly in the early 2000s. Aligned FWCNT materials have had the most significant growth rate in terms of conductivity and strength. For acid‐extruded FWCNT fibers (SS‐CNTs), it has been observed that material properties approximately double every three years,^[^ ^29^ ^]^ based on the individual FWCNT length. With respect to conductivity, considerable progress is still required for any CNT material to reach the established benchmarks: the conductivity of copper in absolute terms or the conductivity of aluminium in weight specific terms. Graphitic intercalation compounds have already demonstrated this to varying degrees of success and stability; we have argued that CNTs should in principle be better than graphitic intercalation compounds, although this has yet to be demonstrated in a lab in terms of maximum conductivity. The information in this meta‐analysis is in part meant as a roadmap through the literature for this objective. In terms of strength however, CNT cables have been on‐par with existing benchmarks materials to include synthetic fibers and carbon fiber. In terms of specific strength, CNT cables continuously extracted from FC‐CVD reactors (DS‐CNT) currently exceed these benchmarks. Properties will continue to climb with greater CNT length and better processing, relative to synthetic fibers and carbon fiber that have been relatively stagnant. In terms of the multi‐functional metric (product of strength and conductivity), CNT materials are by a large margin the leading material. In this way we see CNT cables as the next generation fiber, currently competitive with benchmark materials in terms of strength and multifunctionality—and with continued development, electrical conductivity.

The future direction of CNT materials research will use statistical methods^[^ ^218^, ^219^ ^]^ and closed‐loop, artificial‐intelligent automation^[^ ^407^, ^408^, ^409^, ^410^, ^411^ ^]^ to efficiently explore vast, multi‐dimensional, and confounded experimental spaces formed by CNT production and processing systems. In addition to simply reporting material property results, there is utility in reporting metrics indicating statistical model performance (indicating repeatability and degree of system understanding) and provide the experimental databases that generate the models. FWCNTs will be developed with greater efficiency and yields at lower costs, with ever‐increasing length, less defects, smaller diameter, and control of helicity– likely with FC‐CVD‐like systems due to their demonstrated relatively large yields of high‐quality CNTs.^[^ ^21^, ^66^ ^]^ High‐quality CNTs are sold at $2000 to $100 000 USD per kilogram with an approximately 100 tons per year production capacity (as of 2019), and these commercial metrics have been rapidly improving orders of magnitude over the last five years.^[^ ^29^ ^]^ Carbon and synthetic fibers, for comparison, are at $10 to $200 USD per kilogram and are produced at approximately 100 000 tons per year. With higher quality CNTs more available, bulk material properties will correspondingly improve together with compounding academic and commercial impact. Like conventional carbon fiber, the strongest CNT materials will additionally incorporate cross‐linking mechanisms holding the CNTs together; this will come at some expense of conductivity. The very highest conductivity CNT materials will be doped, debundled and possibly completely isolated from each other, coming at the cost of strength. Other remaining extrinsic limitations may be overcome by targeted doping or Ohmic cross‐linking. Made from sustainable technologies^[^ ^412^ ^]^ that captures more carbon than it releases, we ultimately see a multifunctional cable more conductive than copper and much stronger than carbon fiber. The emerging field of 1D heterostructures (or hybrid nanotubes) factorially expands the already vast experimental space formed by CNTs.^[^ ^413^, ^414^ ^]^ This can increase the multifunctional capabilities of nanotube‐based materials, such as possibly providing a protective sheath around a CNT core and increase the operating temperature of a wire. More interestingly, as 2D heterostructures have demonstrated, interaction between different tubular layers will have correlated phenomena such as tailorable, ultrahigh mobility^[^ ^415^, ^416^ ^]^ and switchable superconductivity^[^ ^417^ ^]^—all in a morphology suitable for a flexible wire. Layer interaction of hybrid nanotubes; CNTs’ diameter‐dependent, strong electron‐phonon coupling; and the potential of CNT's to dope into the van Hove singularities are routes for superconductivity^[^ ^81^, ^154^ ^]^—phenomena already demonstrated in other 1D molecules,^[^ ^418^, ^419^ ^]^ other doped carbon allotropes^[^ ^81^ ^]^ and some CNTs architectures.^[^ ^420^ ^]^ Superconducting nanotube‐based wires could be mechanically strong and bendable, which are features difficult to achieve with current superconductors and are some of the primary limitations to powerful electromagnets. After many years of development and publicity, starting with graphitic intercalation compounds and conductive polymers, CNT based materials have now achieved parity with real‐world engineering materials and there is a clear, scientifically compelling path for CNTs to far exceeding them.

## 6 Experimental Section

The material property database of CNT materials, other advanced carbon‐based conductors, and benchmark materials collected widely across the literature is provided in the Supporting Information section with references indicated. In the life sciences, a meta‐analysis is a widely used methodology, comprising a formalized study with procedures outlined here,^[^ ^421^, ^422^ ^]^ while in the field of carbon nanotubes only one meta‐analysis is known, and it was concerning FC‐CVD.^[^ ^66^ ^]^ Similar to these previous meta‐analyses, the scope of materials covered the rationale for paper selection and statistical methods employed. Pure CNT materials, doped CNT materials, and cross‐linked CNT materials were considered, without including CNT materials mixed with significant composite or metal matrix or additional binder. The exception is for the ampacity discussion and conclusion, where metalized CNTs were briefly discussed. CNT materials included in the database were free‐standing and thick enough to be beyond any percolation threshold effects. Studies that were incorporated into the analysis had to at least offer some amount of data on conductivity and strength; other data to include Raman spectroscopy, alignment, density, and transport data was additionally included into the database.

CNT materials were placed in four categories: unaligned MWCNT, aligned MWCNT, unaligned FWCNT, and aligned FWCNT. It was typically straightforward to determine the alignment category, based on known processing conditions or specific alignment data. CNTs extracted continuously from floating catalyst chemical vapor deposition (DS‐CNTs) are always put in the aligned category, although it is understood that it is possible this alignment could be very low under certain FC‐CVD conditions. Determining the MWCNT/ FWCNT category was sometimes more ambiguous. Having radial breathing modes or diameters less than 3 nm were always assigned as FWCNTs; having three or fewer average walls were typically assigned as FWCNT. Often there was assignment ambiguity associated when a paper reported a spread of diameters and wall numbers, although the spread tended to be weighted more toward MWCNT and were assigned as such. When a reported range of wall numbers extended past four, it was assigned as a MWCNT.^[^ ^24^, ^294^, ^300^, ^423^ ^]^ In a few cases, there were large diameter (7–8 nm) double‐wall CNTs and these were assigned as FWCNTs;^[^ ^424^ ^]^ in another case the double‐wall CNTs were too large in diameter (7–14 nm) and, because quasi‐1D transport was no longer suspected over approximately 10 nm, it was assigned as a MWCNT.^[^ ^237^, ^425^, ^426^ ^]^ In other words, regardless of wall number, when the outer CNT diameter extended past 10 nm, it was assigned as a MWCNT. When no determination could be made, they were assigned to be MWCNTs.^[^ ^260^, ^280^, ^299^, ^316^, ^317^, ^427^ ^]^ CNTs were also assigned a doped/undoped category. Intentional doping is straightforwardly identified in a paper. If there is any acid processing used to purify or make the CNT fibers, this material too would be assigned as doped. Neutralization in solvent or water does not count as de‐doping; this is based on the experience of vacuum baking substantially reducing the conductivity in these neutralized, acid processed materials. Only specific processes to evacuate the remaining acid constituents, typically the high temperate vacuum bake, would assign these acid processed materials as undoped.

Typically, information on a paper incorporated into the database was taken as is, without pulling in additional information from other papers or making any extra assumptions. However, there were some exceptions to this. To determine specific properties of intrinsic elements such as individual FWCNTs and bundles, for the purposes of comparison, a standard procedure had to be applied for estimating density. The density of individual FWCNTs was estimated using Figure 7a provided by Laurent et al.^[^ ^266^ ^]^ Individual MWCNTs were estimated to be 2.1 g cm^−3^ for diameters ranging from 8 to 50 nm.^[^ ^133^ ^]^ Individual FWCNT bundles were estimated to be 1.1 g cm^−3^ for FWCNT diameters ranging from 0.815 to 1.4 nm.^[^ ^133^, ^269^, ^281^ ^]^ Frequently in CNT reports, CNT diameters are given as a range of values; for the purposes of the meta‐analysis the range is reduced to the midpoint. The stratified conductivity values of individual FWCNTs were taken by considering the master curve of 6 kΩ µm^−1^ for experimentally measured ultralong FWCNTs in the diffuse limit, for both a FWCNT with 1 nm diameter and another with 2 nm. Other individual FWCNT, bundle, and MWCNT conductivities of lower values came from specific experiments. While many reports on strength, electrical, and thermal conductivity of individual CNT structures use an annulus area to calculate cross section, in this report these values were modified to account for the entire circular cross section of the CNT to obtain lower “engineering” values.

Plots depicted here were typically logarithmic scales due to the great spread of values typical for CNT materials and in the interests of identifying power‐laws. Colored ovals are sized to encompass 90% of the data points and help identify trends. In the discussion of ampacity, ovals indicated 95% confidence intervals. On the logarithmic data, for a given CNT category, the strength of the Pearson correlation coefficient and the probability (*p*‐value) that it is significant were calculated . Probabilities less than or equal to 0.05 (*p* ≤ 0.05) are deemed significant and colored green; probabilities between 0.1 and 0.05 (0.05 ≤ *p* ≤ 0.1) are deemed possibly significant and colored gray. The fitted slope and standard error of the logarithmic data set were also calculated; this slope is the exponent of the power‐law. Different studies offer different amounts of data points, opening the possibility some studies could unduly skew the aggregated correlations by simply offering more points. To guard against undue bias from specific studies, correlation strength, power‐law fit, and their probability (*p*‐value) with a weighting function were also calculated . The weight is assigned to give each paper, within a material category and within the properties in question, equal impact to the correlation and fit. Correlations and fits that remain statistically significant and do not change much between unweighted and weighted calculation imply that the trends apply across the literature, opposed to a few specific studies. If the correlation loses its significance before or after the weighted adjustment, the trend may still be useful although its applicability across the literature is taken less assuredly. The weighting function was calculated as follows. For a given CNT category, and for the two material properties in question, the number of data points offered in each contributing paper *n* was determined and the reciprocal calculated, *n* ^−1^. The weight assigned to each data point is then *n* ^−1^ divided by the sum of every *n* ^−1^ across all data points being considered. This ensures that the sum of the weights is unity and that the sum of the weights within each paper is equal. In some cases when comparing categorical data t‐tests were used; this analysis is in the Supporting Information section. Box plots were also used in the main text to help compare categorical data, particularly to visually show differences from doping. The t‐test and box plots should be considered in the context that the material categories, which may have fundamental differences, are in continual development across multiple research groups with varying degrees of quality. Considering the larger picture of averages, standard deviations, maximum values, and paths to improvement are necessary when evaluating material categories.

## Conflict of Interest

The authors declare no conflict of interest.

## Supporting information

Supporting Information

Supporting Information
