# Supplementary material for: A Meta‐Analysis of Conductive and Strong Carbon Nanotube Materials
Source: Adv Mater. 2021 Jul 19;33(36):2008432. doi: 10.1002/adma.202008432 (PMC11469326; doi:10.1002/adma.202008432)
Supplement: Supplementary file 1 — Supporting Information [file ADMA-33-2008432-s002.pdf]

© 2021 Wiley-VCH GmbH

ADVANCED MATERIALS

Supporting Information

for *Adv. Mater.,* DOI: 10.1002/adma.202008432

A Meta-Analysis of Conductive and Strong Carbon Nanotube Materials

John S. Bulmer, Adarsh Kaniyoor, and James A. Elliott*

Supplemental Section for

A Meta-analysis of Conductive and Strong Carbon Nanotube Materials

John Bulmer, Adarsh Kaniyoor, James Elliott

Department of Materials Science and Metallurgy, 27 Charles Babbage Road, University of Cambridge, CB3 0FS, UK

Below are various t-tests used to statistically compare properties of two CNT categories.

ANOVA and t-test comparing strengths of aligned MWCNT material versus aligned FWCNT material.

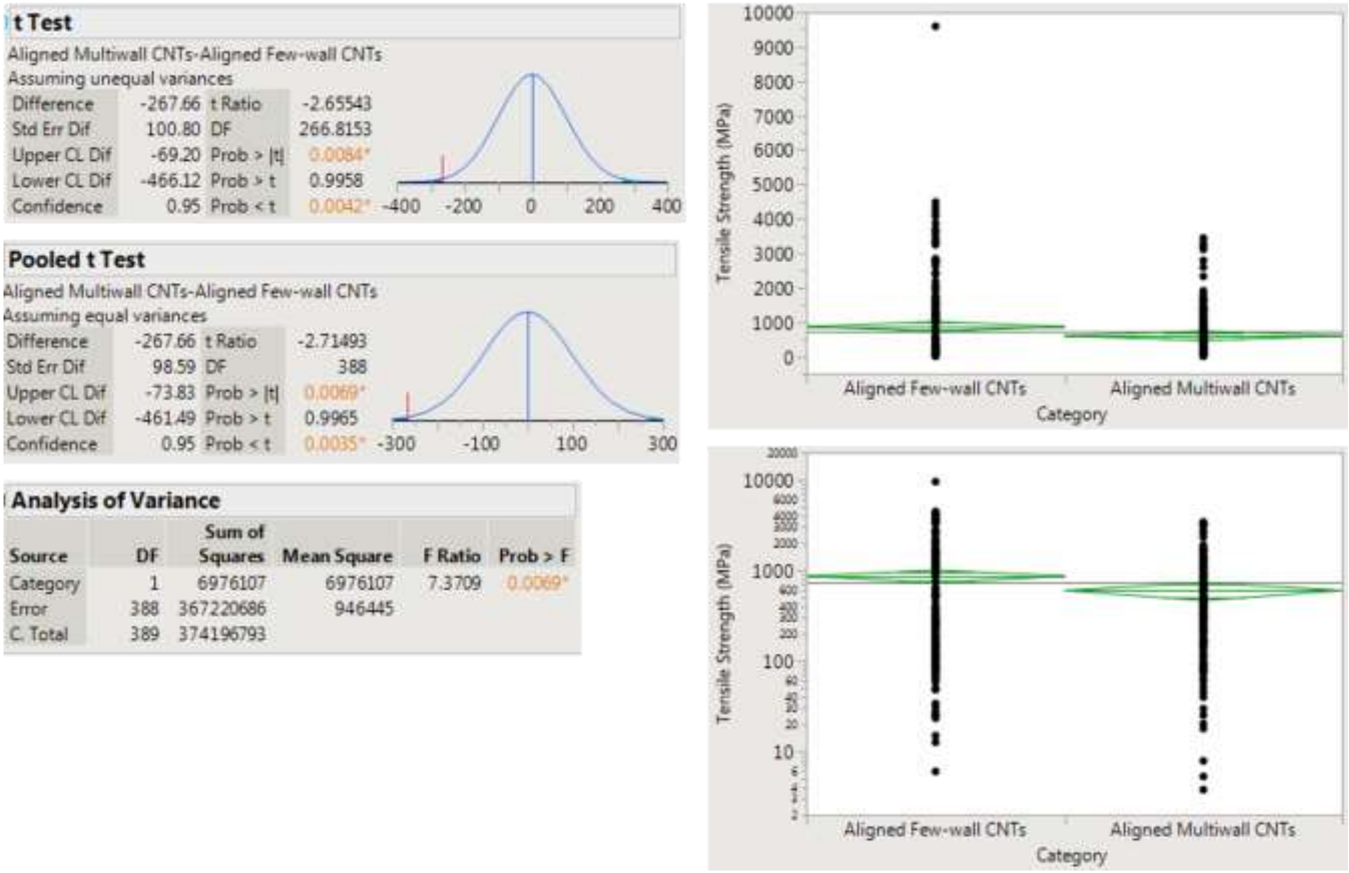

Supplemental Figure S1. t-test showing on average aligned FWCNTs are stronger than aligned MWCNTs. Note that a statistically significant difference between these populations persists even after removing the stratified data point near 10 GPa.

ANOVA and t-test comparing strengths of the two leading varieties of aligned
FWCNT material, fibers produced by the direct extraction from floating catalyst CVD (DS-CNT) and fibers produced from extrusion of a CNT liquid solution (SS-CNT). In this analysis, we consider only aligned FWCNT materials. Further, for the SS-CNT group where there are multiple solution methods, we restrict this analysis to just samples with acid/ doping exposure. Acid-based SS-CNTs are well known to yield the best properties of SS-CNT and makes a more fair comparison of averages between DS-CNT and SS-CNT.

t Test

Liquid Extrusion-Floating Catalyst
Assuming unequal variances

Difference	89.29	t Ratio	0.325733
Std Err Dif	274.12	DF	101.8176
Upper CL Dif	633.02	Prob > |t|	0.7453
Lower CL Dif	-454.44	Prob > t	0.3726
Confidence	0.95	Prob < t	0.6274

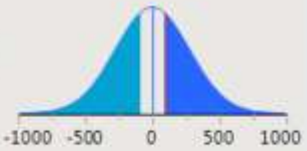

Pooled t Test

Liquid Extrusion-Floating Catalyst
Assuming equal variances

Difference	89.29	t Ratio	0.304947
Std Err Dif	292.81	DF	102
Upper CL Dif	670.07	Prob > |t|	0.7610
Lower CL Dif	-491.49	Prob > t	0.3805
Confidence	0.95	Prob < t	0.6195

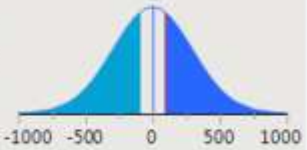

Analysis of Variance

Source	DF	Sum of Squares	Mean Square	F Ratio	Prob > F
Production Process	1	201084	201084	0.0930	0.7610
Error	102	220560864	2162361		
C. Total	103	220761948			

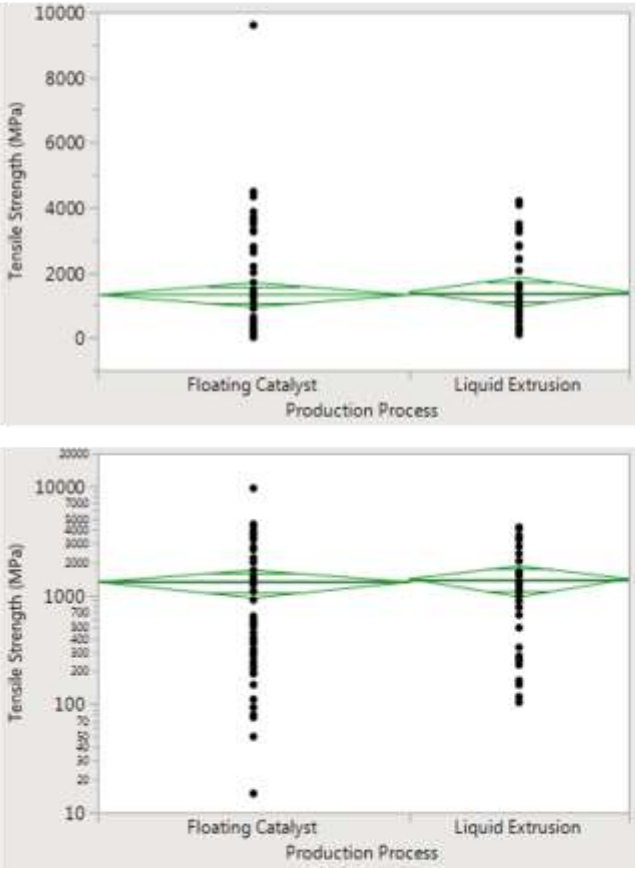

Supplemental Figure S2. t-test showing, on average, the tensile strength of fibres derived from direct FC-CVD extraction (DS-CNT) and fibres derived from extrusion of super-acid CNT solutions (SS-CNT) are equal. Here we only considered the aligned FWCNT subsets. Further, we restricted the SS-CNT subset to those with doping/ acid exposure, the most favourable embodiment of SS-CNTs.

Here we test the on/ off effect of doping on strength, for several different CNT categories.

ANOVA and t-test comparing strengths of aligned MWCNT material, with and without doping.

t Test

Yes-No

Assuming unequal variances

Difference	59.31	t Ratio	0.484266
Std Err Dif	122.46	DF	32.58449
Upper CL Dif	308.58	Prob > |t|	0.6314
Lower CL Dif	-189.97	Prob > t	0.3157
Confidence	0.95	Prob < t	0.6843

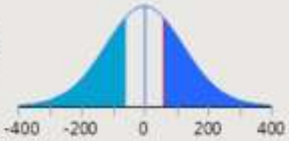

Pooled t Test

Yes-No

Assuming equal variances

Difference	59.31	t Ratio	0.454149
Std Err Dif	130.59	DF	200
Upper CL Dif	316.81	Prob > |t|	0.6502
Lower CL Dif	-198.20	Prob > t	0.3251
Confidence	0.95	Prob < t	0.6749

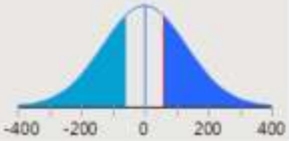

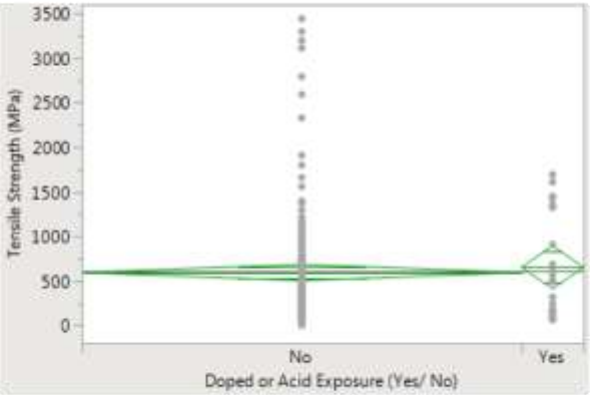

Analysis of Variance

Source	DF	Sum of Squares	Mean Square	F Ratio	Prob > F
Doped or Acid Exposure (Yes/ No)	1	77045	77045	0.2063	0.6502
Error	200	74710125	373551		
C. Total	201	74787170			

Supplemental Figure S3. t-test showing no strength difference from doping on MWCNT materials.

ANOVA and t-test comparing strengths of aligned FWCNT material extracted directly from Floating Catalyst CVD (DS-CNT), with and without exposure to doping or acids.

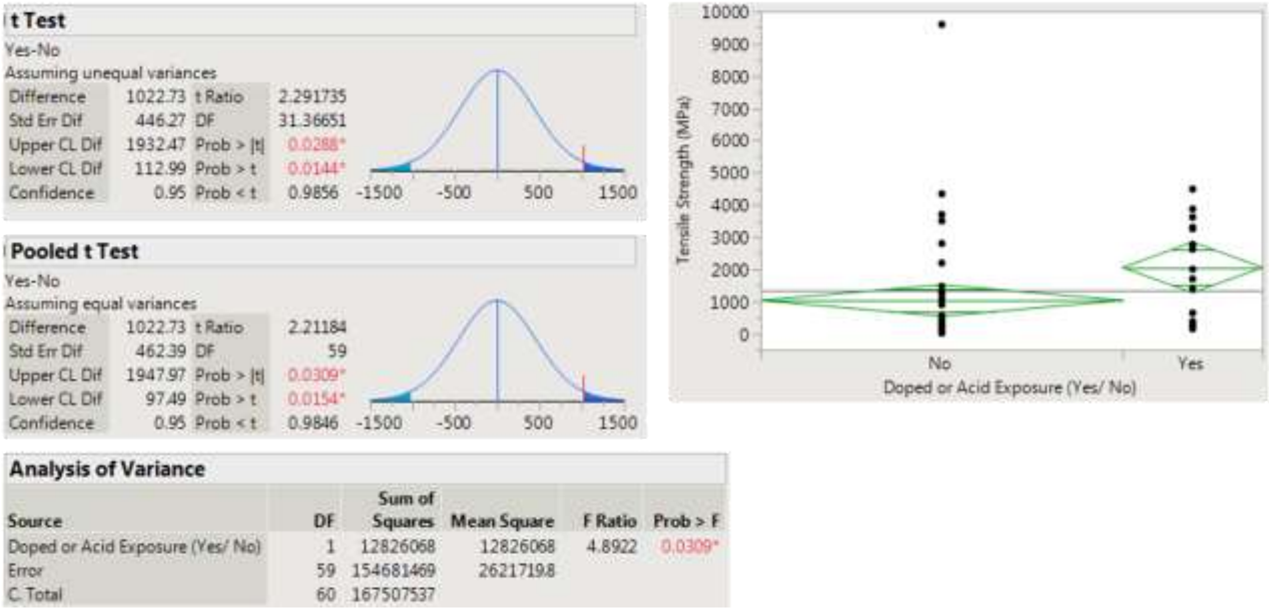

t Test

Yes-No

Assuming unequal variances

Difference	1022.73	t Ratio	2.291735
Std Err Dif	446.27	DF	31.36651
Upper CL Dif	1932.47	Prob > |t|	0.0288*
Lower CL Dif	112.99	Prob > t	0.0144*
Confidence	0.95	Prob < t	0.9856

Pooled t Test

Yes-No

Assuming equal variances

Difference	1022.73	t Ratio	2.21184
Std Err Dif	462.39	DF	59
Upper CL Dif	1947.97	Prob > |t|	0.0309*
Lower CL Dif	97.49	Prob > t	0.0154*
Confidence	0.95	Prob < t	0.9846

Analysis of Variance

Source	DF	Sum of Squares	Mean Square	F Ratio	Prob > F
Doped or Acid Exposure (Yes/ No)	1	12826068	12826068	4.8922	0.0309*
Error	59	154681469	2621719.8		
C. Total	60	167507537			

Supplemental Figure S4. t-test showing doped aligned FWCNT materials are stronger than undoped. This is just from CNT materials extracted directly from FC-CVD (DS-CNT).

ANOVA and t-test comparing strengths of aligned FWCNT material from extrusion from liquids (SS-CNT), with and without exposure to doping or acids.

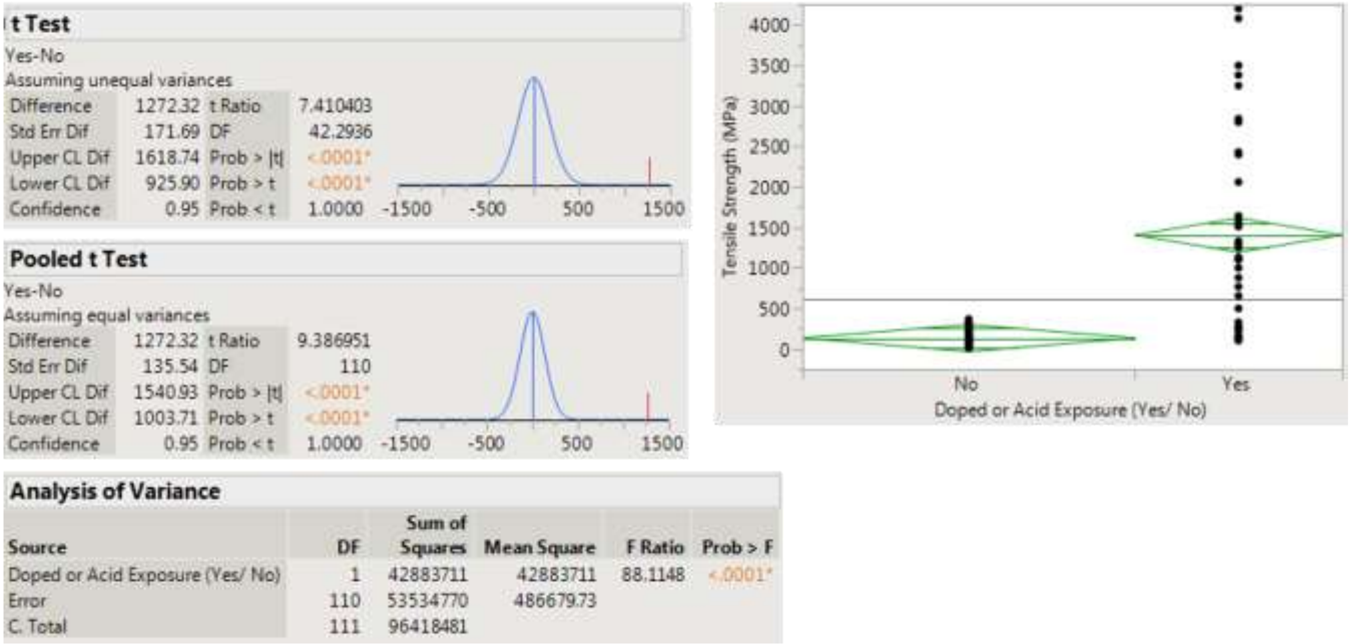

t Test

Yes-No

Assuming unequal variances

Difference	1272.32	t Ratio	7.410403
Std Err Dif	171.69	DF	42.2936
Upper CL Dif	1618.74	Prob > |t|	<.0001*
Lower CL Dif	925.90	Prob > t	<.0001*
Confidence	0.95	Prob < t	1.0000

Pooled t Test

Yes-No

Assuming equal variances

Difference	1272.32	t Ratio	9.386951
Std Err Dif	135.54	DF	110
Upper CL Dif	1540.93	Prob > |t|	<.0001*
Lower CL Dif	1003.71	Prob > t	<.0001*
Confidence	0.95	Prob < t	1.0000

Analysis of Variance

Source	DF	Sum of Squares	Mean Square	F Ratio	Prob > F
Doped or Acid Exposure (Yes/ No)	1	42883711	42883711	88.1148	<.0001*
Error	110	53534770	486679.73		
C. Total	111	96418481			

Supplemental Figure S5. t-test showing doped aligned FWCNT materials are stronger than undoped, for wet-spun fibres (SS-CNT). This is somewhat misleading however in that the super-acid extruded subcategory has better properties generally than other wet-spinning techniques and is typically in a doped configuration.

Now we compare the thermal conductivity of aligned MWCNT materials against aligned FWCNT materials.

ANOVA and t-test comparing thermal conductivity between aligned FWCNT material and aligned MWCNT material.

t Test

Aligned Multiwall CNTs-Aligned Few-wall CNTs
Assuming unequal variances

Difference	97.63	t Ratio	1.291624
Std Err Dif	75.59	DF	60.73595
Upper CL Dif	248.79	Prob > |t|	0.2014
Lower CL Dif	-53.53	Prob > t	0.1007
Confidence	0.95	Prob < t	0.8993

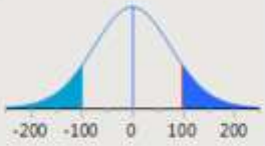

Pooled t Test

Aligned Multiwall CNTs-Aligned Few-wall CNTs
Assuming equal variances

Difference	97.63	t Ratio	1.23667
Std Err Dif	78.95	DF	68
Upper CL Dif	255.17	Prob > |t|	0.2205
Lower CL Dif	-59.91	Prob > t	0.1102
Confidence	0.95	Prob < t	0.8898

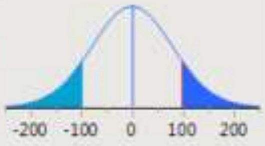

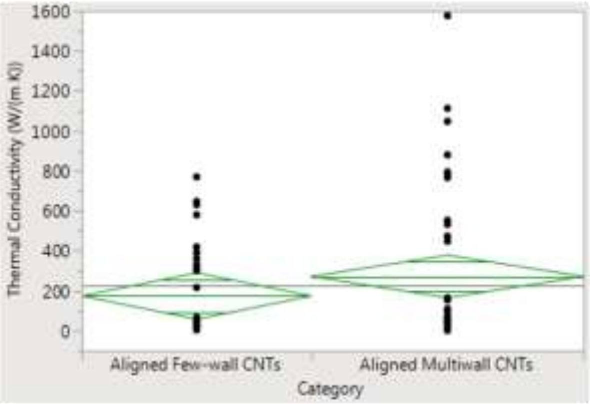

Analysis of Variance

Source	DF	Sum of Squares	Mean Square	F Ratio	Prob > F
Category	1	165583.1	165583	1.5294	0.2205
Error	68	7362367.1	108270		
C. Total	69	7527950.2			

Supplemental Figure S6. t-test showing no major difference in thermal conductivity of aligned MWCNT and FWCNT materials.

Now we compare the specific strength of wet spun and FC-CVD derived fibres.

ANOVA and t-test comparing *specific* strengths of the two leading varieties of aligned FWCNT material, fibres produced by the direct extraction from floating catalyst CVD (DS-CNT) and fibres produced from extrusion of a CNT liquid solution (SS-CNT). In this analysis, we consider only aligned FWCNT materials. Further, for the SS-CNT group where there are multiple solution methods, we restrict this analysis to just samples with acid/ doping exposure. Acid-based SS-CNTs are well known to yield the best properties of SS-CNT and makes a more fair comparison of averages between DS-CNT and SS-CNT.

t Test

Liquid Extrusion-Floating Catalyst
Assuming unequal variances

Difference	-1.0035	t Ratio	-2.89027
Std Err Dif	0.3472	DF	19.81887
Upper CL Dif	-0.2788	Prob > |t|	0.0091*
Lower CL Dif	-1.7282	Prob > t	0.9954
Confidence	0.95	Prob < t	0.0046*

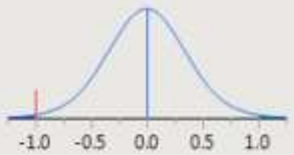

Pooled t Test

Liquid Extrusion-Floating Catalyst
Assuming equal variances

Difference	-1.0035	t Ratio	-2.0438
Std Err Dif	0.4910	DF	78
Upper CL Dif	-0.0260	Prob > |t|	0.0443*
Lower CL Dif	-1.9810	Prob > t	0.9778
Confidence	0.95	Prob < t	0.0222*

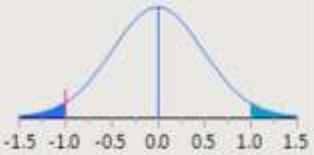

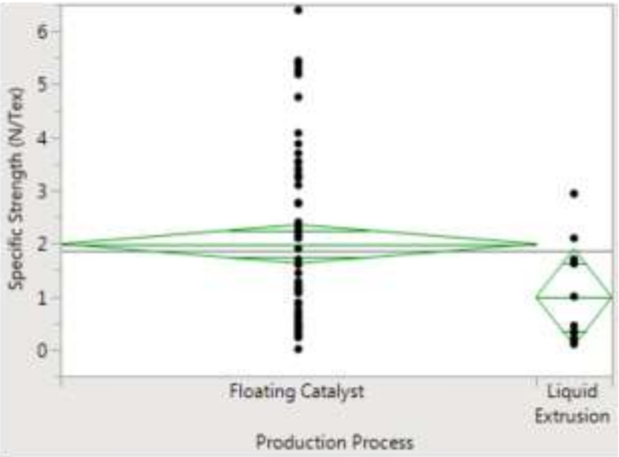

Analysis of Variance

Source	DF	Sum of Squares	Mean Square	F Ratio	Prob > F
Production Process	1	9.55405	9.55405	4.1771	0.0443*
Error	78	178.40421	2.28723		
C. Total	79	187.95826			

Supplemental Figure S7. t-test showing fibres derived directly from FC-CVD (DS-CNT) are decisively stronger than wet-spun fibres (SS-CNT) on the basis of weight. Again for the SS-CNT fibres, we only consider those with acid exposure because it is the most favourable embodiment of SS-CNT methods.

Here we show the correlation and power law of electrical conductivity and tensile strength against the CNT diameter (with and without the weighted adjustment) of all four bulk CNT categories grouped together (unaligned MWCNT, aligned MWCNT, unaligned FWCNT, aligned FWCNT).

Log Electrical Conductivity vs Log CNT diameter

	Correlation	Fitted Slope for Power Law	Fitted Slope Standard Error	Correlation and Fit Probability	Adjusted Correlation	Adjusted Fitted Slope for Power Law	Adjusted Fitted Slope Standard Error	Adjusted Correlation and Fit Probability	Number of data points
All four CNT categories	**-0.34**	**-0.63**	**0.09**	**<0.01**	**-0.39**	**-0.73**	**0.08**	**<0.01**	408

Log Tensile Strength vs Log CNT diameter

	Correlation	Fitted Slope for Power Law	Fitted Slope Standard Error	Correlation and Fit Probability	Adjusted Correlation	Adjusted Fitted Slope for Power Law	Adjusted Fitted Slope Standard Error	Adjusted Correlation and Fit Probability	Number of data points
All four CNT categories	**-0.21**	**-0.26**	**0.07**	**<0.01**	**-0.32**	**-0.46**	**0.08**	**<0.01**	271

Supplemental Figure S8. Tables quantifying the relationship of physical properties against CNT diameter, where all four categories have been grouped together.
